# Supplementary material for: Crosstalk between chromatin state and ATM signalling in DNA damage-induced transcription stress
Source: EMBO J. 2025 Aug 26;44(19):5564–94. doi: 10.1038/s44318-025-00537-7 (PMC12489091; doi:10.1038/s44318-025-00537-7)
Supplement: Supplementary file 2 — Source data Fig. 1 [file 44318_2025_537_MOESM2_ESM.zip › EMBOJ-2025-120849-T_Source data Fig_1/Fig_1A/readme_Fig_1A.docx]

**Immunofluorescence detection of histone H3 acetylation (H3Ac) in untreated and UV-irradiated cells (Figure 1A)**

**Folder Contents:**
This folder contains microscopy images (“Images” subfolder) and numerical data (Excel file) corresponding to the quantifications shown in Figure 1A of the manuscript.

**Image Folder:**

- Confocal microscopy images were acquired at a resolution of 512 × 512 pixels using a Zeiss LSM700 laser-scanning confocal microscope.
- Images were exported as TIFF files directly from ZEN software, with acquisition settings optimized for high-throughput imaging across multiple conditions.
- No image processing, filtering, or resolution downsampling was applied to the TIFF files after acquisition.
- For figure presentation, brightness and contrast were adjusted uniformly across all conditions to enhance visualization. These adjustments were made only to representative figure panels, not the files used for quantification.
- Images displayed in the figure panel were cropped for clarity. Cropped regions are indicated in a separate merged image file included for reference.

**Numerical Data File:**

- Signal intensities quantified using Fiji (ImageJ), were normalized to the average value of non-irradiated cells.
- Graphs were generated using GraphPad Prism, and all statistical analyses were also performed in Prism, as detailed in the Excel file.

**Image Handling Notes:**
All measurements were based on original unmodified images. Adjustments to figure panels were for illustration purposes only and applied uniformly across samples.
