# Supplementary material for: Crosstalk between chromatin state and ATM signalling in DNA damage-induced transcription stress
Source: EMBO J. 2025 Aug 26;44(19):5564–94. doi: 10.1038/s44318-025-00537-7 (PMC12489091; doi:10.1038/s44318-025-00537-7)
Supplement: Supplementary file 2 — Source data Fig. 1 [file 44318_2025_537_MOESM2_ESM.zip › EMBOJ-2025-120849-T_Source data Fig_1/Fig_1B/readme_Fig_1B.docx]

**Immunoblots showing histone H3 acetylation in UV-irradiated cells (Figure 1B)**

**Folder Contents:**
This folder contains the source data for the immunoblots presented in Figure 1B of the manuscript, including TIFF images (“Images” subfolder) and an Excel file with quantification and reference panels.

**Image Acquisition:**

- Immunoblot images were acquired using an Odyssey CLx (LI-COR) imaging system.
- Image intensity levels were adjusted prior to quantification to ensure grayscale rendering and avoid saturation. These adjustments were applied uniformly across the membrane and did not alter the relative signal intensities.
- Images were exported as TIFFs directly from the LI-COR Image Studio 6.0 Software.

**Blotting and Antibody Incubation Details:**

- Membranes were cut prior to antibody incubation to allow probing with different antibodies.
- Acetylated histone H3 and total histone H3 were detected on the same membrane, sequentially.

**Excel File Contents:**
The Excel file includes:

- Quantified signal intensities calculated using ImageQuant software.
- Normalization to total histone H3 signal.
- Fold change values relative to non-irradiated control samples.
- Cropped blot image corresponding to the panel shown in Figure 1B (included for reference only).
- Identifiers matching each cropped region to its respective uncropped TIFF image.

**Image Handling Notes:**
All quantifications were performed on grayscale-adjusted, unsaturated TIFF images. Cropped versions were used exclusively for figure presentation and were not used for data analysis.
