# Supplementary material for: Crosstalk between chromatin state and ATM signalling in DNA damage-induced transcription stress
Source: EMBO J. 2025 Aug 26;44(19):5564–94. doi: 10.1038/s44318-025-00537-7 (PMC12489091; doi:10.1038/s44318-025-00537-7)
Supplement: Supplementary file 2 — Source data Fig. 1 [file 44318_2025_537_MOESM2_ESM.zip › EMBOJ-2025-120849-T_Source data Fig_1/Fig_1C/readme_Fig_1C.docx]

**Immunoblots showing histone H3 acetylation in the chromatin fraction and in CSB-associated immunoprecipitated mononucleosomes (Figure 1C)**

**Folder Contents:**
This folder contains TIFF images (“Images” subfolder) and an Excel file corresponding to the immunoblots presented in Figure 1C of the manuscript.

**Image Acquisition:**

- Immunoblot images were acquired using an Odyssey CLx (*LI-COR)* imaging system.
- Image intensity levels were adjusted prior to quantification to ensure grayscale rendering and avoid saturation. These adjustments were applied uniformly across the membrane and did not alter the relative signal intensities.
- Images were exported as TIFFs directly from the *LI-COR* Image Studio 6.0 Software.

**Blotting and Antibody Incubation Details:**

- Membranes were cut prior to antibody incubation to enable separate hybridization with different antibodies.
- All shown proteins were detected on the same membrane. Acetylated and total histone H3 were detected by probing the membrane sequentially.

**Excel File:**
The Excel file includes:

- Quantified signal intensities obtained using ImageQuant software.
- Normalization to total histone H3 levels.
- Fold change calculations relative to non-irradiated controls.
- Representative figure panel (cropped images) corresponding to the final Figure 1C layout (included for reference only).
- Identifiers matching each cropped region to its respective uncropped TIFF image.

**Image Handling Notes:**
All quantifications were performed on grayscale-adjusted, unsaturated TIFF images. Cropped versions were used exclusively for figure presentation and were not used for data analysis.
