# Supplementary material for: Crosstalk between chromatin state and ATM signalling in DNA damage-induced transcription stress
Source: EMBO J. 2025 Aug 26;44(19):5564–94. doi: 10.1038/s44318-025-00537-7 (PMC12489091; doi:10.1038/s44318-025-00537-7)
Supplement: Supplementary file 2 — Source data Fig. 1 [file 44318_2025_537_MOESM2_ESM.zip › EMBOJ-2025-120849-T_Source data Fig_1/Fig_1E/readme_Fig_1E.docx]

**Splicing factor mobility measured by FRAP in untreated and UV-C–irradiated cells ± Trichostatin A (TSA) (Figure 1E)**

**File Description:**
This file contains the numerical data corresponding to the FRAP analysis shown in Figure 1E of the manuscript.

**Experimental Details:**
The mobility of GFP-tagged splicing factors was assessed using strip-FRAP on a Leica TCS SP5 AOBS laser scanning confocal microscope.

**Data Acquisition and Quantification:**

- Images were acquired and quantified using LASAF software.
- Fluorescence recovery was calculated in Microsoft Excel by subtracting background fluorescence (measured outside the bleached strip) from the fluorescence intensity in the bleached region, followed by normalization to pre-bleach levels.

**Data Presented:**

- The file contains averaged FRAP values from images acquired 20–21 seconds post-photobleaching (~100 images per condition).
- Values for treated conditions are presented after subtraction of the mean values from untreated controls.

**Image Processing:**

All analyses were performed on raw, unmodified images. No post-acquisition image processing or resolution downsampling was applied.

**Graphing and Statistical Analysis:**

Graphs were generated using GraphPad Prism. Statistical analyses were also performed in Prism, as detailed in the accompanying Excel file.
