# Supplementary material for: Crosstalk between chromatin state and ATM signalling in DNA damage-induced transcription stress
Source: EMBO J. 2025 Aug 26;44(19):5564–94. doi: 10.1038/s44318-025-00537-7 (PMC12489091; doi:10.1038/s44318-025-00537-7)
Supplement: Supplementary file 2 — Source data Fig. 1 [file 44318_2025_537_MOESM2_ESM.zip › EMBOJ-2025-120849-T_Source data Fig_1/Fig_1H/readme_Fig_1H.docx]

**Displacement of SNRNP40 and SF3a1 from UV-C laser–irradiated subnuclear regions (Figure 1H)**

**File Description:**
The Excel file contains the numerical data corresponding to the analysis presented in Figure 1H of the manuscript.

**Experimental Details:**
Displacement of GFP-tagged SNRNP40 and SF3a1 was analyzed following UV-C laser microirradiation in live cells. Imaging was performed using a Leica TCS SP5 AOBS laser scanning confocal microscope.

**Data Acquisition and Quantification:**

- Images were acquired and quantified using LASAF software.
- Raw numerical data were processed in Excel. For each time point and individual cell, the data represent:
  *(Fluorescence intensity in the UV-C–irradiated region − nuclear background fluorescence), normalized to pre-irradiation fluorescence levels.*

**Image Processing:**
All quantifications were performed on unmodified, raw images. No image processing or resolution downsampling was applied post-acquisition.

**Graphing and Statistical Analysis:**
Graphs were generated in GraphPad Prism. Statistical analyses were also performed using Prism, as described in the accompanying Excel file.
