# Supplementary figures and images for: Crosstalk between chromatin state and ATM signalling in DNA damage-induced transcription stress (part 1 of 2)
Source: EMBO J. 2025 Aug 26;44(19):5564–94. doi: 10.1038/s44318-025-00537-7 (PMC12489091; doi:10.1038/s44318-025-00537-7)

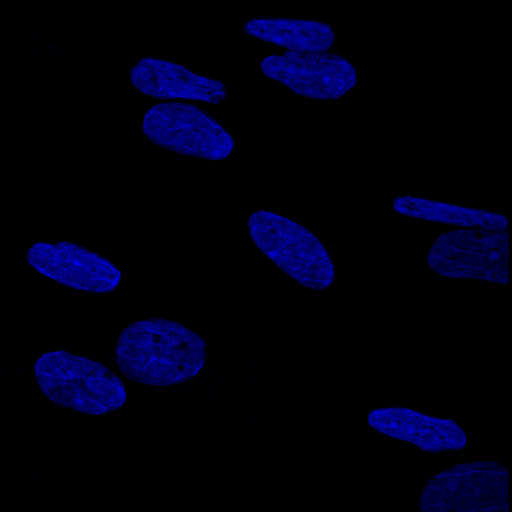

Supplement: Supplementary file 2 — Source data Fig. 1 [file 44318_2025_537_MOESM2_ESM.zip › EMBOJ-2025-120849-T_Source data Fig_1/Fig_1A/Images_Fig_1A/DAPI_untreated.tif]

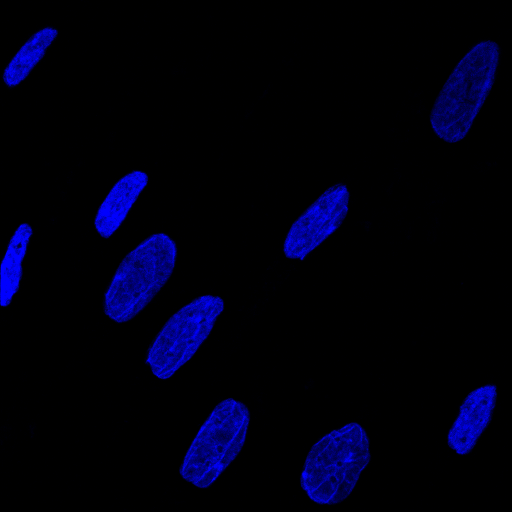

Supplement: Supplementary file 2 — Source data Fig. 1 [file 44318_2025_537_MOESM2_ESM.zip › EMBOJ-2025-120849-T_Source data Fig_1/Fig_1A/Images_Fig_1A/DAPI_UV.tif]

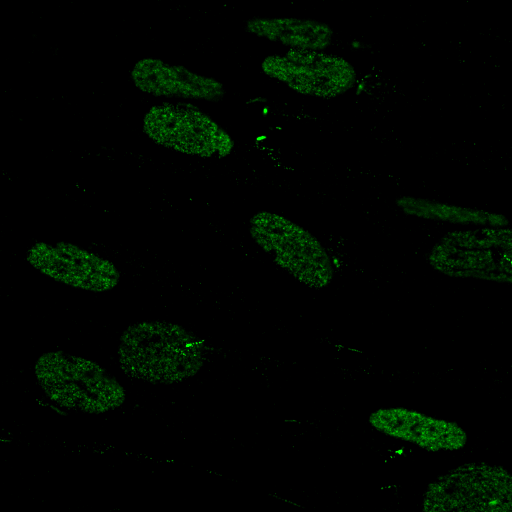

Supplement: Supplementary file 2 — Source data Fig. 1 [file 44318_2025_537_MOESM2_ESM.zip › EMBOJ-2025-120849-T_Source data Fig_1/Fig_1A/Images_Fig_1A/H3Ac_untreated.tif]

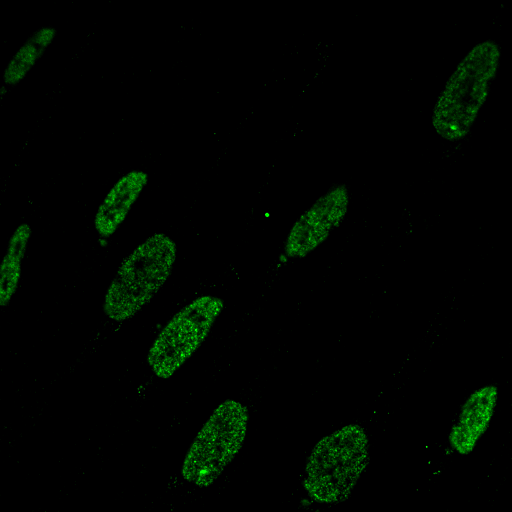

Supplement: Supplementary file 2 — Source data Fig. 1 [file 44318_2025_537_MOESM2_ESM.zip › EMBOJ-2025-120849-T_Source data Fig_1/Fig_1A/Images_Fig_1A/H3Ac_UV.tif]

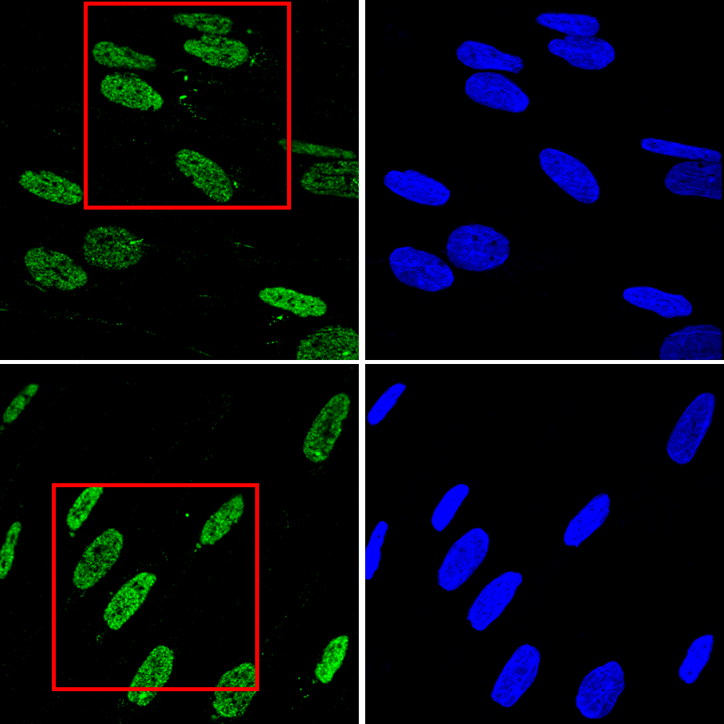

Supplement: Supplementary file 2 — Source data Fig. 1 [file 44318_2025_537_MOESM2_ESM.zip › EMBOJ-2025-120849-T_Source data Fig_1/Fig_1A/Images_Fig_1A/Cropped_image_areas_Fig_1A.tif]

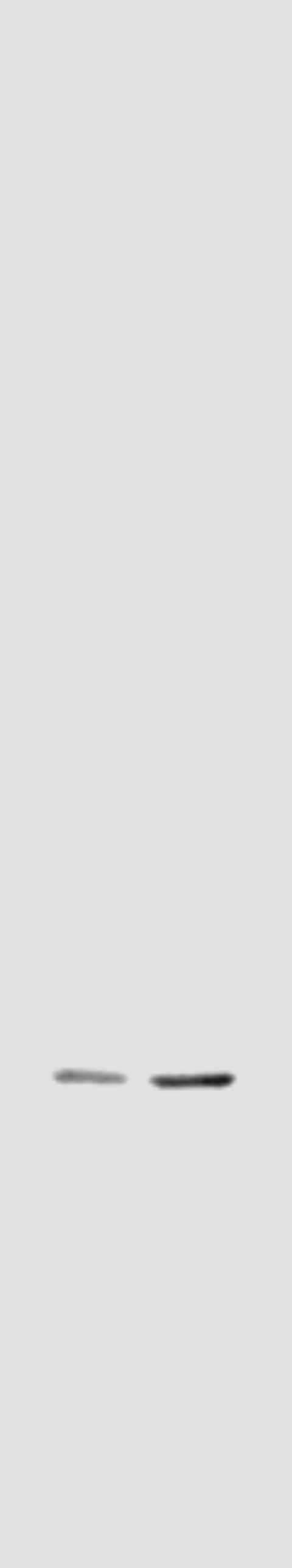

Supplement: Supplementary file 2 — Source data Fig. 1 [file 44318_2025_537_MOESM2_ESM.zip › EMBOJ-2025-120849-T_Source data Fig_1/Fig_1B/Images_Fig_1B/H3Ac.tif]

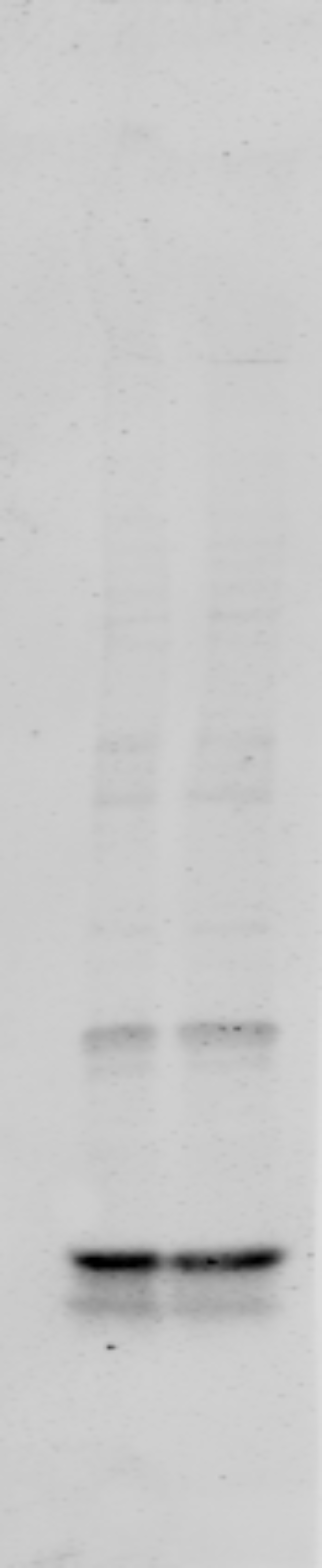

Supplement: Supplementary file 2 — Source data Fig. 1 [file 44318_2025_537_MOESM2_ESM.zip › EMBOJ-2025-120849-T_Source data Fig_1/Fig_1B/Images_Fig_1B/Histone_H3.tif]

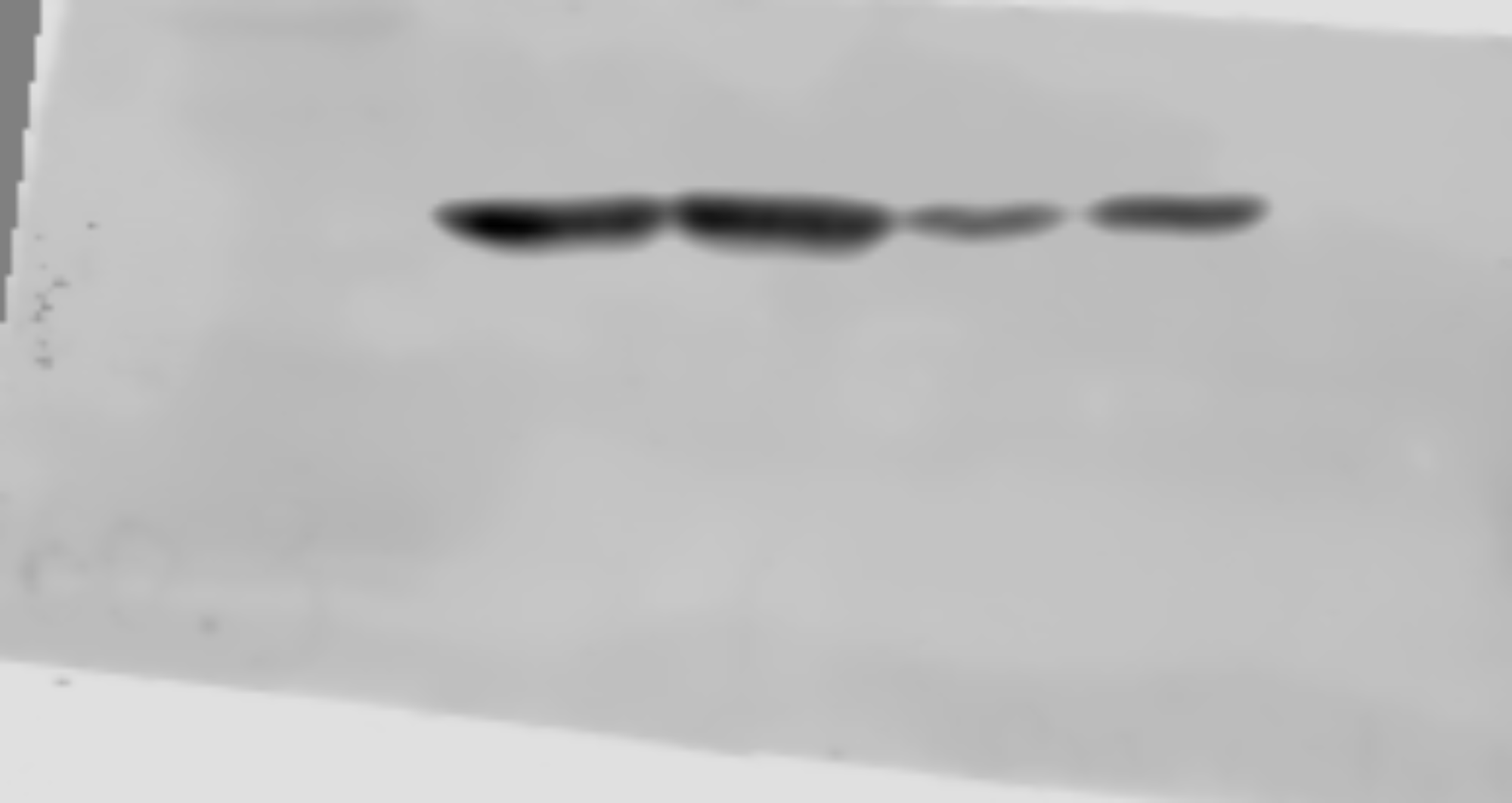

Supplement: Supplementary file 2 — Source data Fig. 1 [file 44318_2025_537_MOESM2_ESM.zip › EMBOJ-2025-120849-T_Source data Fig_1/Fig_1C/Images_Fig_1C/Histone_H3.tif]

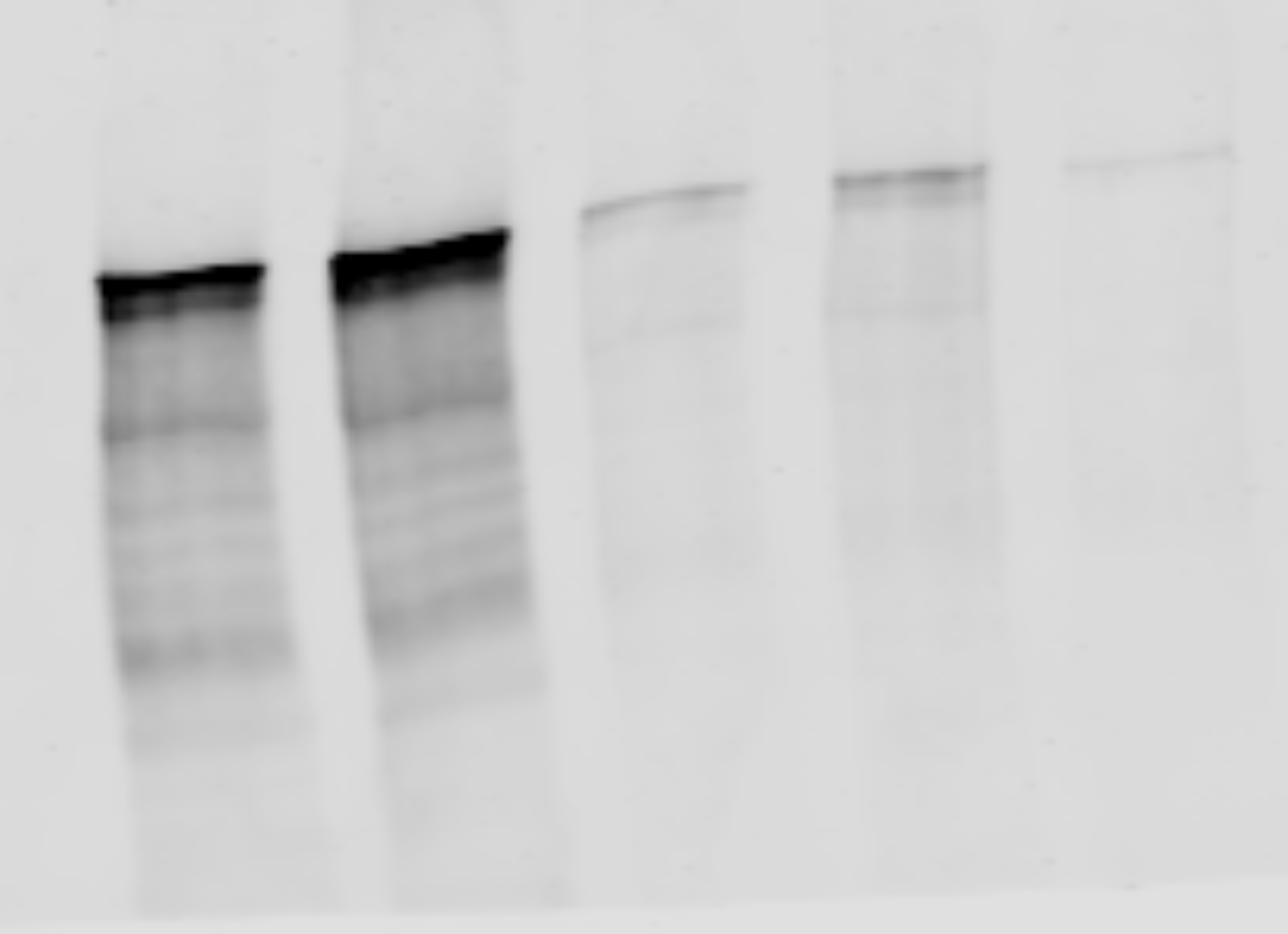

Supplement: Supplementary file 2 — Source data Fig. 1 [file 44318_2025_537_MOESM2_ESM.zip › EMBOJ-2025-120849-T_Source data Fig_1/Fig_1C/Images_Fig_1C/RPB1(S2P).tif]

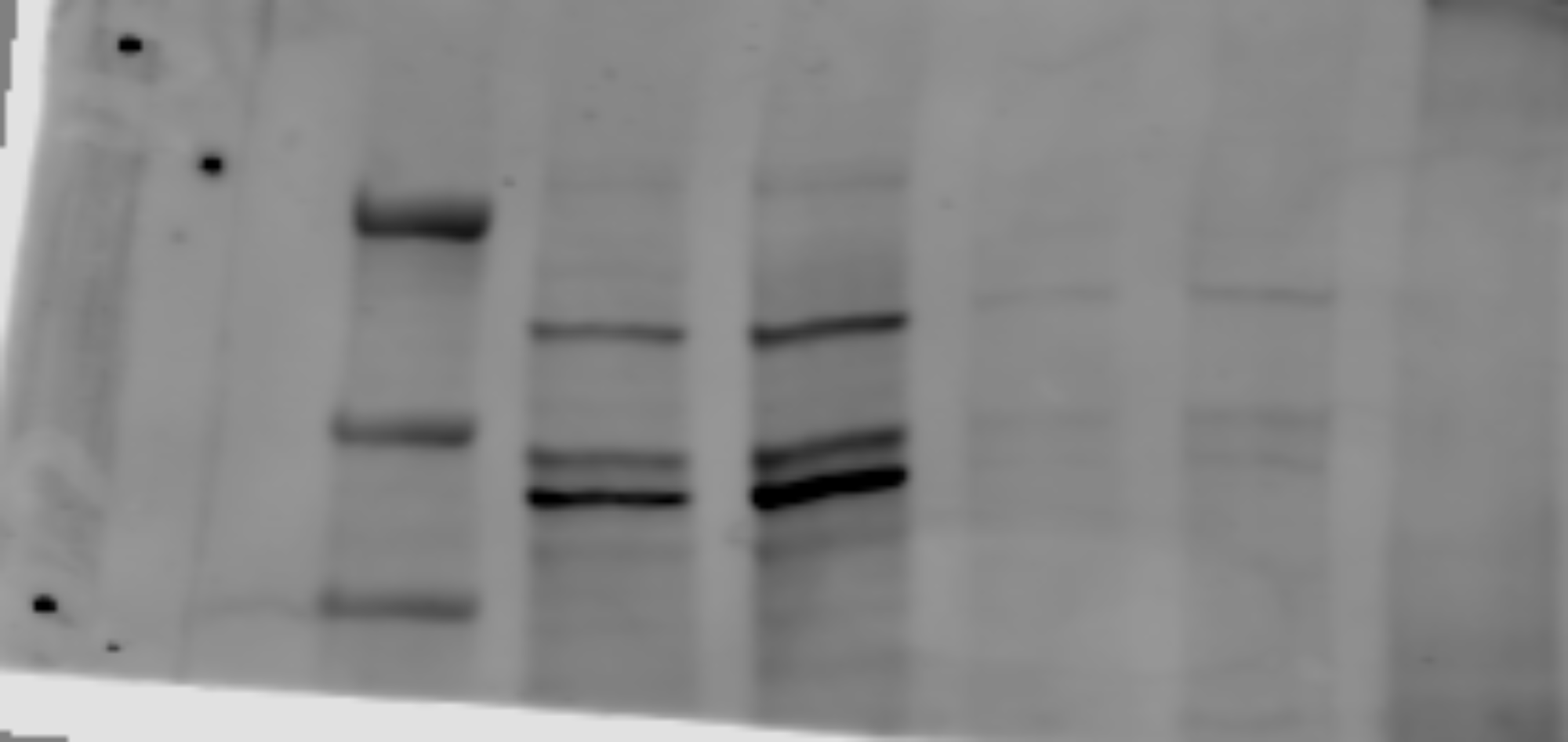

Supplement: Supplementary file 2 — Source data Fig. 1 [file 44318_2025_537_MOESM2_ESM.zip › EMBOJ-2025-120849-T_Source data Fig_1/Fig_1C/Images_Fig_1C/CSB.tif]

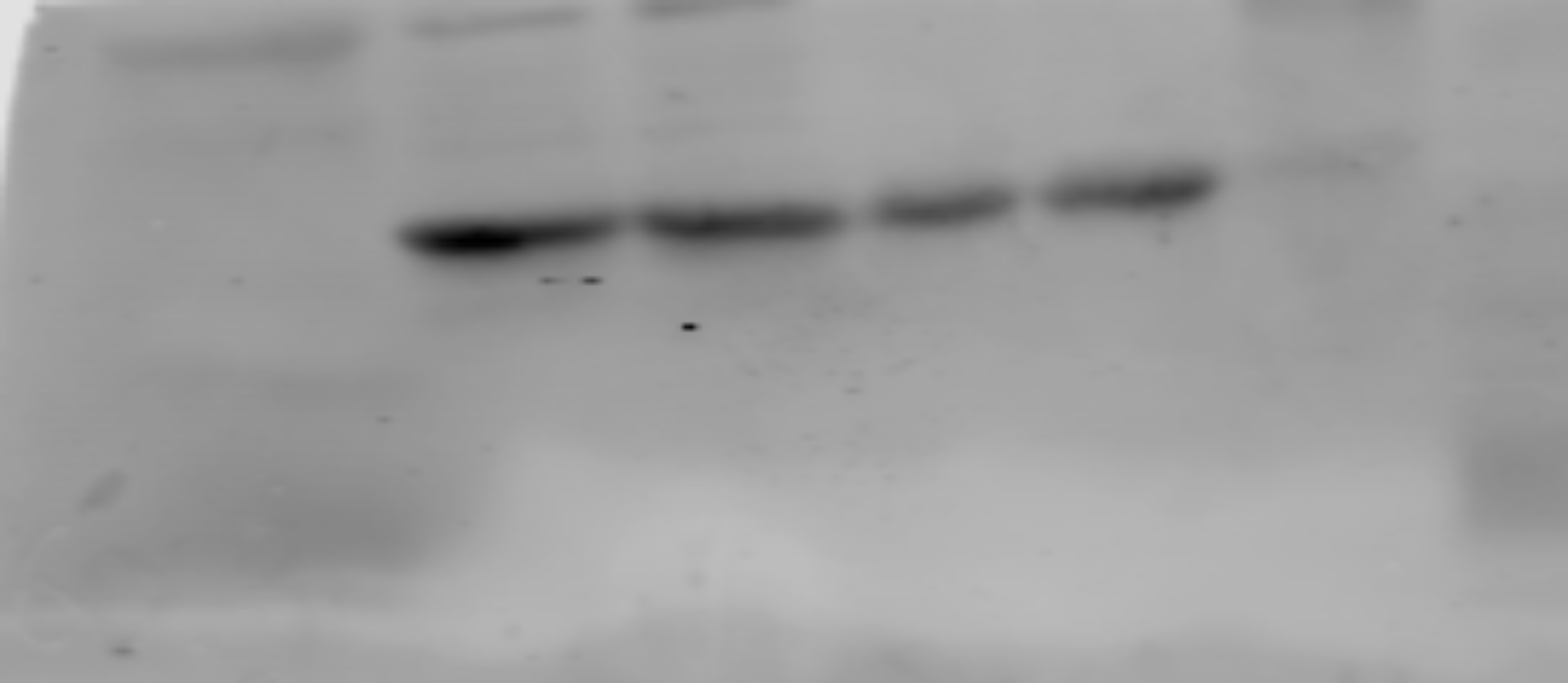

Supplement: Supplementary file 2 — Source data Fig. 1 [file 44318_2025_537_MOESM2_ESM.zip › EMBOJ-2025-120849-T_Source data Fig_1/Fig_1C/Images_Fig_1C/H3Ac.tif]

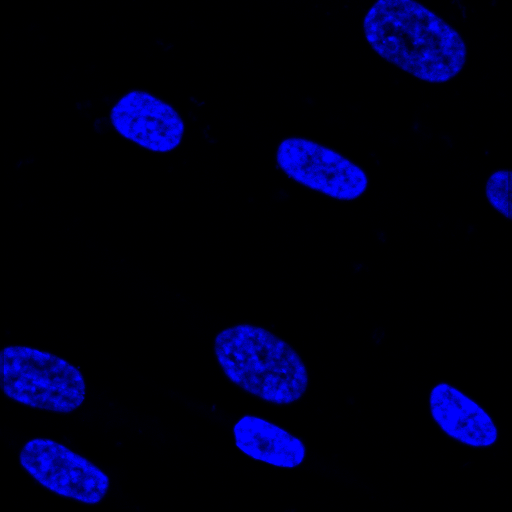

Supplement: Supplementary file 4 — Source data Fig. 3 [file 44318_2025_537_MOESM4_ESM.zip › EMBOJ-2025-120849-T_Source data Fig_3/Fig_3A/PLA_PCAF_CPD/DAPI_untreated.tif]

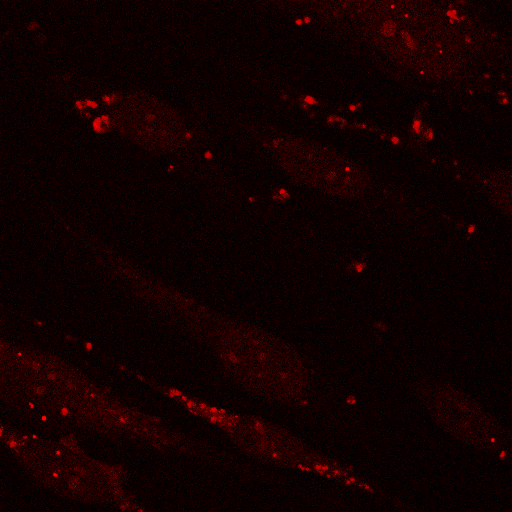

Supplement: Supplementary file 4 — Source data Fig. 3 [file 44318_2025_537_MOESM4_ESM.zip › EMBOJ-2025-120849-T_Source data Fig_3/Fig_3A/PLA_PCAF_CPD/PLA_untreated.tif]

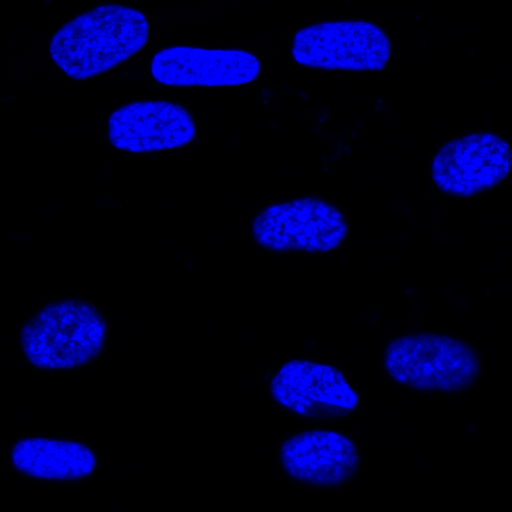

Supplement: Supplementary file 4 — Source data Fig. 3 [file 44318_2025_537_MOESM4_ESM.zip › EMBOJ-2025-120849-T_Source data Fig_3/Fig_3A/PLA_PCAF_CPD/DAPI_UV.tif]

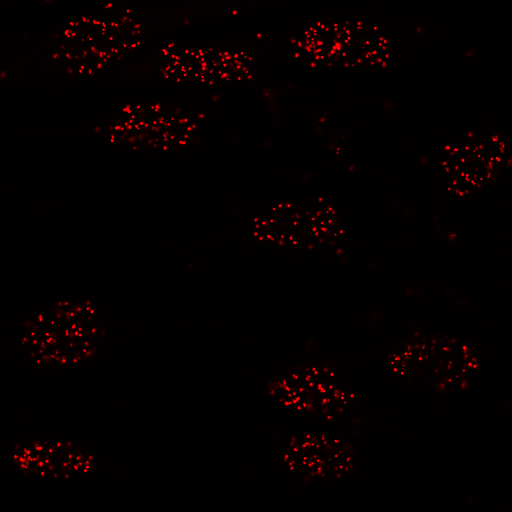

Supplement: Supplementary file 4 — Source data Fig. 3 [file 44318_2025_537_MOESM4_ESM.zip › EMBOJ-2025-120849-T_Source data Fig_3/Fig_3A/PLA_PCAF_CPD/PLA_UV.tif]

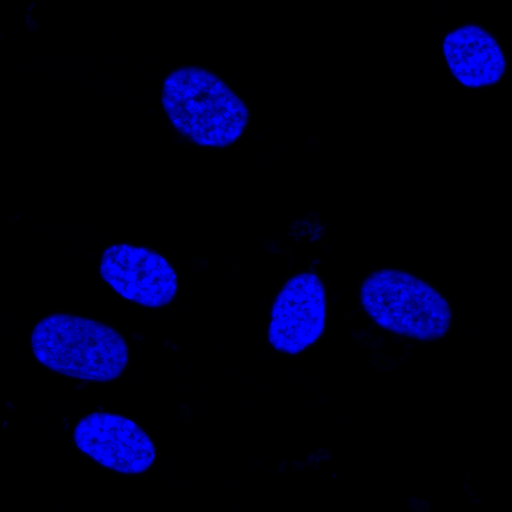

Supplement: Supplementary file 4 — Source data Fig. 3 [file 44318_2025_537_MOESM4_ESM.zip › EMBOJ-2025-120849-T_Source data Fig_3/Fig_3A/PLA_GCN5_CPD/DAPI_untreated.tif]

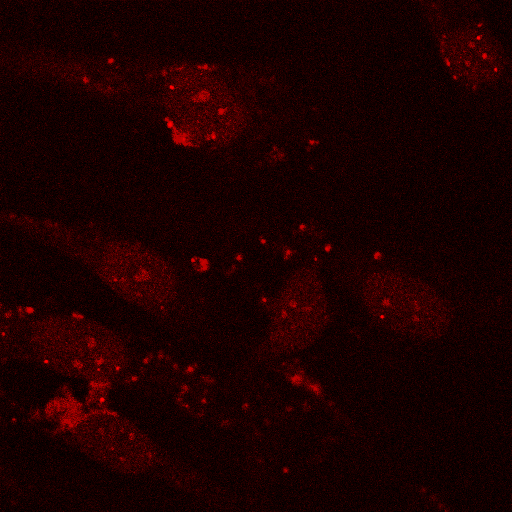

Supplement: Supplementary file 4 — Source data Fig. 3 [file 44318_2025_537_MOESM4_ESM.zip › EMBOJ-2025-120849-T_Source data Fig_3/Fig_3A/PLA_GCN5_CPD/PLA_untreated.tif]

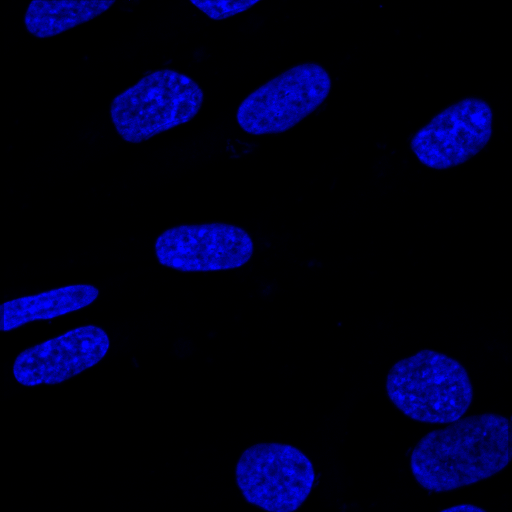

Supplement: Supplementary file 4 — Source data Fig. 3 [file 44318_2025_537_MOESM4_ESM.zip › EMBOJ-2025-120849-T_Source data Fig_3/Fig_3A/PLA_GCN5_CPD/DAPI_UV.tif]

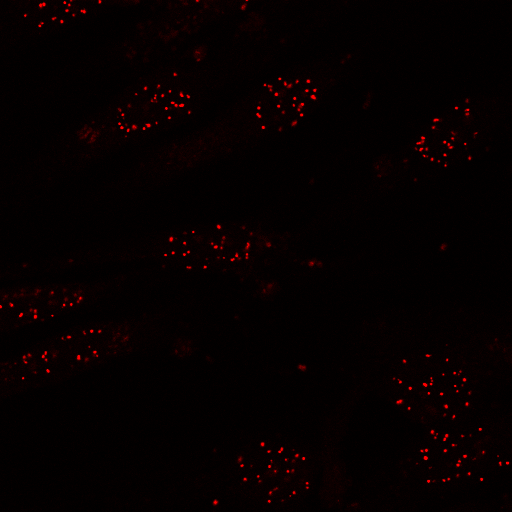

Supplement: Supplementary file 4 — Source data Fig. 3 [file 44318_2025_537_MOESM4_ESM.zip › EMBOJ-2025-120849-T_Source data Fig_3/Fig_3A/PLA_GCN5_CPD/PLA_UV.tif]

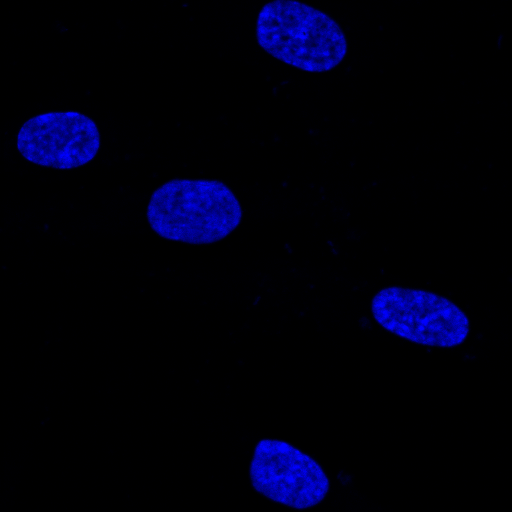

Supplement: Supplementary file 4 — Source data Fig. 3 [file 44318_2025_537_MOESM4_ESM.zip › EMBOJ-2025-120849-T_Source data Fig_3/Fig_3A/PLA_p300_CPD/DAPI_untreated.tif]

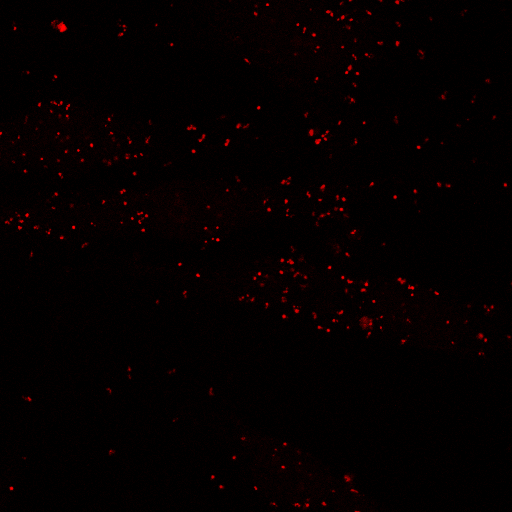

Supplement: Supplementary file 4 — Source data Fig. 3 [file 44318_2025_537_MOESM4_ESM.zip › EMBOJ-2025-120849-T_Source data Fig_3/Fig_3A/PLA_p300_CPD/PLA_untreated.tif]

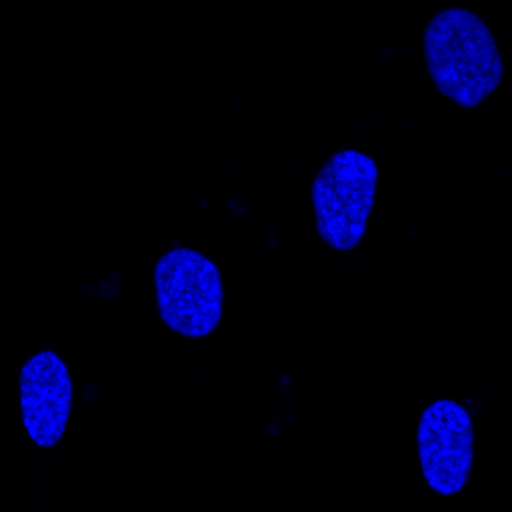

Supplement: Supplementary file 4 — Source data Fig. 3 [file 44318_2025_537_MOESM4_ESM.zip › EMBOJ-2025-120849-T_Source data Fig_3/Fig_3A/PLA_p300_CPD/DAPI_UV.tif]

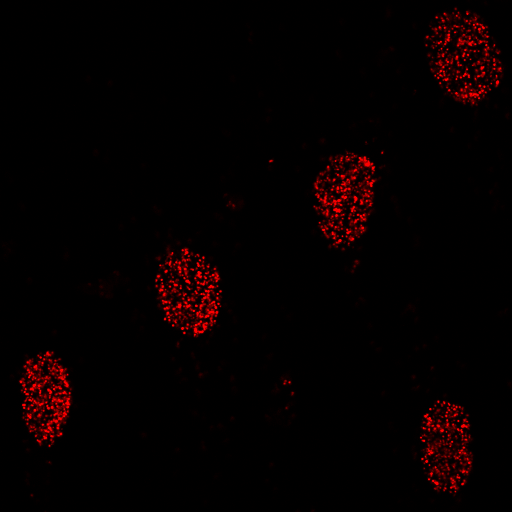

Supplement: Supplementary file 4 — Source data Fig. 3 [file 44318_2025_537_MOESM4_ESM.zip › EMBOJ-2025-120849-T_Source data Fig_3/Fig_3A/PLA_p300_CPD/PLA_UV.tif]

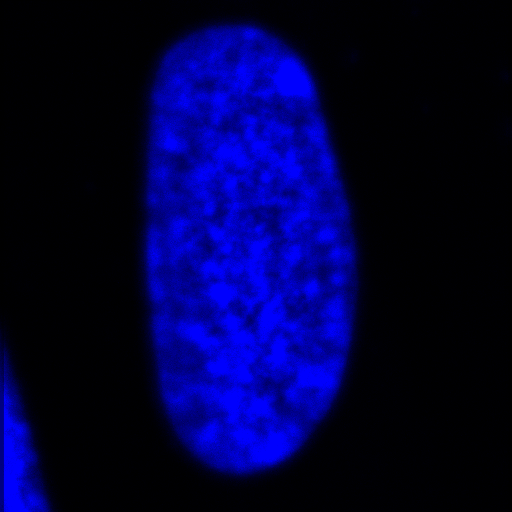

Supplement: Supplementary file 4 — Source data Fig. 3 [file 44318_2025_537_MOESM4_ESM.zip › EMBOJ-2025-120849-T_Source data Fig_3/Fig_3B/Images_Fig_3B/DAPI_UV_dose_10_magnifcation.tif]

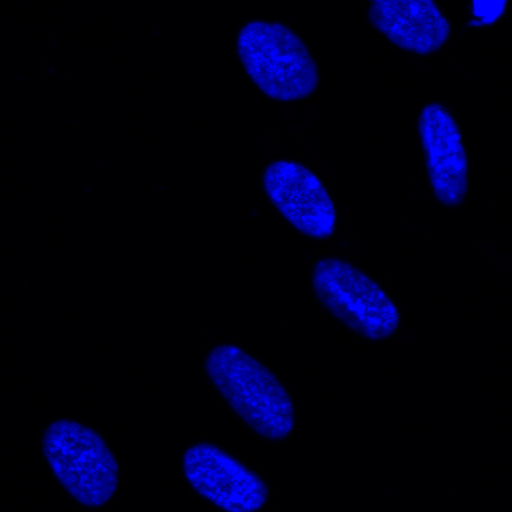

Supplement: Supplementary file 4 — Source data Fig. 3 [file 44318_2025_537_MOESM4_ESM.zip › EMBOJ-2025-120849-T_Source data Fig_3/Fig_3B/Images_Fig_3B/DAPI_UV_dose_10.tif]

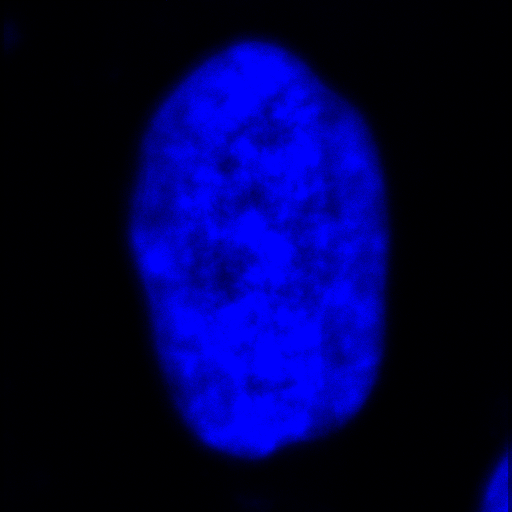

Supplement: Supplementary file 4 — Source data Fig. 3 [file 44318_2025_537_MOESM4_ESM.zip › EMBOJ-2025-120849-T_Source data Fig_3/Fig_3B/Images_Fig_3B/DAPI_UV_dose_20_magnifcation.tif]

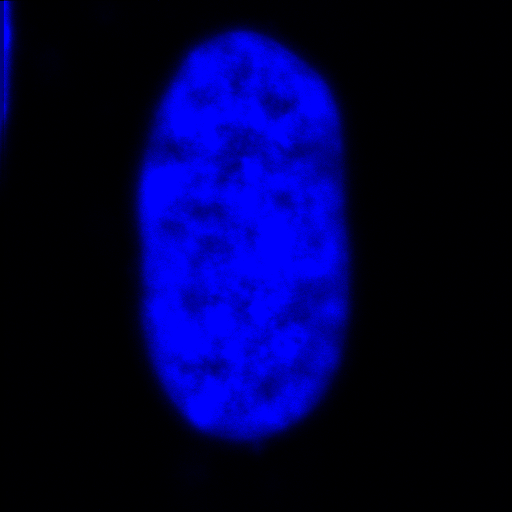

Supplement: Supplementary file 4 — Source data Fig. 3 [file 44318_2025_537_MOESM4_ESM.zip › EMBOJ-2025-120849-T_Source data Fig_3/Fig_3B/Images_Fig_3B/DAPI_UV_dose_0_magnifcation.tif]

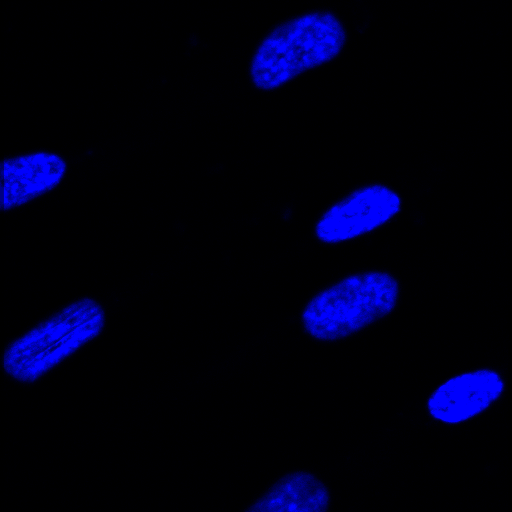

Supplement: Supplementary file 4 — Source data Fig. 3 [file 44318_2025_537_MOESM4_ESM.zip › EMBOJ-2025-120849-T_Source data Fig_3/Fig_3B/Images_Fig_3B/DAPI_UV_dose_0.tif]

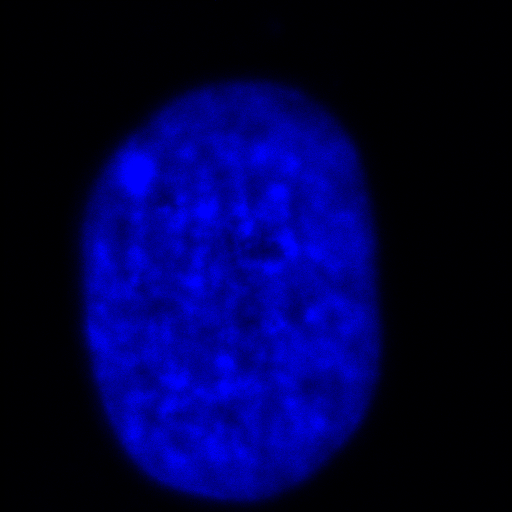

Supplement: Supplementary file 4 — Source data Fig. 3 [file 44318_2025_537_MOESM4_ESM.zip › EMBOJ-2025-120849-T_Source data Fig_3/Fig_3B/Images_Fig_3B/DAPI_UV_dose_5_magnifcation.tif]

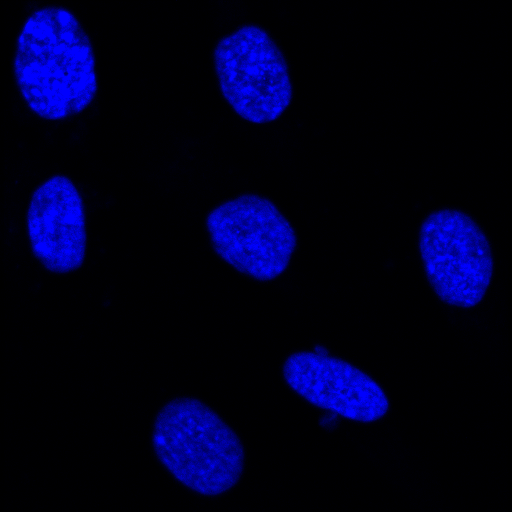

Supplement: Supplementary file 4 — Source data Fig. 3 [file 44318_2025_537_MOESM4_ESM.zip › EMBOJ-2025-120849-T_Source data Fig_3/Fig_3B/Images_Fig_3B/DAPI_UV_dose_5.tif]

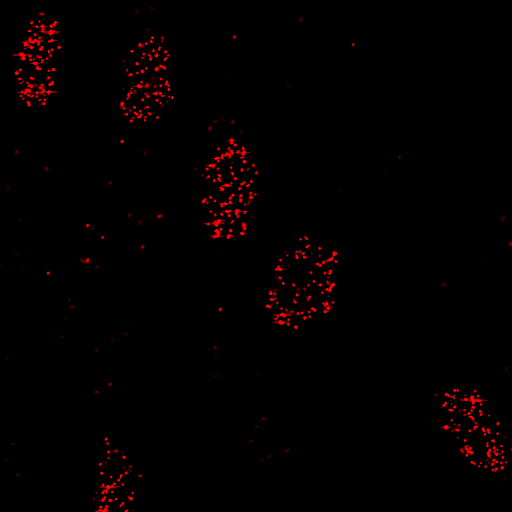

Supplement: Supplementary file 4 — Source data Fig. 3 [file 44318_2025_537_MOESM4_ESM.zip › EMBOJ-2025-120849-T_Source data Fig_3/Fig_3B/Images_Fig_3B/PLA_UV_dose_20.tif]

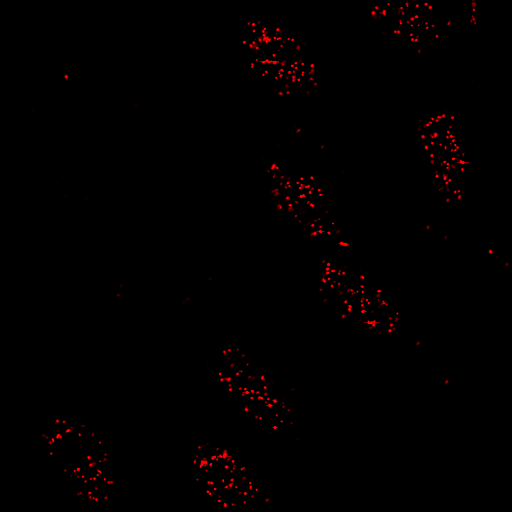

Supplement: Supplementary file 4 — Source data Fig. 3 [file 44318_2025_537_MOESM4_ESM.zip › EMBOJ-2025-120849-T_Source data Fig_3/Fig_3B/Images_Fig_3B/PLA_UV_dose_10.tif]

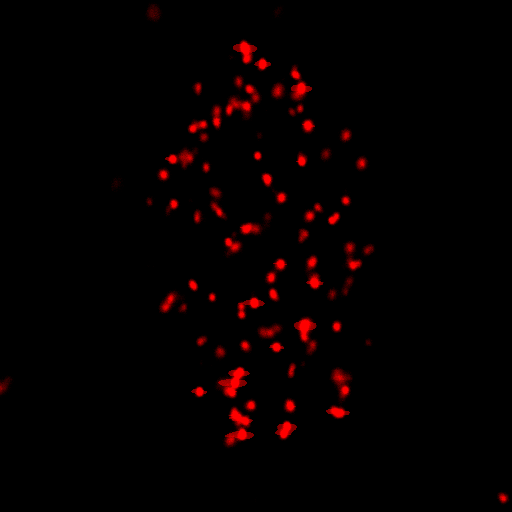

Supplement: Supplementary file 4 — Source data Fig. 3 [file 44318_2025_537_MOESM4_ESM.zip › EMBOJ-2025-120849-T_Source data Fig_3/Fig_3B/Images_Fig_3B/PLA_UV_dose_20_magnifcation.tif]

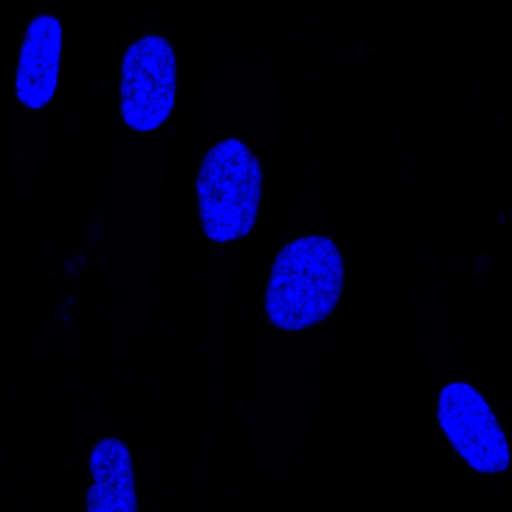

Supplement: Supplementary file 4 — Source data Fig. 3 [file 44318_2025_537_MOESM4_ESM.zip › EMBOJ-2025-120849-T_Source data Fig_3/Fig_3B/Images_Fig_3B/DAPI_UV_dose_20.tif]

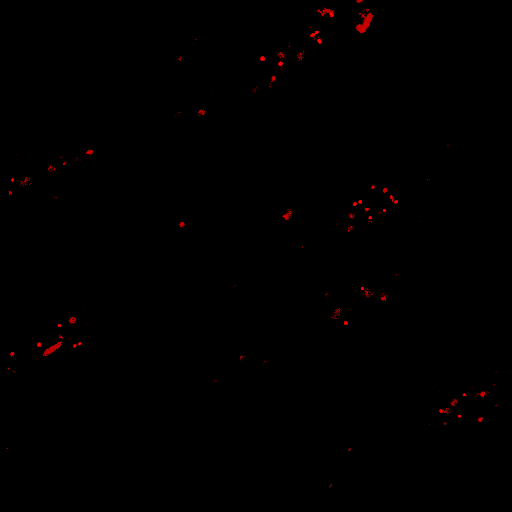

Supplement: Supplementary file 4 — Source data Fig. 3 [file 44318_2025_537_MOESM4_ESM.zip › EMBOJ-2025-120849-T_Source data Fig_3/Fig_3B/Images_Fig_3B/PLA_UV_dose_0.tif]

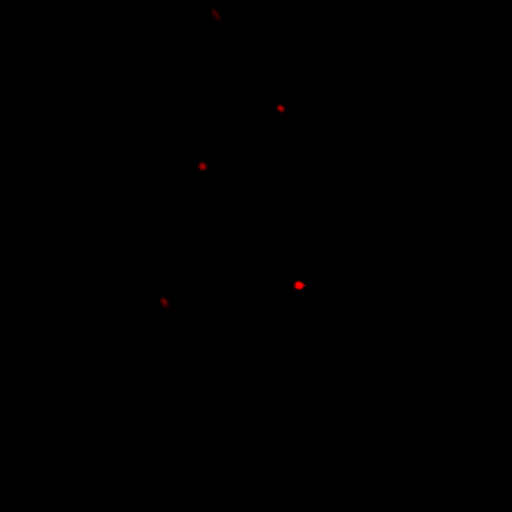

Supplement: Supplementary file 4 — Source data Fig. 3 [file 44318_2025_537_MOESM4_ESM.zip › EMBOJ-2025-120849-T_Source data Fig_3/Fig_3B/Images_Fig_3B/PLA_UV_dose_0_magnifcation.tif]

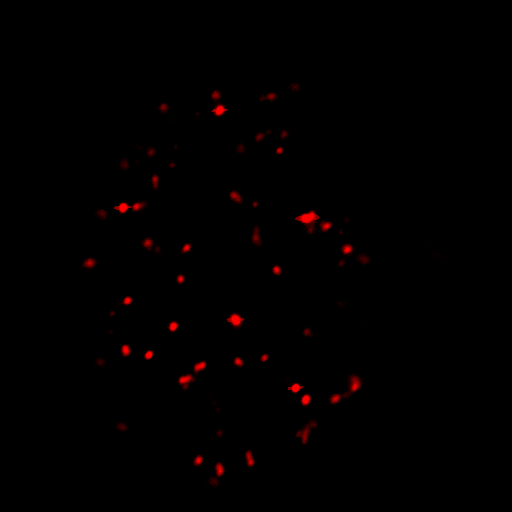

Supplement: Supplementary file 4 — Source data Fig. 3 [file 44318_2025_537_MOESM4_ESM.zip › EMBOJ-2025-120849-T_Source data Fig_3/Fig_3B/Images_Fig_3B/PLA_UV_dose_5_magnifcation.tif]

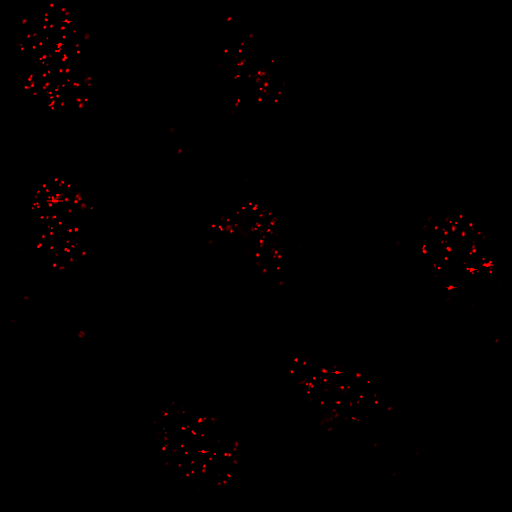

Supplement: Supplementary file 4 — Source data Fig. 3 [file 44318_2025_537_MOESM4_ESM.zip › EMBOJ-2025-120849-T_Source data Fig_3/Fig_3B/Images_Fig_3B/PLA_UV_dose_5.tif]

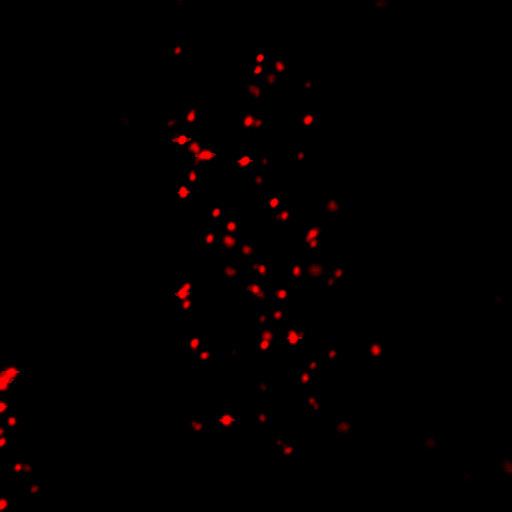

Supplement: Supplementary file 4 — Source data Fig. 3 [file 44318_2025_537_MOESM4_ESM.zip › EMBOJ-2025-120849-T_Source data Fig_3/Fig_3B/Images_Fig_3B/PLA_UV_dose_10_magnifcation.tif]

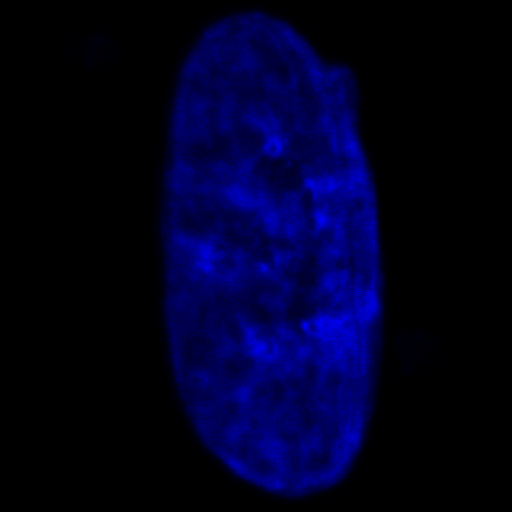

Supplement: Supplementary file 4 — Source data Fig. 3 [file 44318_2025_537_MOESM4_ESM.zip › EMBOJ-2025-120849-T_Source data Fig_3/Fig_3C/Fig_3C_GCN5/DAPI.tif]

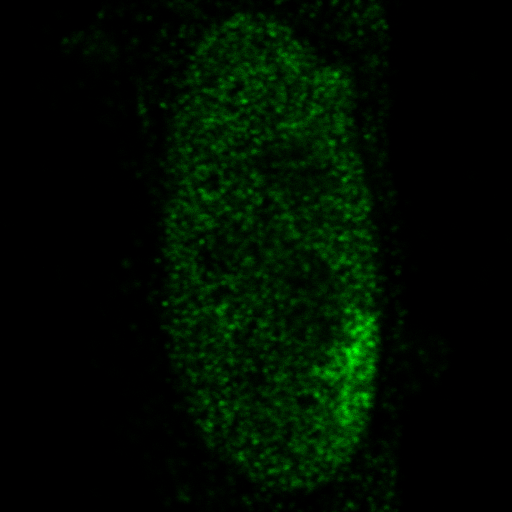

Supplement: Supplementary file 4 — Source data Fig. 3 [file 44318_2025_537_MOESM4_ESM.zip › EMBOJ-2025-120849-T_Source data Fig_3/Fig_3C/Fig_3C_GCN5/GCN5.tif]

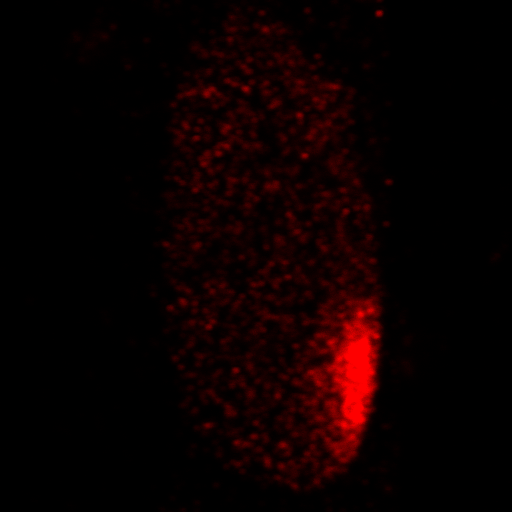

Supplement: Supplementary file 4 — Source data Fig. 3 [file 44318_2025_537_MOESM4_ESM.zip › EMBOJ-2025-120849-T_Source data Fig_3/Fig_3C/Fig_3C_GCN5/CPD.tif]

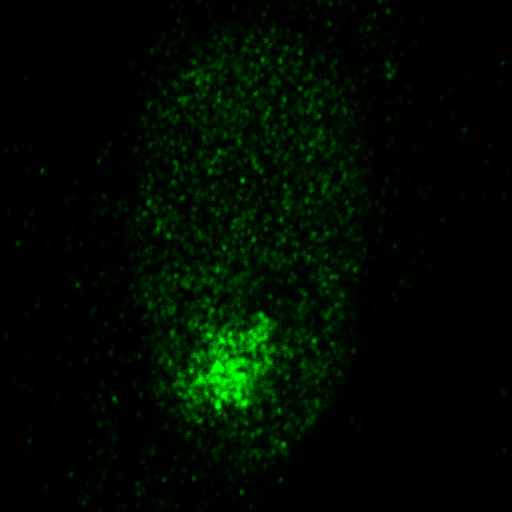

Supplement: Supplementary file 4 — Source data Fig. 3 [file 44318_2025_537_MOESM4_ESM.zip › EMBOJ-2025-120849-T_Source data Fig_3/Fig_3C/Fig_3C_PCAF/PCAF.tif]

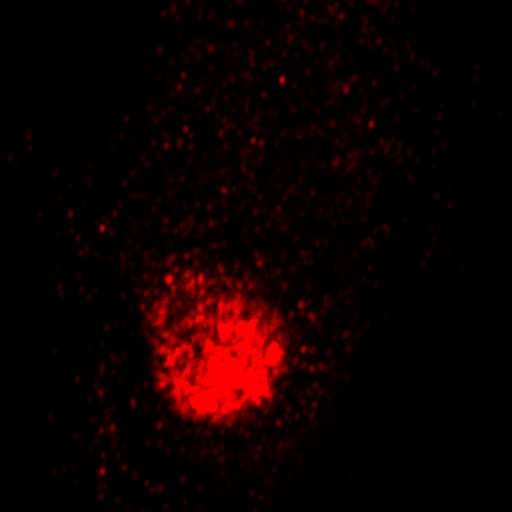

Supplement: Supplementary file 4 — Source data Fig. 3 [file 44318_2025_537_MOESM4_ESM.zip › EMBOJ-2025-120849-T_Source data Fig_3/Fig_3C/Fig_3C_PCAF/CPD.tif]

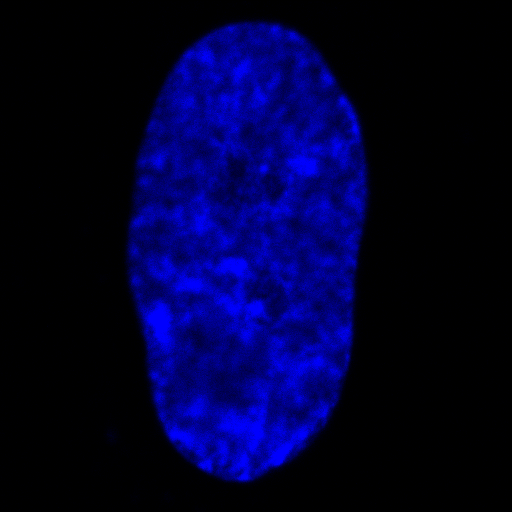

Supplement: Supplementary file 4 — Source data Fig. 3 [file 44318_2025_537_MOESM4_ESM.zip › EMBOJ-2025-120849-T_Source data Fig_3/Fig_3C/Fig_3C_PCAF/DAPI.tif]

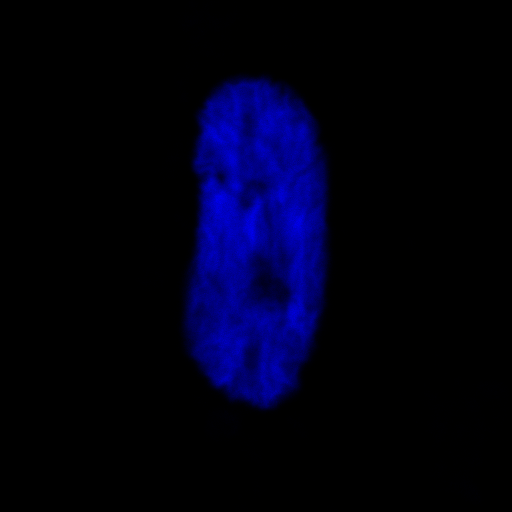

Supplement: Supplementary file 4 — Source data Fig. 3 [file 44318_2025_537_MOESM4_ESM.zip › EMBOJ-2025-120849-T_Source data Fig_3/Fig_3C/Fig_3C_p300/dapi.tif]

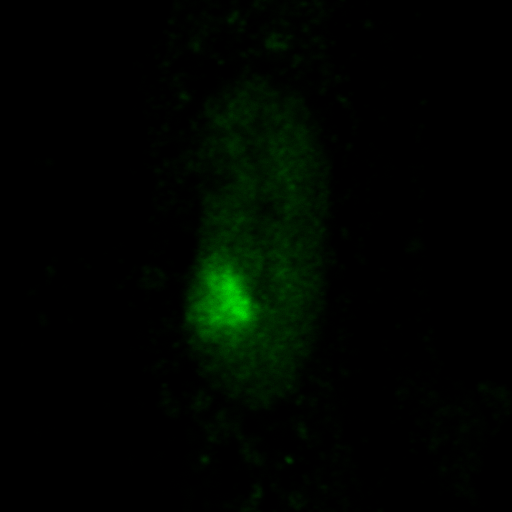

Supplement: Supplementary file 4 — Source data Fig. 3 [file 44318_2025_537_MOESM4_ESM.zip › EMBOJ-2025-120849-T_Source data Fig_3/Fig_3C/Fig_3C_p300/XPC.tif]

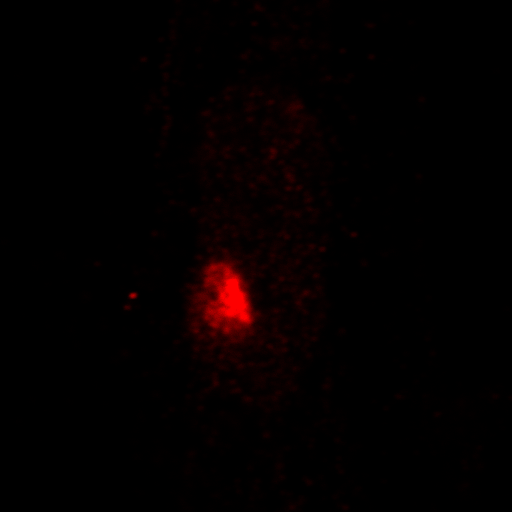

Supplement: Supplementary file 4 — Source data Fig. 3 [file 44318_2025_537_MOESM4_ESM.zip › EMBOJ-2025-120849-T_Source data Fig_3/Fig_3C/Fig_3C_p300/p300.tif]

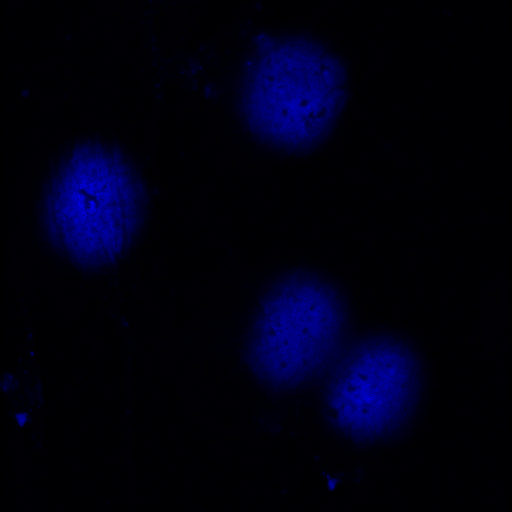

Supplement: Supplementary file 4 — Source data Fig. 3 [file 44318_2025_537_MOESM4_ESM.zip › EMBOJ-2025-120849-T_Source data Fig_3/Fig_3D/Images_Fig_3D/PLA_GCN5_and_S9.6/DAPI_untreated_1.tif]

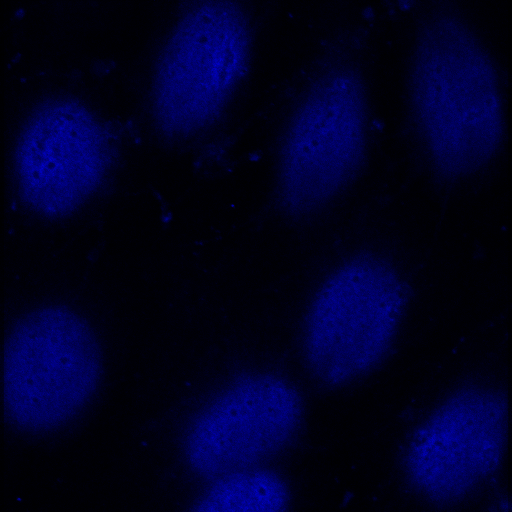

Supplement: Supplementary file 4 — Source data Fig. 3 [file 44318_2025_537_MOESM4_ESM.zip › EMBOJ-2025-120849-T_Source data Fig_3/Fig_3D/Images_Fig_3D/PLA_GCN5_and_S9.6/DAPI_UV.tif]

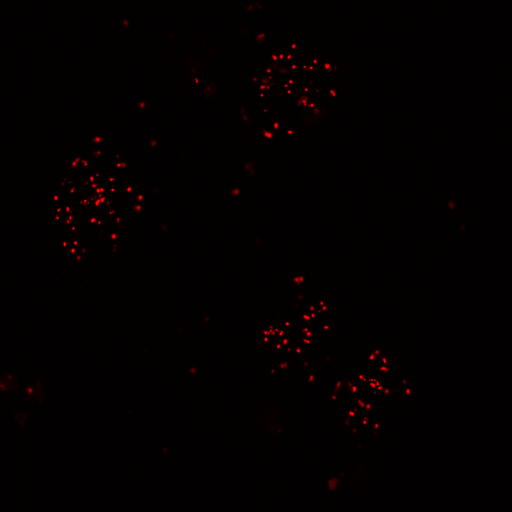

Supplement: Supplementary file 4 — Source data Fig. 3 [file 44318_2025_537_MOESM4_ESM.zip › EMBOJ-2025-120849-T_Source data Fig_3/Fig_3D/Images_Fig_3D/PLA_GCN5_and_S9.6/PLA_untreated_1.tif]

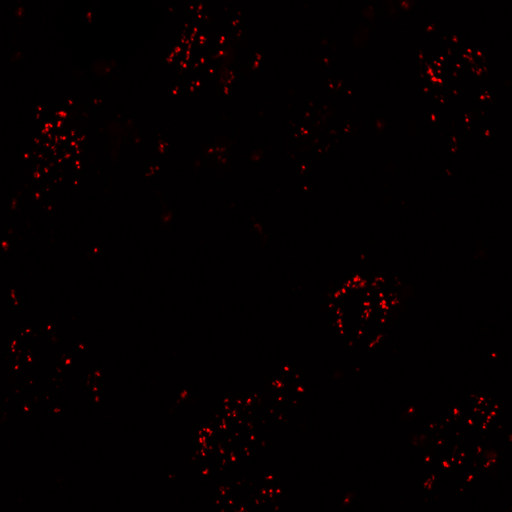

Supplement: Supplementary file 4 — Source data Fig. 3 [file 44318_2025_537_MOESM4_ESM.zip › EMBOJ-2025-120849-T_Source data Fig_3/Fig_3D/Images_Fig_3D/PLA_GCN5_and_S9.6/PLA_UV.tif]

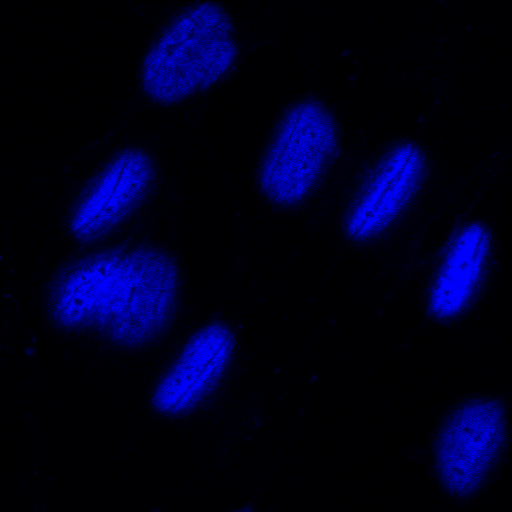

Supplement: Supplementary file 4 — Source data Fig. 3 [file 44318_2025_537_MOESM4_ESM.zip › EMBOJ-2025-120849-T_Source data Fig_3/Fig_3D/Images_Fig_3D/PLA_p300_and_S9.6/p300_untreated_DAPI.tif]

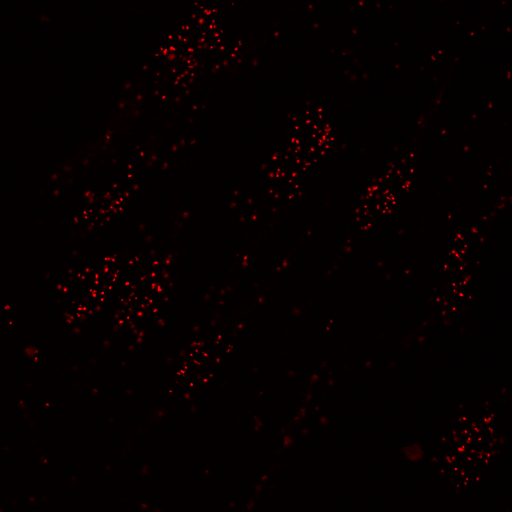

Supplement: Supplementary file 4 — Source data Fig. 3 [file 44318_2025_537_MOESM4_ESM.zip › EMBOJ-2025-120849-T_Source data Fig_3/Fig_3D/Images_Fig_3D/PLA_p300_and_S9.6/p300_untreated_PLA.tif]

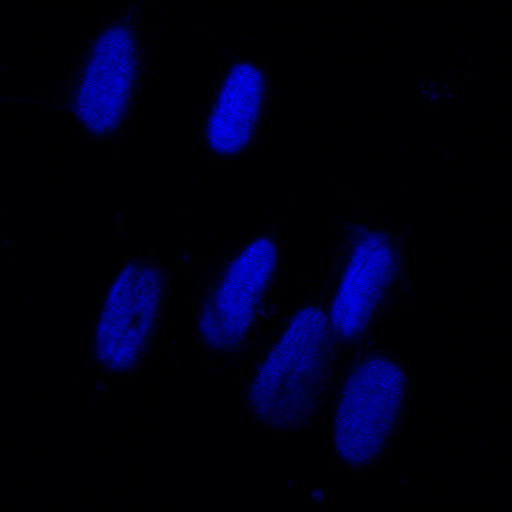

Supplement: Supplementary file 4 — Source data Fig. 3 [file 44318_2025_537_MOESM4_ESM.zip › EMBOJ-2025-120849-T_Source data Fig_3/Fig_3D/Images_Fig_3D/PLA_p300_and_S9.6/p300_UV_DAPI.tif]

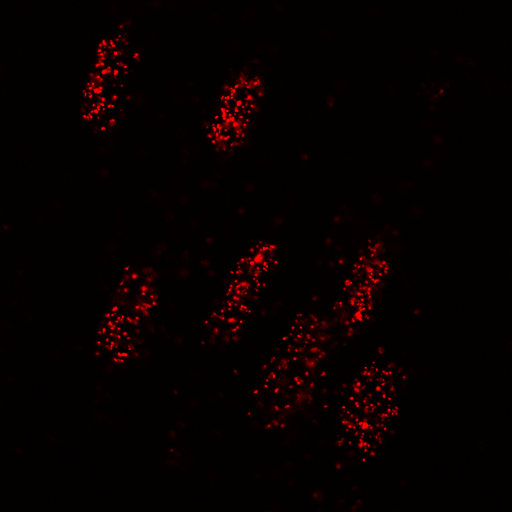

Supplement: Supplementary file 4 — Source data Fig. 3 [file 44318_2025_537_MOESM4_ESM.zip › EMBOJ-2025-120849-T_Source data Fig_3/Fig_3D/Images_Fig_3D/PLA_p300_and_S9.6/p300_UV_PLA.tif]

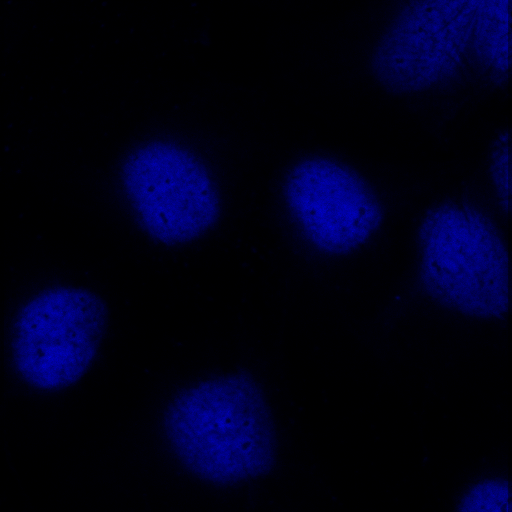

Supplement: Supplementary file 4 — Source data Fig. 3 [file 44318_2025_537_MOESM4_ESM.zip › EMBOJ-2025-120849-T_Source data Fig_3/Fig_3D/Images_Fig_3D/PLA_PCAF_and_S9.6/PLA_PCAF_untrearted_DAPI2f.tif]

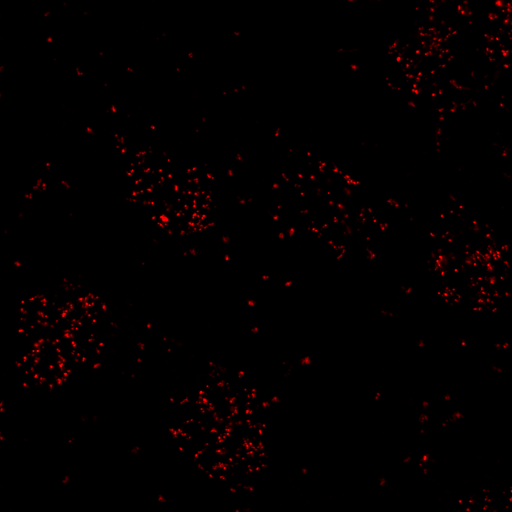

Supplement: Supplementary file 4 — Source data Fig. 3 [file 44318_2025_537_MOESM4_ESM.zip › EMBOJ-2025-120849-T_Source data Fig_3/Fig_3D/Images_Fig_3D/PLA_PCAF_and_S9.6/PLA_PCAF_UNTREATED_PLA2.tif]

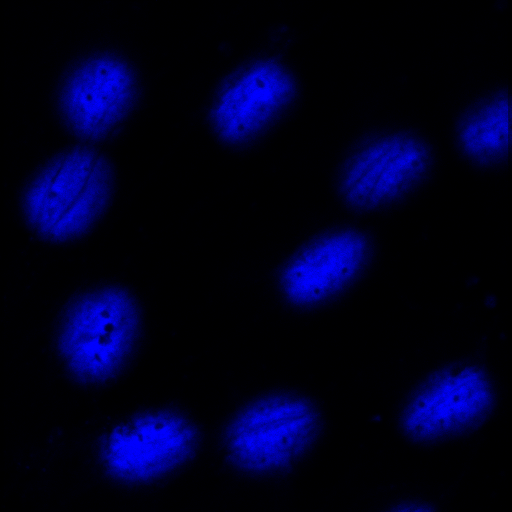

Supplement: Supplementary file 4 — Source data Fig. 3 [file 44318_2025_537_MOESM4_ESM.zip › EMBOJ-2025-120849-T_Source data Fig_3/Fig_3D/Images_Fig_3D/PLA_PCAF_and_S9.6/PLA_PCAF_UV_dapi2.tif]

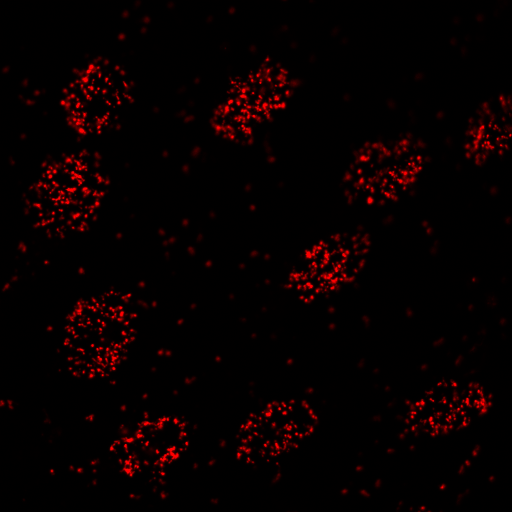

Supplement: Supplementary file 4 — Source data Fig. 3 [file 44318_2025_537_MOESM4_ESM.zip › EMBOJ-2025-120849-T_Source data Fig_3/Fig_3D/Images_Fig_3D/PLA_PCAF_and_S9.6/PLA_PCAF_UV_PLA2.tif]

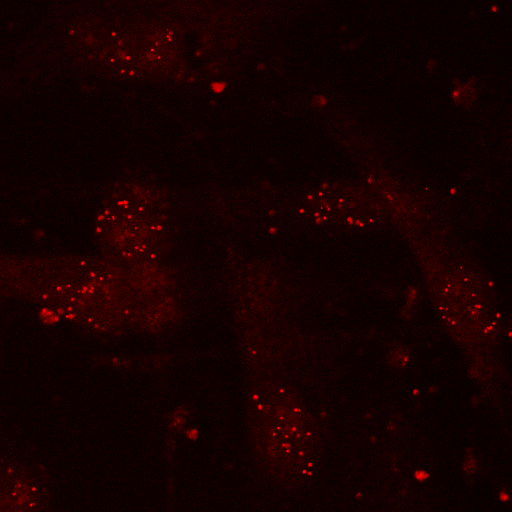

Supplement: Supplementary file 5 — Source data Fig. 4 [file 44318_2025_537_MOESM5_ESM.zip › EMBOJ-2025-120849-T_Source data Fig_4/Fig_4A/Images_Fig_4A_PLA_GCN5_CPD/PLA_THZ1+UV.tif]

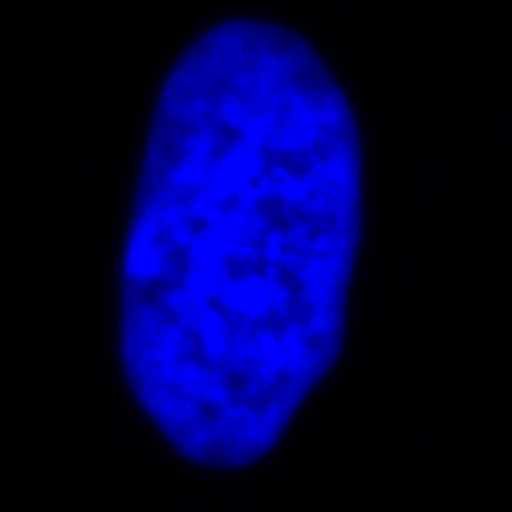

Supplement: Supplementary file 5 — Source data Fig. 4 [file 44318_2025_537_MOESM5_ESM.zip › EMBOJ-2025-120849-T_Source data Fig_4/Fig_4A/Images_Fig_4A_PLA_GCN5_CPD/DAPI_THZ1+UV_magnification.tif]

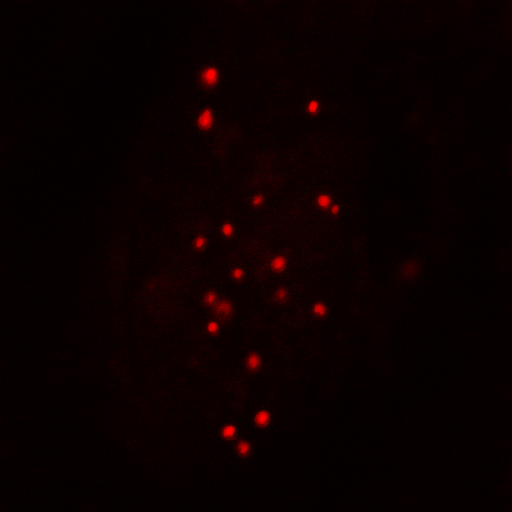

Supplement: Supplementary file 5 — Source data Fig. 4 [file 44318_2025_537_MOESM5_ESM.zip › EMBOJ-2025-120849-T_Source data Fig_4/Fig_4A/Images_Fig_4A_PLA_GCN5_CPD/PLA_THZ1+UV_magnification.tif]

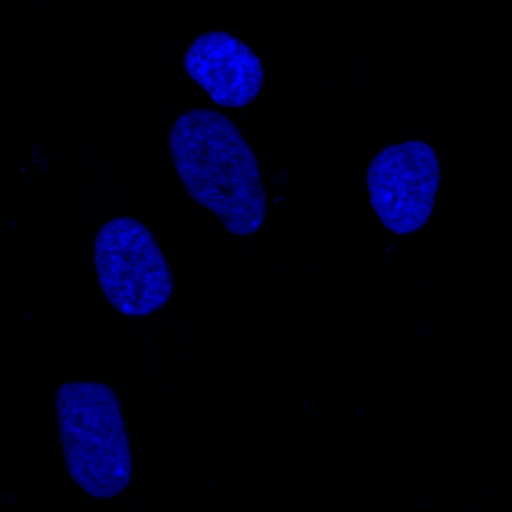

Supplement: Supplementary file 5 — Source data Fig. 4 [file 44318_2025_537_MOESM5_ESM.zip › EMBOJ-2025-120849-T_Source data Fig_4/Fig_4A/Images_Fig_4A_PLA_GCN5_CPD/DAPI_untreated.tif]

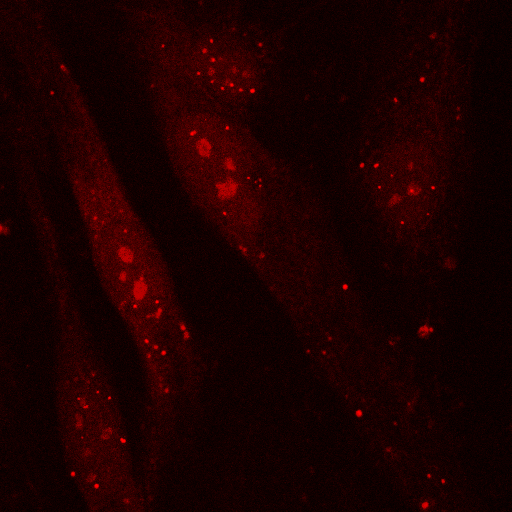

Supplement: Supplementary file 5 — Source data Fig. 4 [file 44318_2025_537_MOESM5_ESM.zip › EMBOJ-2025-120849-T_Source data Fig_4/Fig_4A/Images_Fig_4A_PLA_GCN5_CPD/PLA_untreated.tif]

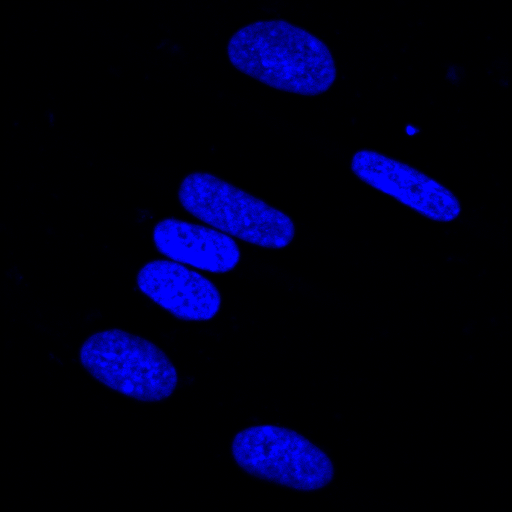

Supplement: Supplementary file 5 — Source data Fig. 4 [file 44318_2025_537_MOESM5_ESM.zip › EMBOJ-2025-120849-T_Source data Fig_4/Fig_4A/Images_Fig_4A_PLA_GCN5_CPD/DAPI_UV.tif]

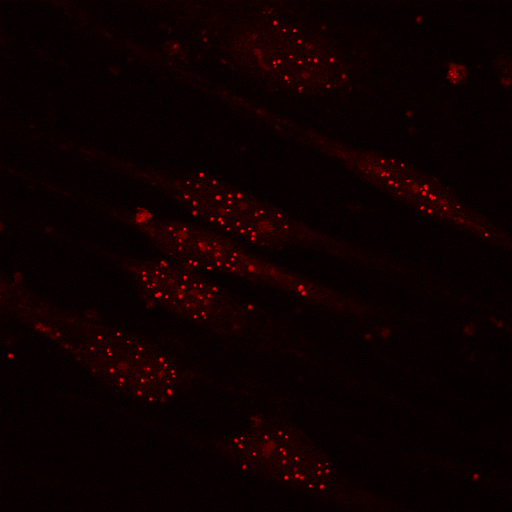

Supplement: Supplementary file 5 — Source data Fig. 4 [file 44318_2025_537_MOESM5_ESM.zip › EMBOJ-2025-120849-T_Source data Fig_4/Fig_4A/Images_Fig_4A_PLA_GCN5_CPD/PLA_UV.tif]

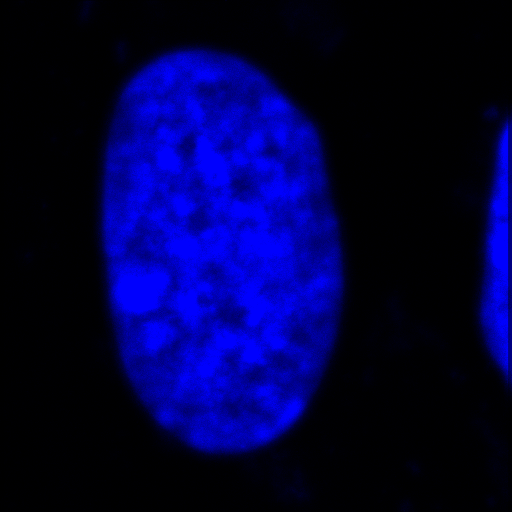

Supplement: Supplementary file 5 — Source data Fig. 4 [file 44318_2025_537_MOESM5_ESM.zip › EMBOJ-2025-120849-T_Source data Fig_4/Fig_4A/Images_Fig_4A_PLA_GCN5_CPD/DAPI_UV_magnification.tif]

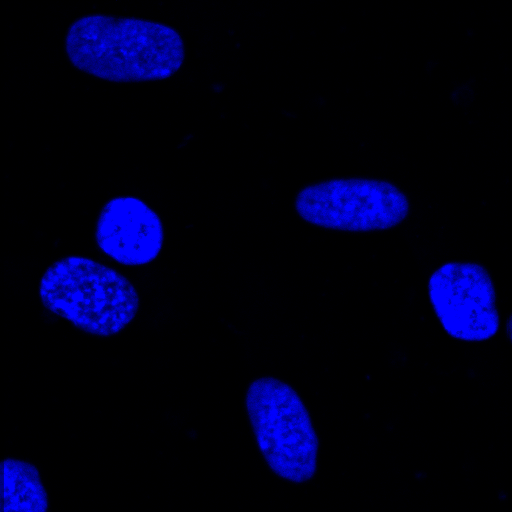

Supplement: Supplementary file 5 — Source data Fig. 4 [file 44318_2025_537_MOESM5_ESM.zip › EMBOJ-2025-120849-T_Source data Fig_4/Fig_4A/Images_Fig_4A_PLA_GCN5_CPD/DAPI_THZ1+UV.tif]

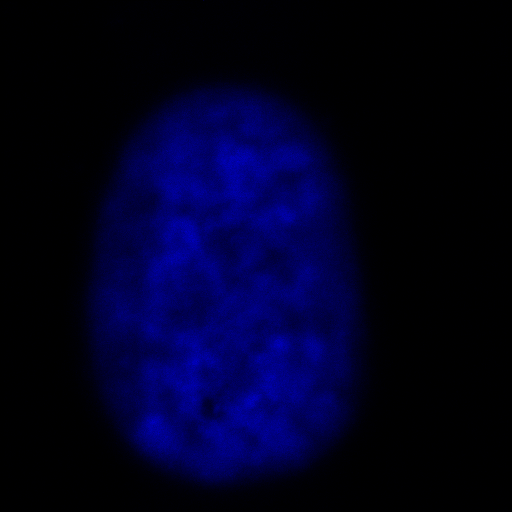

Supplement: Supplementary file 5 — Source data Fig. 4 [file 44318_2025_537_MOESM5_ESM.zip › EMBOJ-2025-120849-T_Source data Fig_4/Fig_4A/Images_Fig_4A_PLA_GCN5_CPD/DAPI_untreated_magnification.tif]

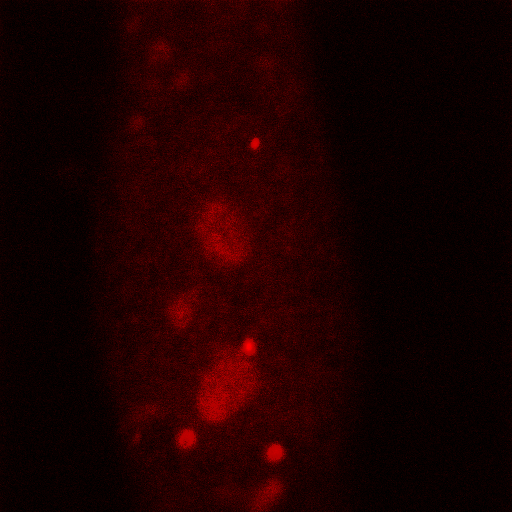

Supplement: Supplementary file 5 — Source data Fig. 4 [file 44318_2025_537_MOESM5_ESM.zip › EMBOJ-2025-120849-T_Source data Fig_4/Fig_4A/Images_Fig_4A_PLA_GCN5_CPD/PLA_untreated_magnification.tif]

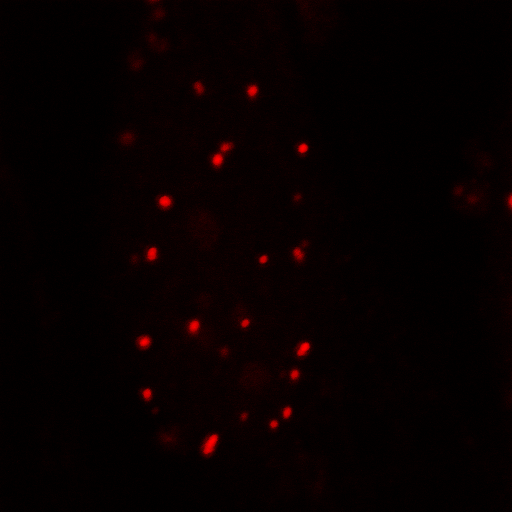

Supplement: Supplementary file 5 — Source data Fig. 4 [file 44318_2025_537_MOESM5_ESM.zip › EMBOJ-2025-120849-T_Source data Fig_4/Fig_4A/Images_Fig_4A_PLA_GCN5_CPD/PLA_UV_magnification.tif]

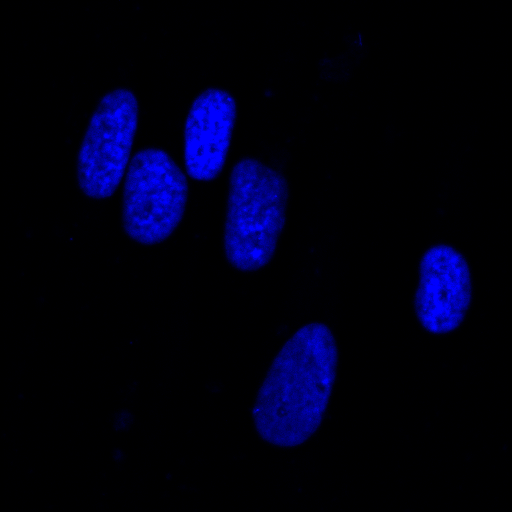

Supplement: Supplementary file 5 — Source data Fig. 4 [file 44318_2025_537_MOESM5_ESM.zip › EMBOJ-2025-120849-T_Source data Fig_4/Fig_4A/Images_Fig_4A_PLA_PCAF_CPD/DAPI_UV.tif]

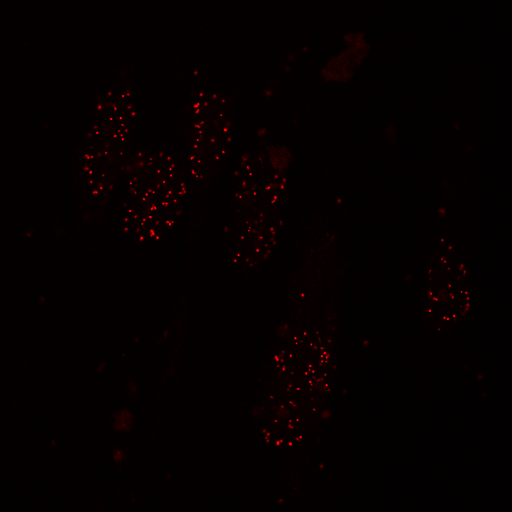

Supplement: Supplementary file 5 — Source data Fig. 4 [file 44318_2025_537_MOESM5_ESM.zip › EMBOJ-2025-120849-T_Source data Fig_4/Fig_4A/Images_Fig_4A_PLA_PCAF_CPD/PLA_UV.tif]

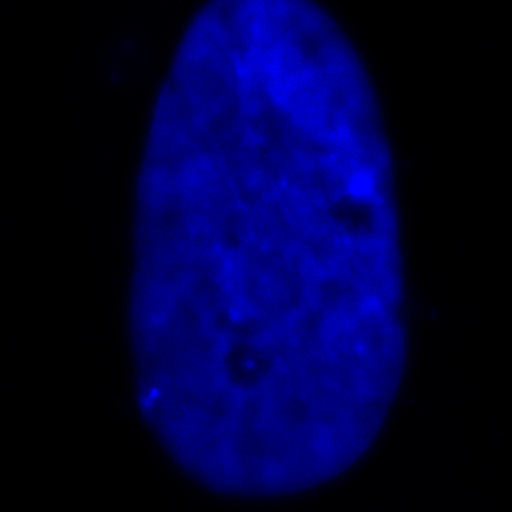

Supplement: Supplementary file 5 — Source data Fig. 4 [file 44318_2025_537_MOESM5_ESM.zip › EMBOJ-2025-120849-T_Source data Fig_4/Fig_4A/Images_Fig_4A_PLA_PCAF_CPD/DAPI_UV_magnification.tif]

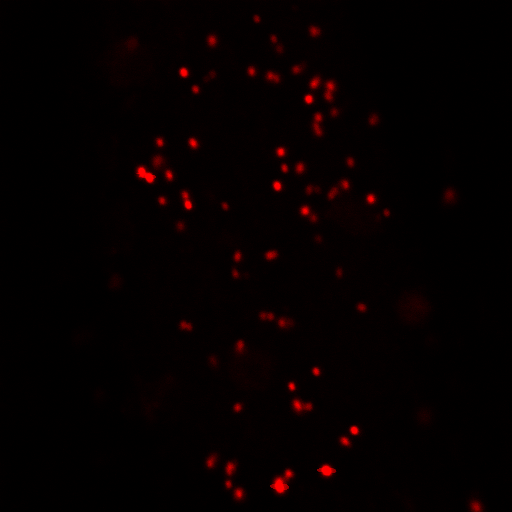

Supplement: Supplementary file 5 — Source data Fig. 4 [file 44318_2025_537_MOESM5_ESM.zip › EMBOJ-2025-120849-T_Source data Fig_4/Fig_4A/Images_Fig_4A_PLA_PCAF_CPD/PLA_UV_magnification.tif]

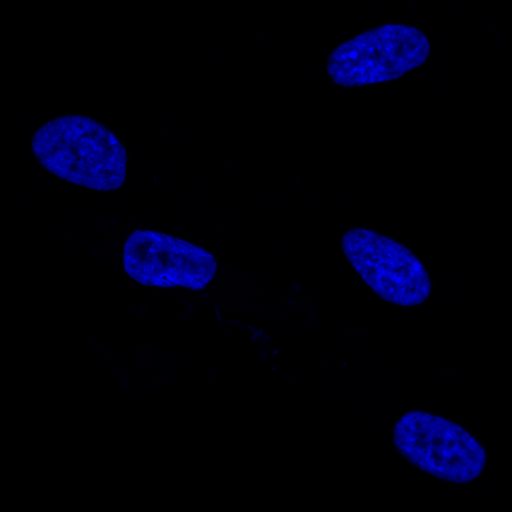

Supplement: Supplementary file 5 — Source data Fig. 4 [file 44318_2025_537_MOESM5_ESM.zip › EMBOJ-2025-120849-T_Source data Fig_4/Fig_4A/Images_Fig_4A_PLA_PCAF_CPD/DAPI_untreated.tif]

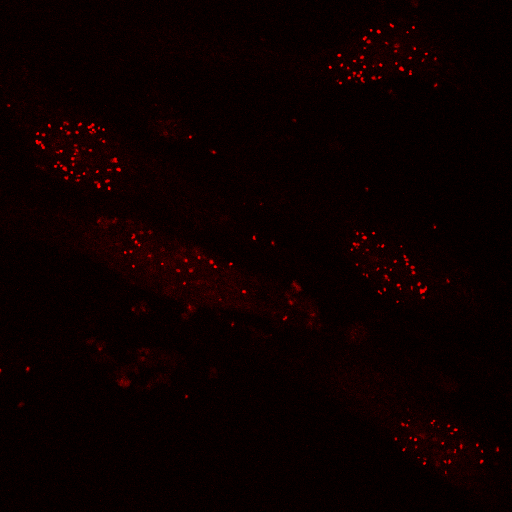

Supplement: Supplementary file 5 — Source data Fig. 4 [file 44318_2025_537_MOESM5_ESM.zip › EMBOJ-2025-120849-T_Source data Fig_4/Fig_4A/Images_Fig_4A_PLA_PCAF_CPD/PLA_untreated.tif]

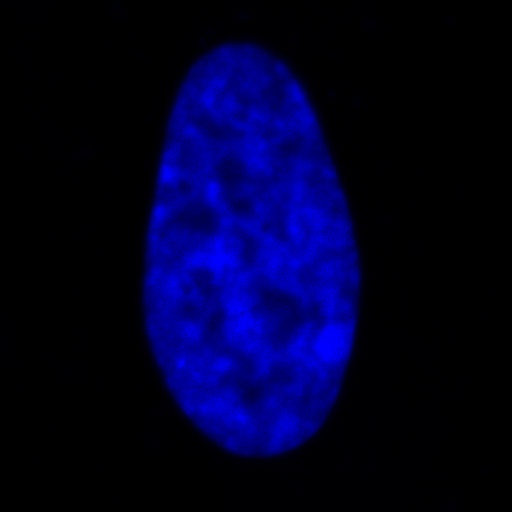

Supplement: Supplementary file 5 — Source data Fig. 4 [file 44318_2025_537_MOESM5_ESM.zip › EMBOJ-2025-120849-T_Source data Fig_4/Fig_4A/Images_Fig_4A_PLA_PCAF_CPD/DAPI_THZ1_UV_magnification.tif]

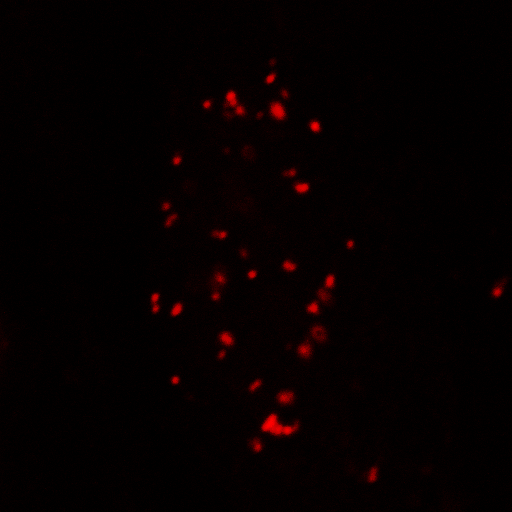

Supplement: Supplementary file 5 — Source data Fig. 4 [file 44318_2025_537_MOESM5_ESM.zip › EMBOJ-2025-120849-T_Source data Fig_4/Fig_4A/Images_Fig_4A_PLA_PCAF_CPD/PLA_THZ1_UV_magnification.tif]

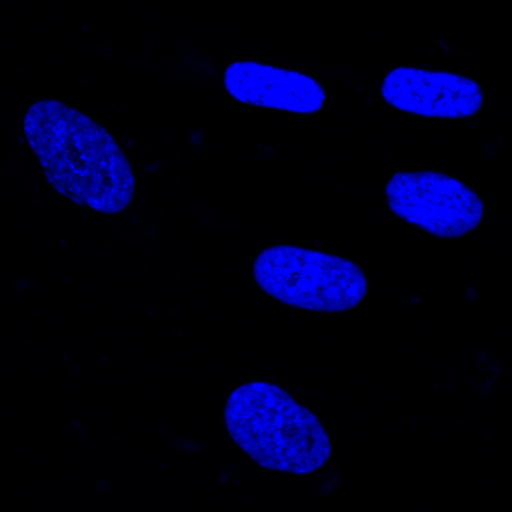

Supplement: Supplementary file 5 — Source data Fig. 4 [file 44318_2025_537_MOESM5_ESM.zip › EMBOJ-2025-120849-T_Source data Fig_4/Fig_4A/Images_Fig_4A_PLA_PCAF_CPD/DAPI_THZ1_UV.tif]

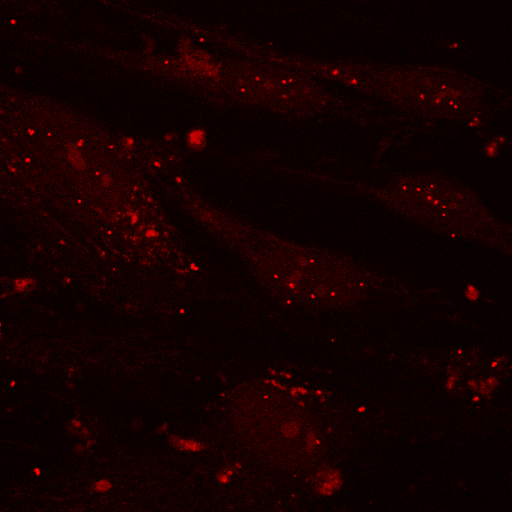

Supplement: Supplementary file 5 — Source data Fig. 4 [file 44318_2025_537_MOESM5_ESM.zip › EMBOJ-2025-120849-T_Source data Fig_4/Fig_4A/Images_Fig_4A_PLA_PCAF_CPD/PLA_untreated_magnification.tif]

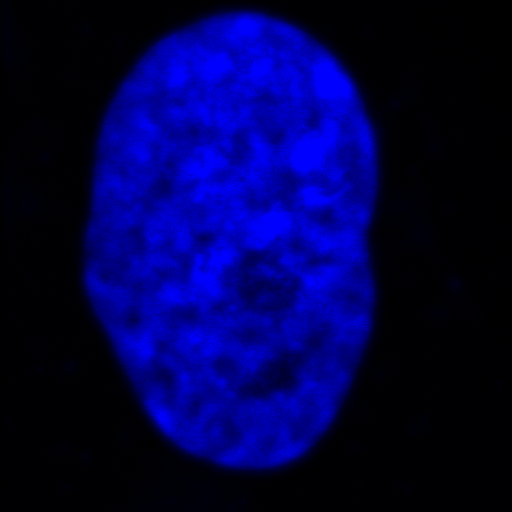

Supplement: Supplementary file 5 — Source data Fig. 4 [file 44318_2025_537_MOESM5_ESM.zip › EMBOJ-2025-120849-T_Source data Fig_4/Fig_4A/Images_Fig_4A_PLA_PCAF_CPD/DAPI_untreated_magnification.tif]

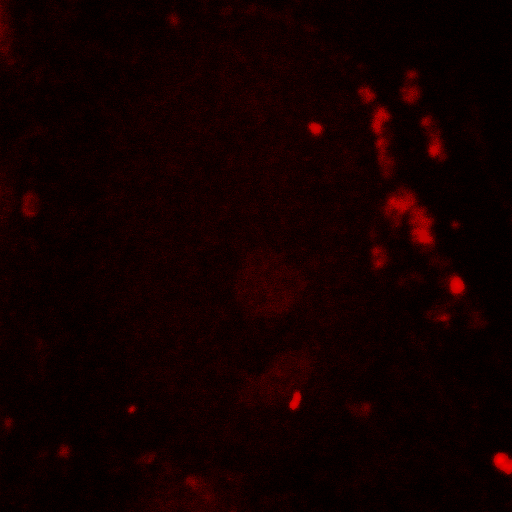

Supplement: Supplementary file 5 — Source data Fig. 4 [file 44318_2025_537_MOESM5_ESM.zip › EMBOJ-2025-120849-T_Source data Fig_4/Fig_4A/Images_Fig_4A_PLA_PCAF_CPD/PLA_THZ1_UV.tif]

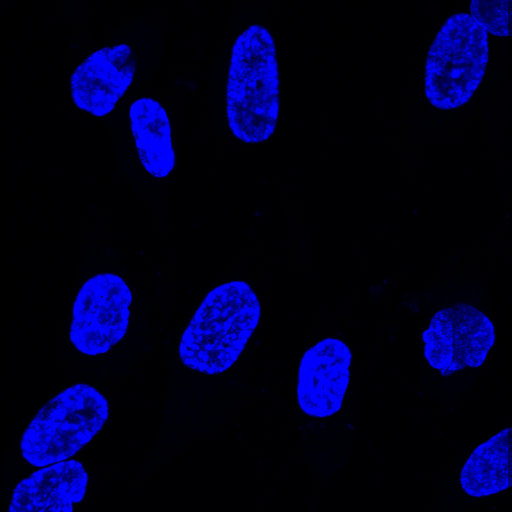

Supplement: Supplementary file 5 — Source data Fig. 4 [file 44318_2025_537_MOESM5_ESM.zip › EMBOJ-2025-120849-T_Source data Fig_4/Fig_4A/Images_Fig_4A_PLA_p300_CPD/DAPI_THZ1+UV.tif]

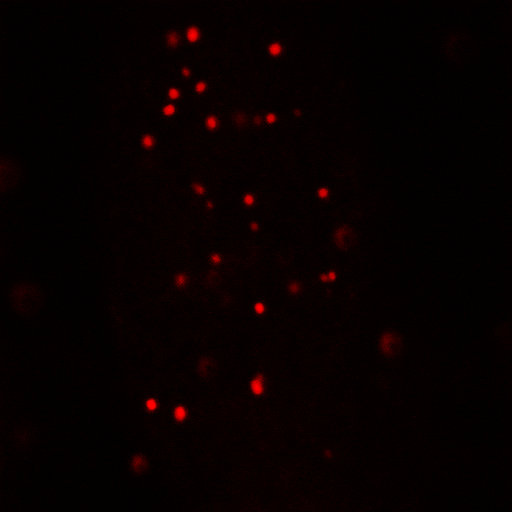

Supplement: Supplementary file 5 — Source data Fig. 4 [file 44318_2025_537_MOESM5_ESM.zip › EMBOJ-2025-120849-T_Source data Fig_4/Fig_4A/Images_Fig_4A_PLA_p300_CPD/PLA_THZ1+UV_magnification.tif]

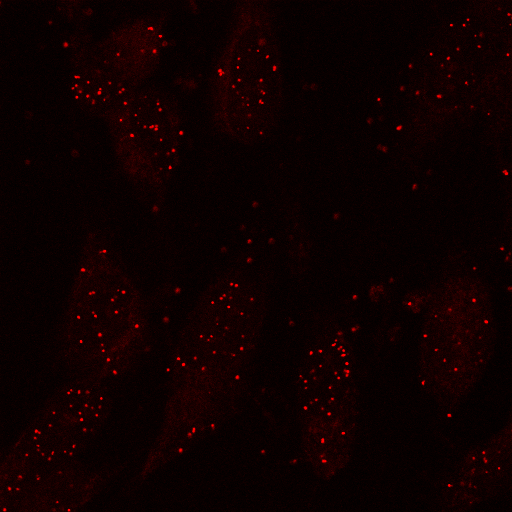

Supplement: Supplementary file 5 — Source data Fig. 4 [file 44318_2025_537_MOESM5_ESM.zip › EMBOJ-2025-120849-T_Source data Fig_4/Fig_4A/Images_Fig_4A_PLA_p300_CPD/PLA_THZ1+UV.tif]

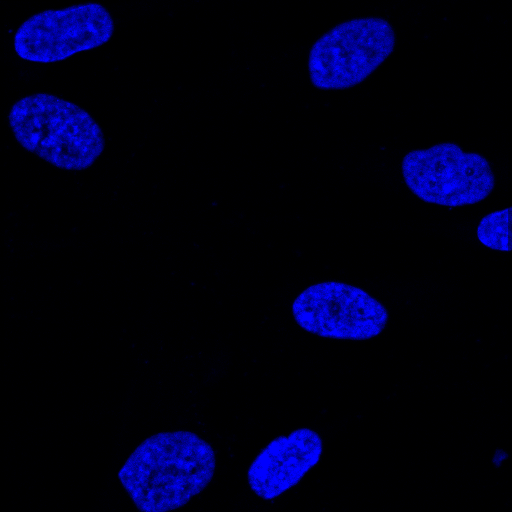

Supplement: Supplementary file 5 — Source data Fig. 4 [file 44318_2025_537_MOESM5_ESM.zip › EMBOJ-2025-120849-T_Source data Fig_4/Fig_4A/Images_Fig_4A_PLA_p300_CPD/DAPI_untreated.tif]

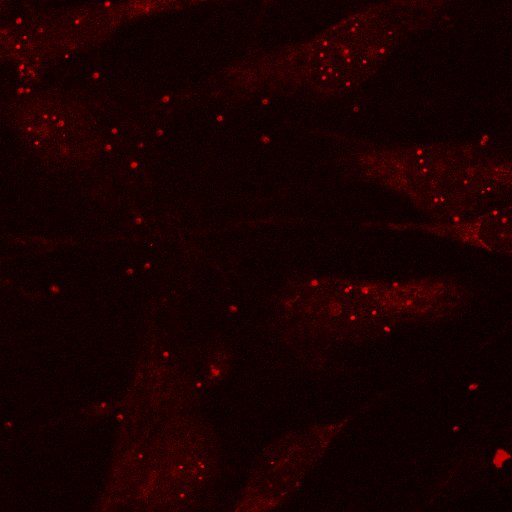

Supplement: Supplementary file 5 — Source data Fig. 4 [file 44318_2025_537_MOESM5_ESM.zip › EMBOJ-2025-120849-T_Source data Fig_4/Fig_4A/Images_Fig_4A_PLA_p300_CPD/PLA_untreated.tif]

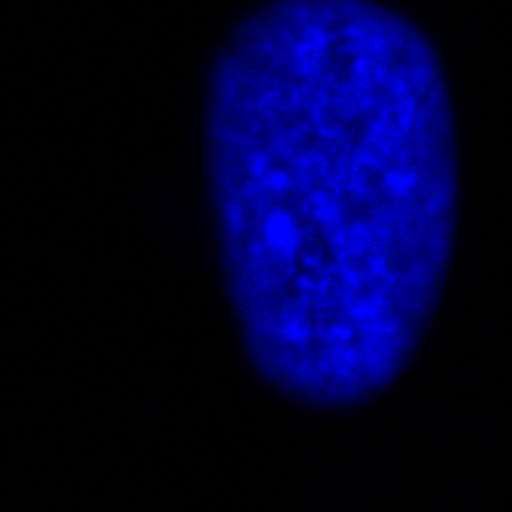

Supplement: Supplementary file 5 — Source data Fig. 4 [file 44318_2025_537_MOESM5_ESM.zip › EMBOJ-2025-120849-T_Source data Fig_4/Fig_4A/Images_Fig_4A_PLA_p300_CPD/DAPI_UV_magnification.tif]

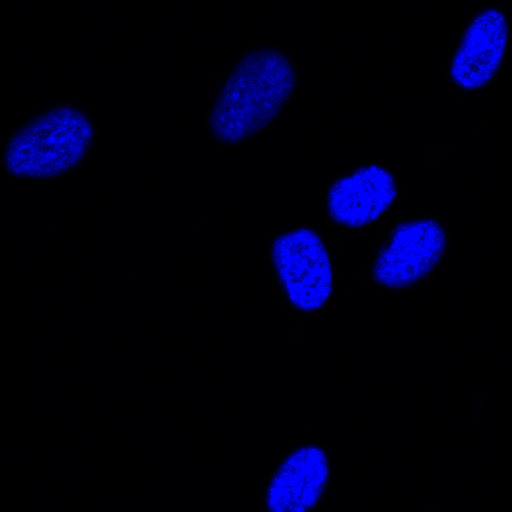

Supplement: Supplementary file 5 — Source data Fig. 4 [file 44318_2025_537_MOESM5_ESM.zip › EMBOJ-2025-120849-T_Source data Fig_4/Fig_4A/Images_Fig_4A_PLA_p300_CPD/DAPI_UV.tif]

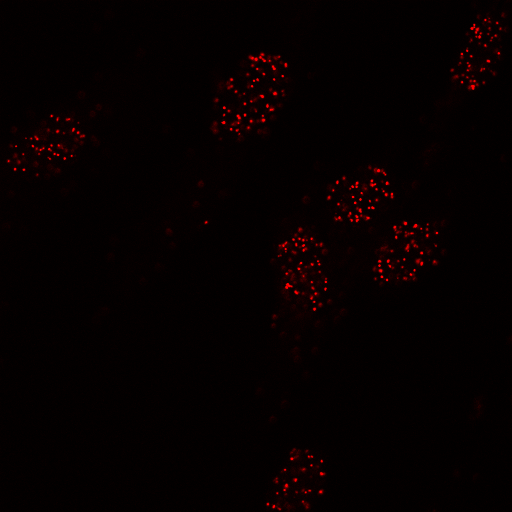

Supplement: Supplementary file 5 — Source data Fig. 4 [file 44318_2025_537_MOESM5_ESM.zip › EMBOJ-2025-120849-T_Source data Fig_4/Fig_4A/Images_Fig_4A_PLA_p300_CPD/PLA_UV.tif]

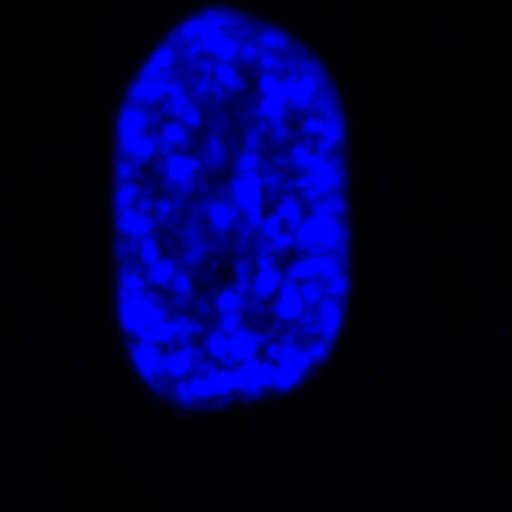

Supplement: Supplementary file 5 — Source data Fig. 4 [file 44318_2025_537_MOESM5_ESM.zip › EMBOJ-2025-120849-T_Source data Fig_4/Fig_4A/Images_Fig_4A_PLA_p300_CPD/DAPI_THZ1+UV_magnification.tif]

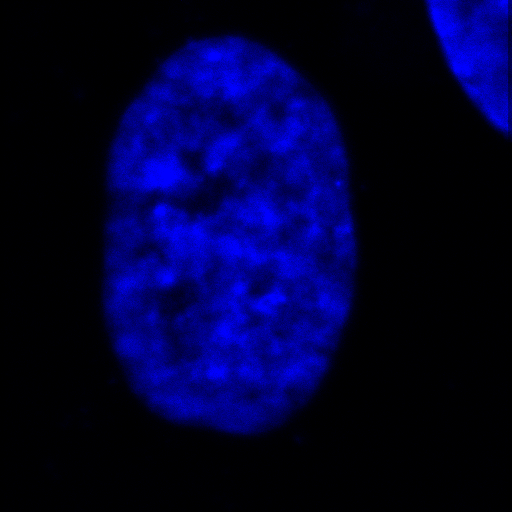

Supplement: Supplementary file 5 — Source data Fig. 4 [file 44318_2025_537_MOESM5_ESM.zip › EMBOJ-2025-120849-T_Source data Fig_4/Fig_4A/Images_Fig_4A_PLA_p300_CPD/DAPI_untreated_magnification.tif]

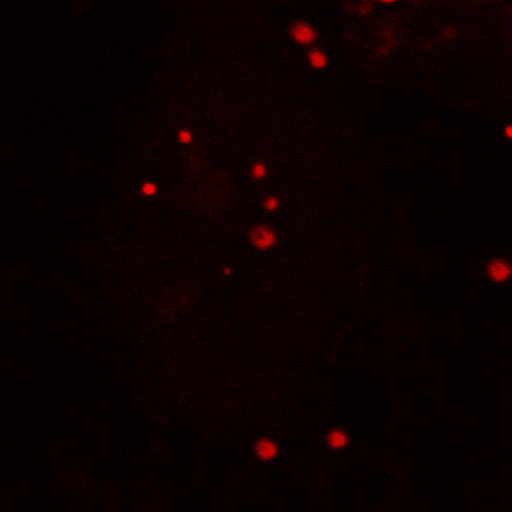

Supplement: Supplementary file 5 — Source data Fig. 4 [file 44318_2025_537_MOESM5_ESM.zip › EMBOJ-2025-120849-T_Source data Fig_4/Fig_4A/Images_Fig_4A_PLA_p300_CPD/PLA_untreated_magnification.tif]

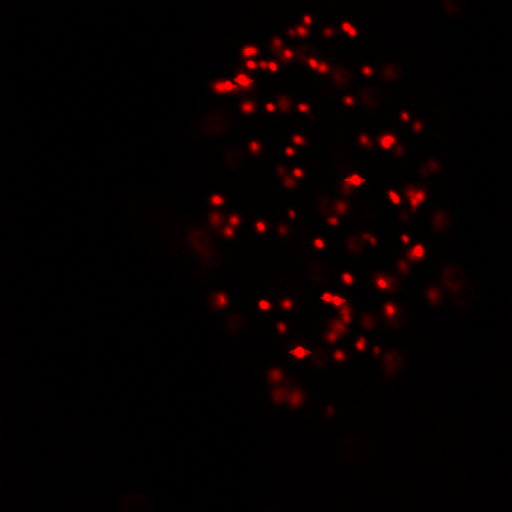

Supplement: Supplementary file 5 — Source data Fig. 4 [file 44318_2025_537_MOESM5_ESM.zip › EMBOJ-2025-120849-T_Source data Fig_4/Fig_4A/Images_Fig_4A_PLA_p300_CPD/PLA_UV_magnification.tif]

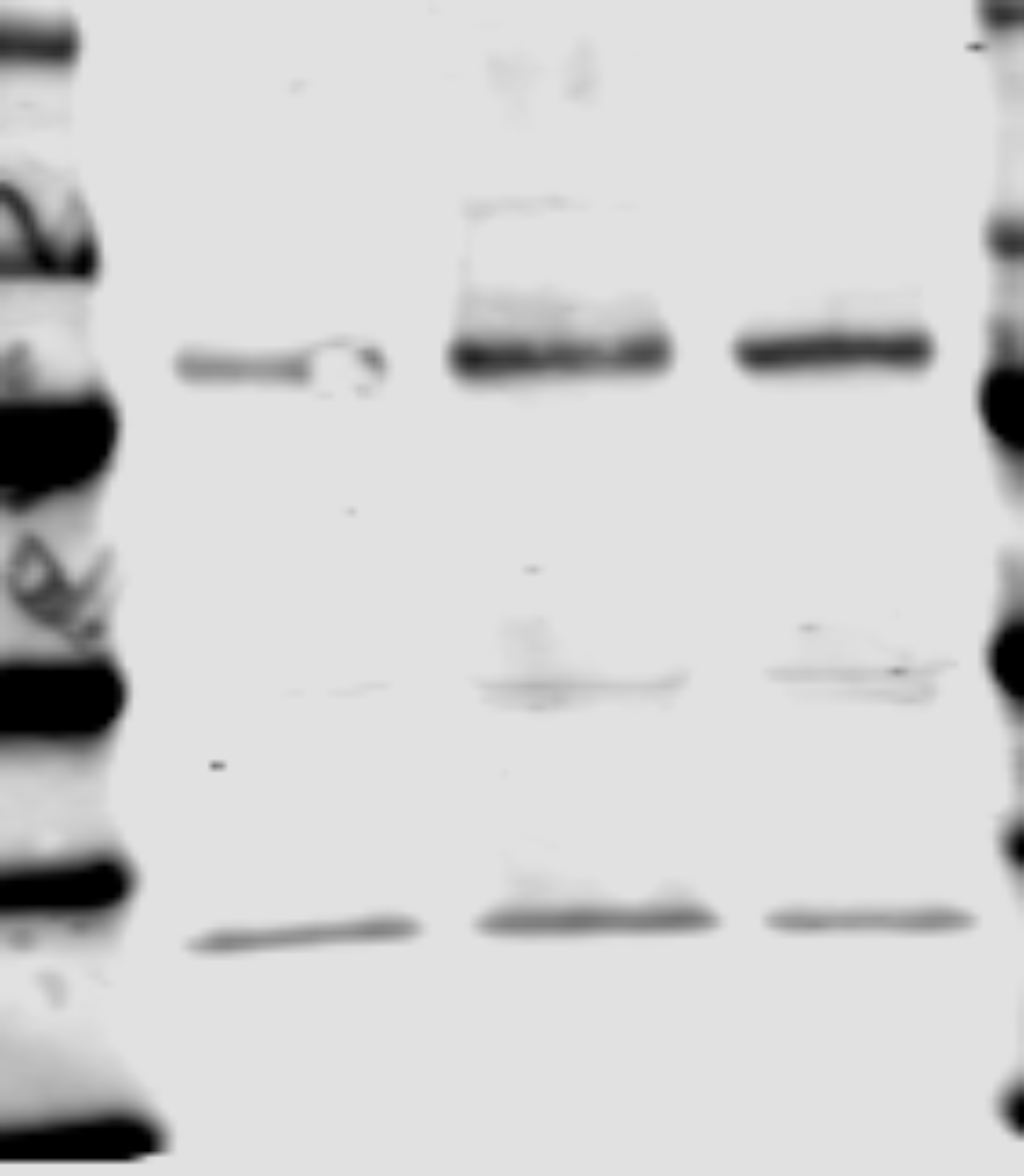

Supplement: Supplementary file 5 — Source data Fig. 4 [file 44318_2025_537_MOESM5_ESM.zip › EMBOJ-2025-120849-T_Source data Fig_4/Fig_4B/Images_Fig_4B/PCAF.tif]

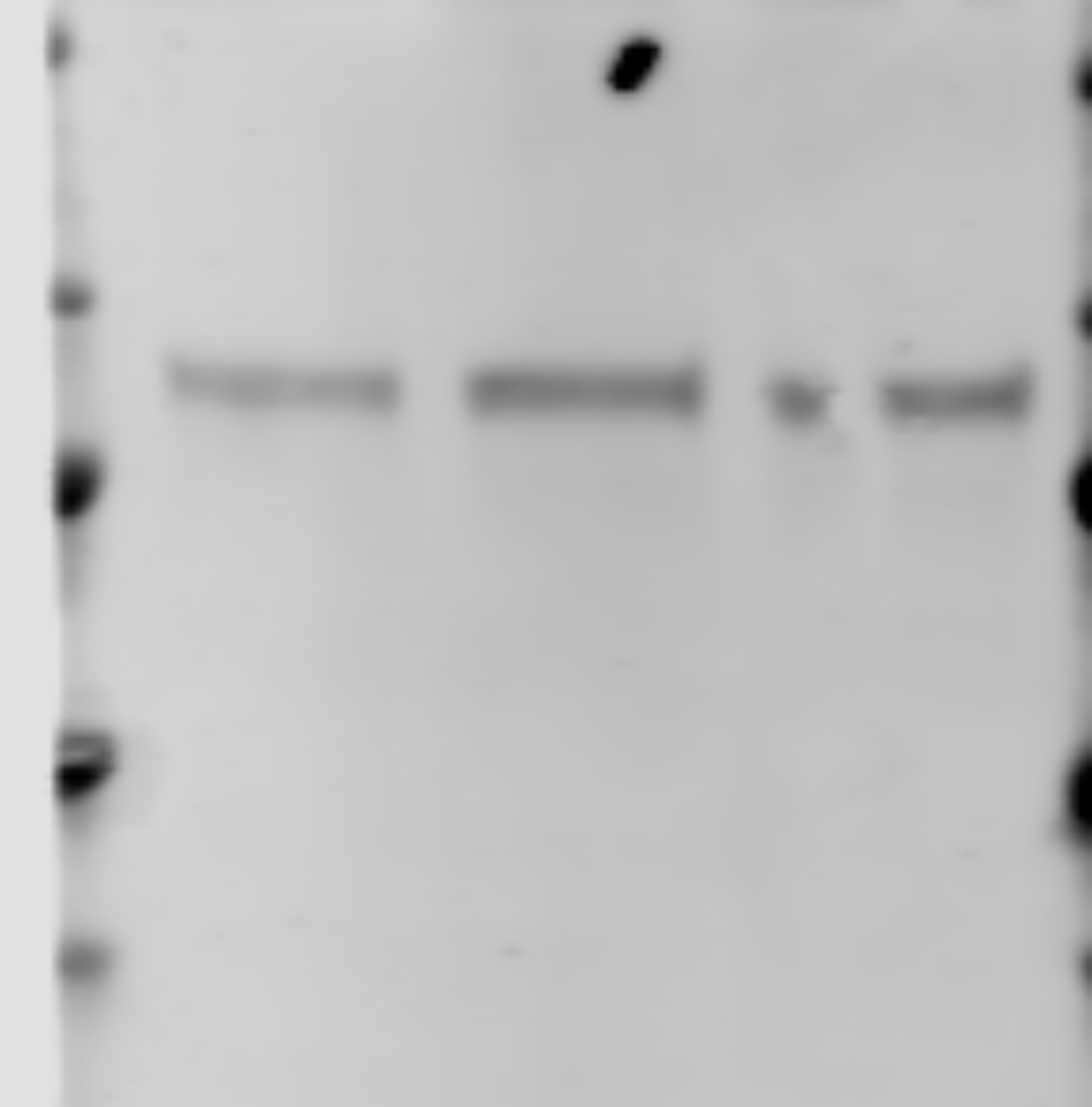

Supplement: Supplementary file 5 — Source data Fig. 4 [file 44318_2025_537_MOESM5_ESM.zip › EMBOJ-2025-120849-T_Source data Fig_4/Fig_4B/Images_Fig_4B/GCN5.tif]

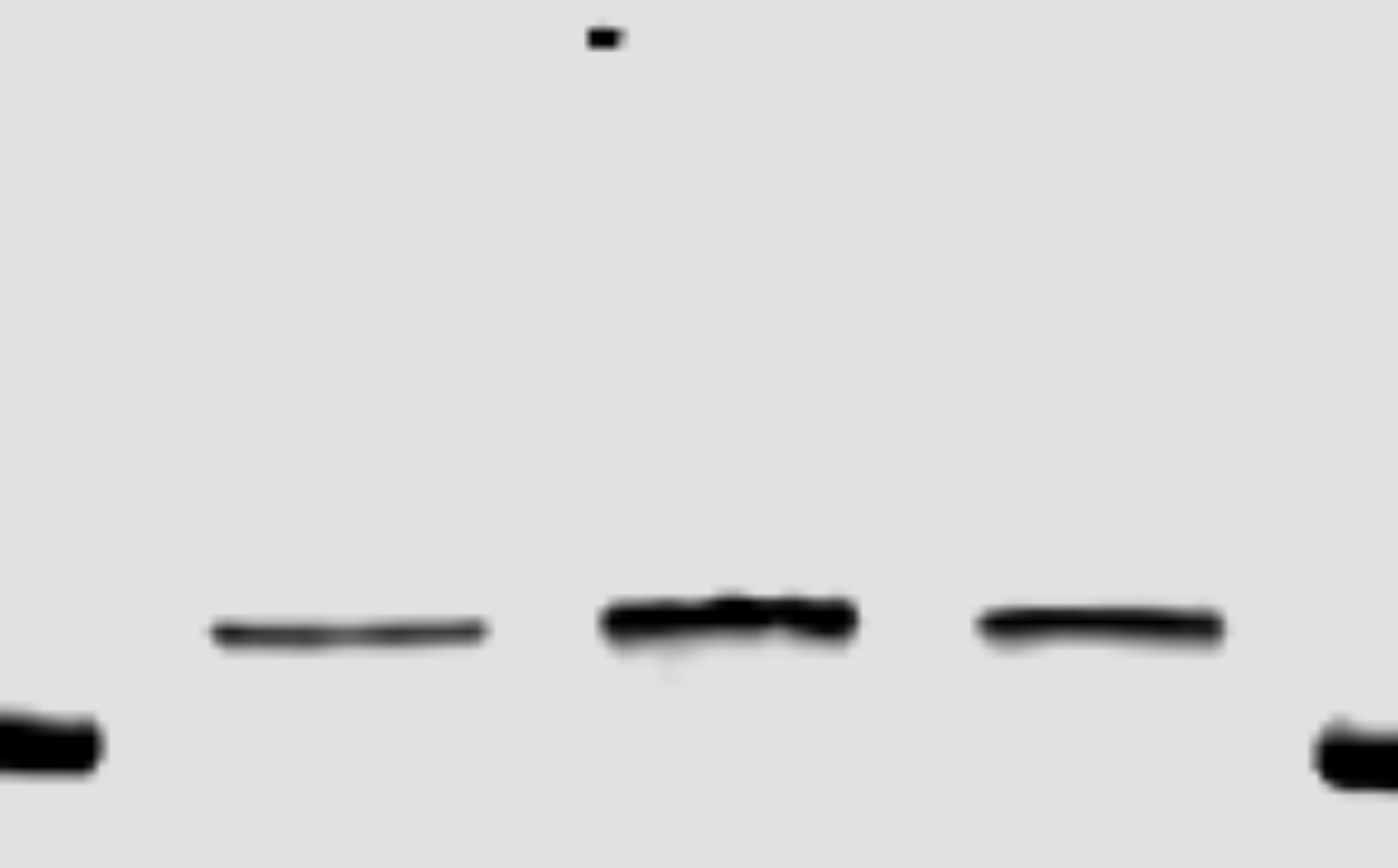

Supplement: Supplementary file 5 — Source data Fig. 4 [file 44318_2025_537_MOESM5_ESM.zip › EMBOJ-2025-120849-T_Source data Fig_4/Fig_4B/Images_Fig_4B/p300.tif]

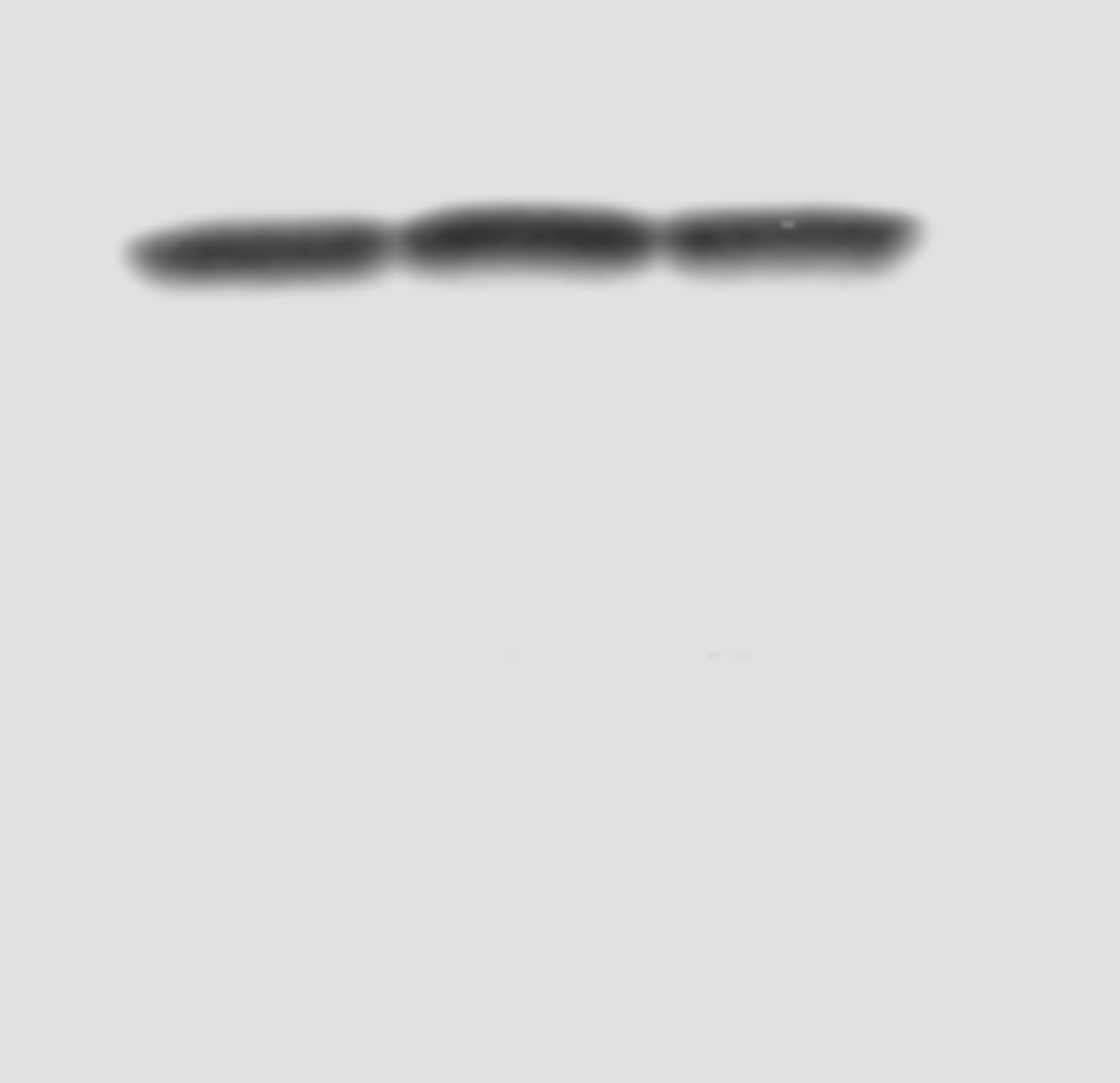

Supplement: Supplementary file 5 — Source data Fig. 4 [file 44318_2025_537_MOESM5_ESM.zip › EMBOJ-2025-120849-T_Source data Fig_4/Fig_4B/Images_Fig_4B/Histone_H3_Ctrl_for_GCN5.tif]
