# Supplementary material for: Crosstalk between chromatin state and ATM signalling in DNA damage-induced transcription stress
Source: EMBO J. 2025 Aug 26;44(19):5564–94. doi: 10.1038/s44318-025-00537-7 (PMC12489091; doi:10.1038/s44318-025-00537-7)
Supplement: Supplementary file 4 — Source data Fig. 3 [file 44318_2025_537_MOESM4_ESM.zip › EMBOJ-2025-120849-T_Source data Fig_3/Fig_3B/readme_Fig_3B.docx]

**Proximity Ligation Assay (PLA) between p300 and CPD in UV-irradiated cells (0, 5, 10, and 20 J/m²) (Figure 3B)**

**Folder Contents:**This folder contains microscopy images (in “Images” subfolder) and quantification data (Excel file) corresponding to the PLA analysis shown in Figure 3B of the manuscript.

**Image Acquisition:**

- Confocal microscopy images were acquired using a Zeiss LSM700 laser-scanning confocal microscope at a resolution of 512 × 512 pixels.
- Images were exported as TIFF files directly from ZEN software.
- This acquisition setting was optimized for high-throughput imaging across multiple UV doses.
- No image processing, filtering, or resolution downsampling was performed following acquisition.

**Quantification and Analysis:**

- PLA signal quantification was performed using multiple unmodified TIFF images in Fiji (Image J).
- Foci numbers were normalized to the mean foci of non-irradiated cells (0 J/m²) in Excel.
- Graphs and statistical analyses were generated using GraphPad Prism, with full details provided in the accompanying Excel file.

**Figure Presentation Notes:**

- Brightness and contrast adjustments were applied uniformly only to the representative figure panels for visualization purposes.
- These adjustments were applied identically across all conditions.
- No adjustments were made to the images used for quantification.
