# Supplementary material for: Crosstalk between chromatin state and ATM signalling in DNA damage-induced transcription stress
Source: EMBO J. 2025 Aug 26;44(19):5564–94. doi: 10.1038/s44318-025-00537-7 (PMC12489091; doi:10.1038/s44318-025-00537-7)
Supplement: Supplementary file 4 — Source data Fig. 3 [file 44318_2025_537_MOESM4_ESM.zip › EMBOJ-2025-120849-T_Source data Fig_3/Fig_3C/readme_Fig_3C.docx]

**Co-localization of p300, PCAF and GCN5 HATs with CPDs, following micropore filter UV irradiation (Figure 3C)**

**Folder Contents:**This folder contains microscopy images corresponding to the Figure 3C of the manuscript.

**Image Acquisition and processing:**

- Confocal microscopy images were acquired using a Zeiss LSM700 laser-scanning confocal microscope.
- Images were exported as TIFF files directly from ZEN software.
- Brightness and contrast adjustments were applied uniformly only to the figure panels in the manuscript for visualization purposes.
- These adjustments were applied identically across all conditions.
