# Supplementary material for: Crosstalk between chromatin state and ATM signalling in DNA damage-induced transcription stress
Source: EMBO J. 2025 Aug 26;44(19):5564–94. doi: 10.1038/s44318-025-00537-7 (PMC12489091; doi:10.1038/s44318-025-00537-7)
Supplement: Supplementary file 5 — Source data Fig. 4 [file 44318_2025_537_MOESM5_ESM.zip › EMBOJ-2025-120849-T_Source data Fig_4/Fig_4A/readme_Fig_4A.docx]

**Proximity Ligation Assays (PLA) between p300–CPD, PCAF–CPD, and GCN5–CPD in UV-irradiated cells ± THZ1 (Figure 4A)**

**Folder Contents:**
This folder contains microscopy images (“Images” subfolder) and quantitative data (Excel file) corresponding to the PLA experiments shown in Figure 4A of the manuscript.

**Image Acquisition:**

- Images were acquired using a Zeiss LSM700 laser-scanning confocal microscope at a resolution of 512 × 512 pixels with acquisition settings optimized for high-throughput across multiple protein targets and treatment conditions.
- Files were exported as TIFF files directly from ZEN software.
- No post-acquisition image processing (e.g., filtering, resolution adjustment, or cropping) was applied to the files used for quantification.

**Quantification and Analysis:**

- PLA signal intensities were quantified from multiple unmodified TIFF images using Fiji (ImageJ), and the data were normalized to control samples in Excel.
- Graphs and statistical comparisons were generated using GraphPad Prism, with full details provided in the accompanying Excel file.

**Figure Presentation Notes:**

- Brightness and contrast adjustments were applied only to the representative images shown in the figure panel, to enhance visibility.
- These adjustments were applied uniformly across all experimental conditions.
- Images used for quantification remained unaltered.
