# Supplementary material for: Crosstalk between chromatin state and ATM signalling in DNA damage-induced transcription stress
Source: EMBO J. 2025 Aug 26;44(19):5564–94. doi: 10.1038/s44318-025-00537-7 (PMC12489091; doi:10.1038/s44318-025-00537-7)
Supplement: Supplementary file 5 — Source data Fig. 4 [file 44318_2025_537_MOESM5_ESM.zip › EMBOJ-2025-120849-T_Source data Fig_4/Fig_4B/readme_Fig_4B.docx]

**Immunoblots showing chromatin association of HATs (PCAF and p300) in UV-irradiated cells ± THZ1 (Figure 4B)**

**Folder Contents:**
This folder contains source data for the immunoblots presented in Figure 4B of the manuscript, including images (“Images” subfolder) and quantification (Excel file).

**Image Acquisition and Processing:**

- Immunoblot images were acquired using an Odyssey CLx (*LI-COR)* imaging system.
- Image intensity levels were adjusted prior to quantification to ensure grayscale rendering and avoid saturation. These adjustments were applied uniformly across the membrane and did not alter the relative signal intensities.
- Images were exported as TIFF files directly from the *LI-COR* Image Studio 6.0 Software.

**Blotting and Antibody Incubation Details:**

- Membranes were cut prior to antibody incubation to allow separate hybridization with different antibodies.
- PCAF and p300 were detected on the same (cropped) membrane and share a common Histone H3 loading control (also detected on the same membrane). GCN5 and its corresponding Histone H3 control were detected on the same (cropped) membrane.

**Quantification and Analysis:**

- Signal intensities were measured using ImageQuant software.
- Values were normalized to histone H3 levels and expressed as fold change relative to non-irradiated controls.
- The accompanying Excel file contains:
  - Raw and normalized quantification data
  - Cropped versions of the blot images corresponding to the final figure panel (included for reference only)
  - Uncropped images
