# Supplementary figures and images for: Crosstalk between chromatin state and ATM signalling in DNA damage-induced transcription stress (part 2 of 2)
Source: EMBO J. 2025 Aug 26;44(19):5564–94. doi: 10.1038/s44318-025-00537-7 (PMC12489091; doi:10.1038/s44318-025-00537-7)

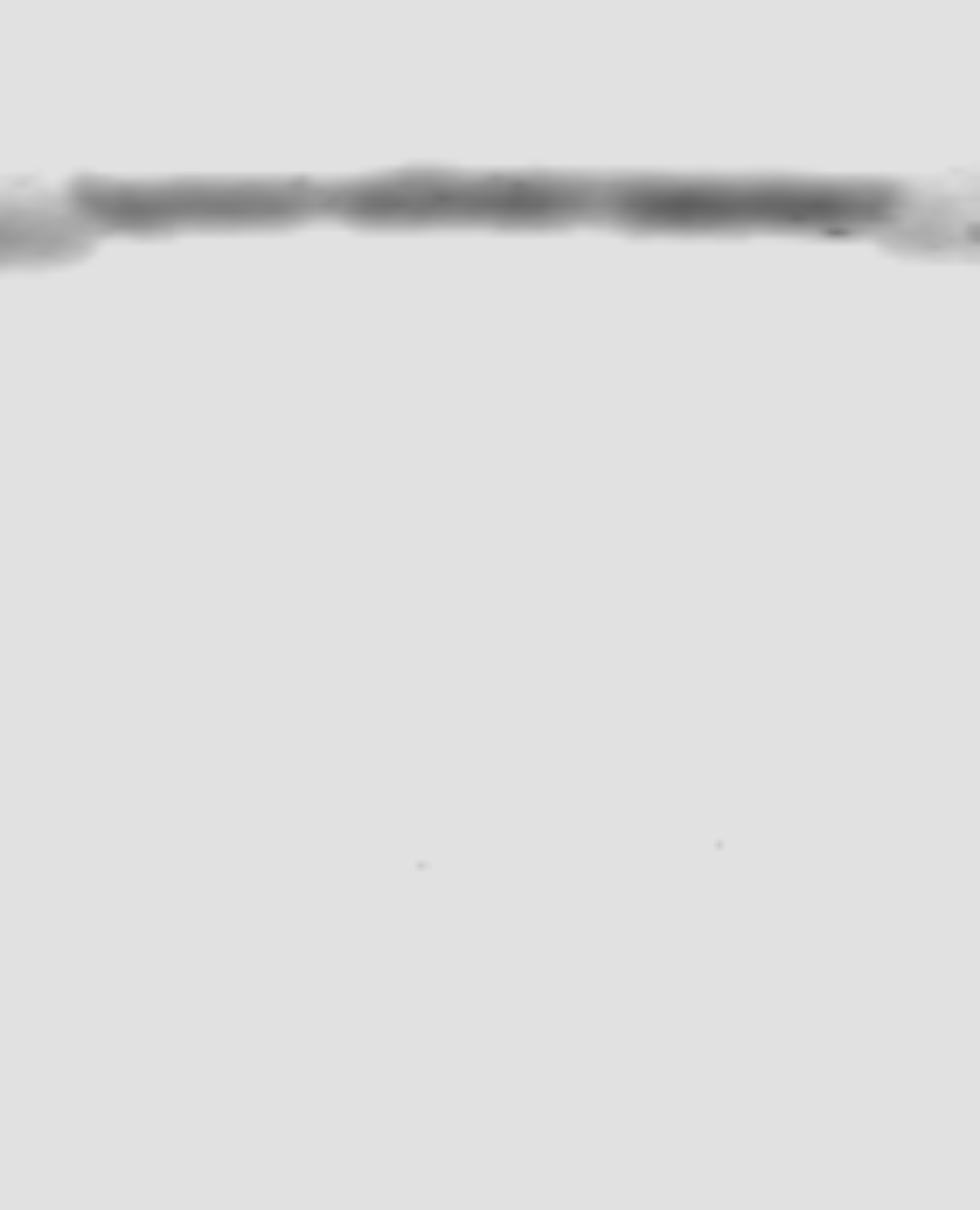

Supplement: Supplementary file 5 — Source data Fig. 4 [file 44318_2025_537_MOESM5_ESM.zip › EMBOJ-2025-120849-T_Source data Fig_4/Fig_4B/Images_Fig_4B/Histone_H3_Ctrl_for_p300_and_PCAF.tif]

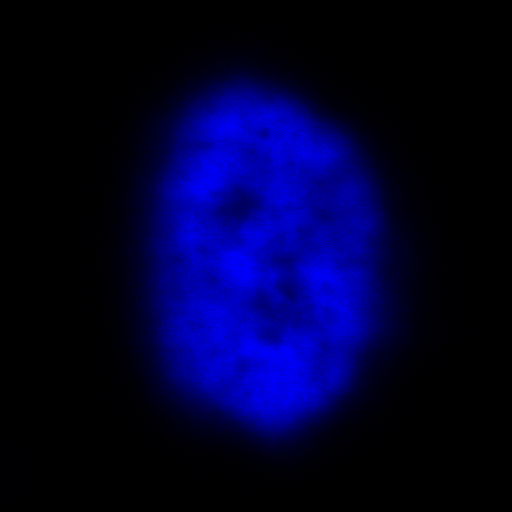

Supplement: Supplementary file 5 — Source data Fig. 4 [file 44318_2025_537_MOESM5_ESM.zip › EMBOJ-2025-120849-T_Source data Fig_4/Fig_4C/Images_Fig_4C/DAPI_THZ1+UV_magnification.tif]

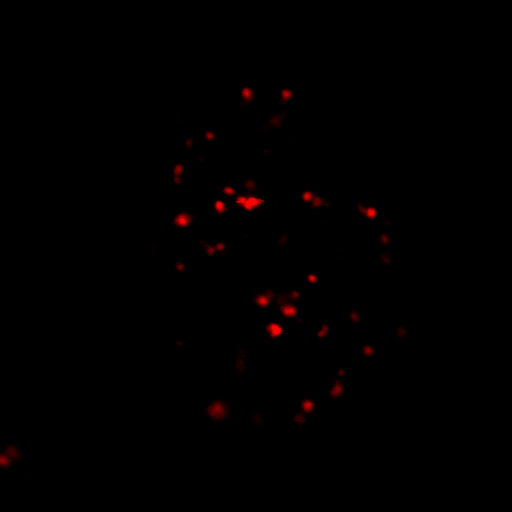

Supplement: Supplementary file 5 — Source data Fig. 4 [file 44318_2025_537_MOESM5_ESM.zip › EMBOJ-2025-120849-T_Source data Fig_4/Fig_4C/Images_Fig_4C/PLA_THZ1+UV_magnification.tif]

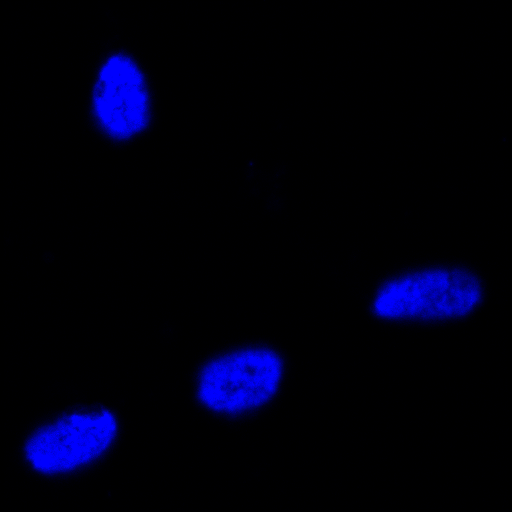

Supplement: Supplementary file 5 — Source data Fig. 4 [file 44318_2025_537_MOESM5_ESM.zip › EMBOJ-2025-120849-T_Source data Fig_4/Fig_4C/Images_Fig_4C/DAPI_THZ1+UV.tif]

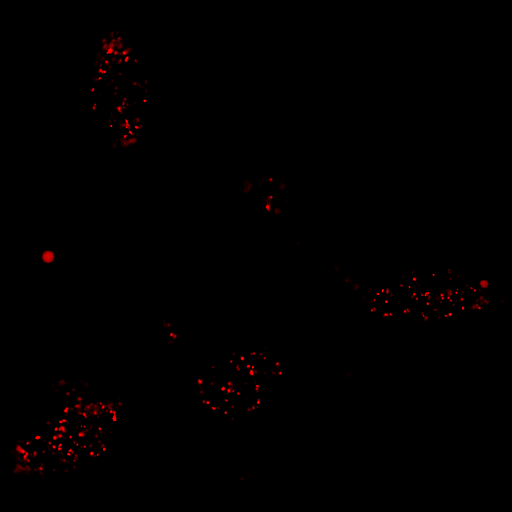

Supplement: Supplementary file 5 — Source data Fig. 4 [file 44318_2025_537_MOESM5_ESM.zip › EMBOJ-2025-120849-T_Source data Fig_4/Fig_4C/Images_Fig_4C/PLA_THZ1+UV.tif]

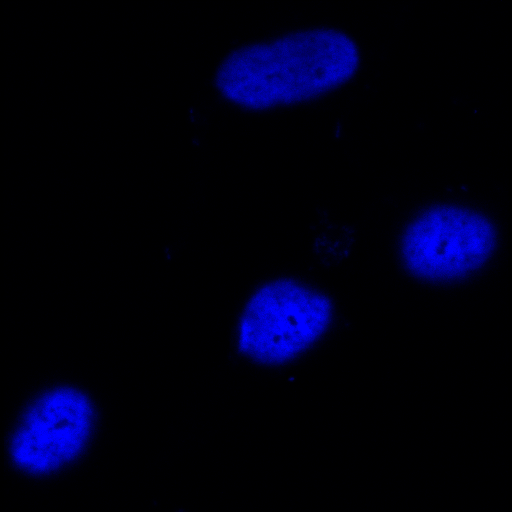

Supplement: Supplementary file 5 — Source data Fig. 4 [file 44318_2025_537_MOESM5_ESM.zip › EMBOJ-2025-120849-T_Source data Fig_4/Fig_4C/Images_Fig_4C/DAPI_untreated.tif]

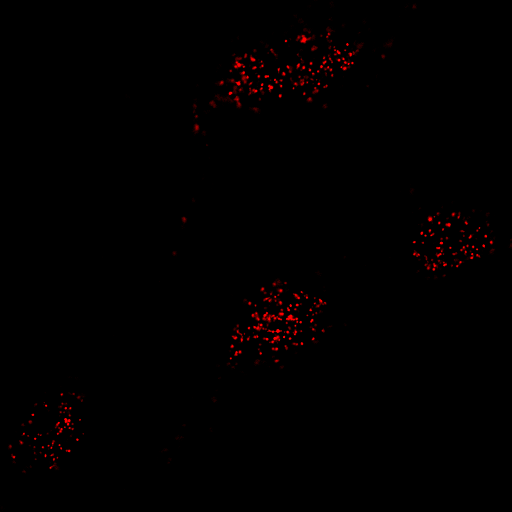

Supplement: Supplementary file 5 — Source data Fig. 4 [file 44318_2025_537_MOESM5_ESM.zip › EMBOJ-2025-120849-T_Source data Fig_4/Fig_4C/Images_Fig_4C/PLA_untreated.tif]

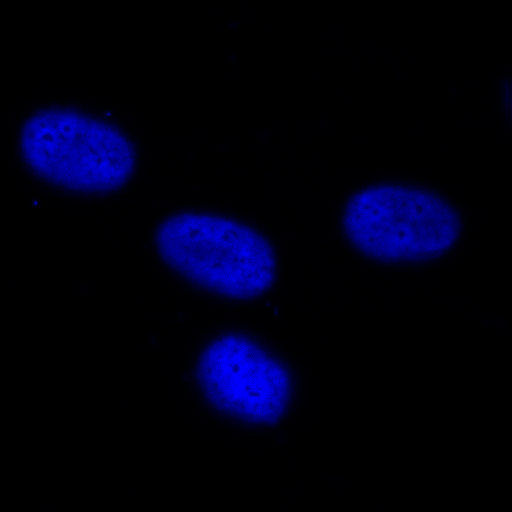

Supplement: Supplementary file 5 — Source data Fig. 4 [file 44318_2025_537_MOESM5_ESM.zip › EMBOJ-2025-120849-T_Source data Fig_4/Fig_4C/Images_Fig_4C/UV_DAPI.tif]

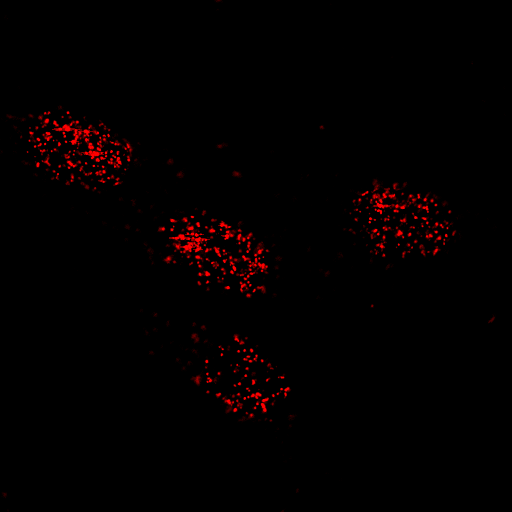

Supplement: Supplementary file 5 — Source data Fig. 4 [file 44318_2025_537_MOESM5_ESM.zip › EMBOJ-2025-120849-T_Source data Fig_4/Fig_4C/Images_Fig_4C/UV_PLA.tif]

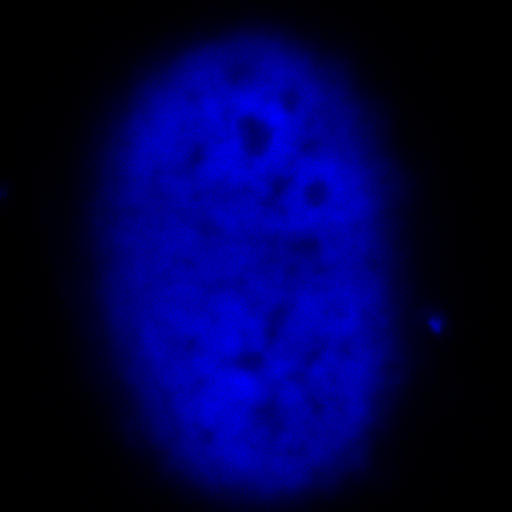

Supplement: Supplementary file 5 — Source data Fig. 4 [file 44318_2025_537_MOESM5_ESM.zip › EMBOJ-2025-120849-T_Source data Fig_4/Fig_4C/Images_Fig_4C/DAPI_UV_magnification.tif]

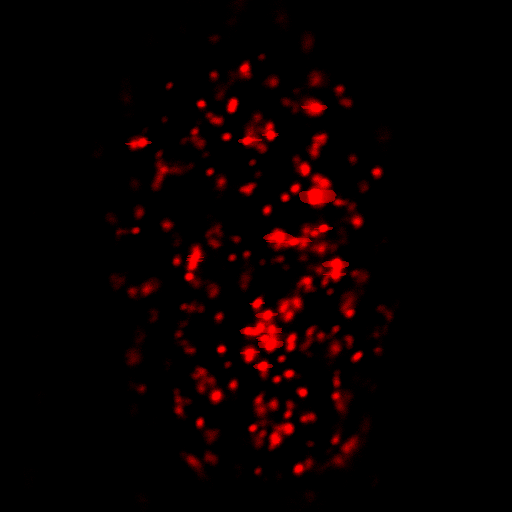

Supplement: Supplementary file 5 — Source data Fig. 4 [file 44318_2025_537_MOESM5_ESM.zip › EMBOJ-2025-120849-T_Source data Fig_4/Fig_4C/Images_Fig_4C/PLA_UV_magnification.tif]

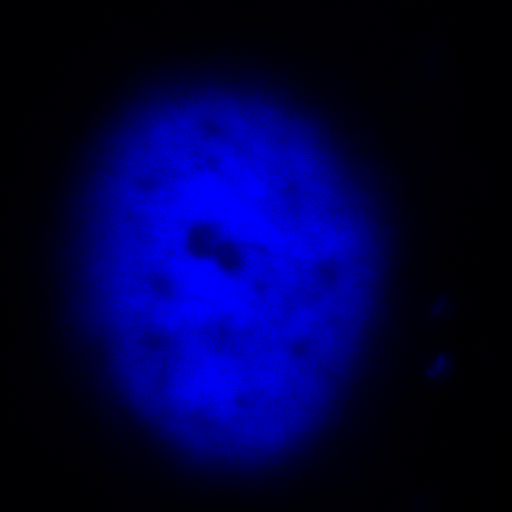

Supplement: Supplementary file 5 — Source data Fig. 4 [file 44318_2025_537_MOESM5_ESM.zip › EMBOJ-2025-120849-T_Source data Fig_4/Fig_4C/Images_Fig_4C/DAPI_untreated_magnification.tif]

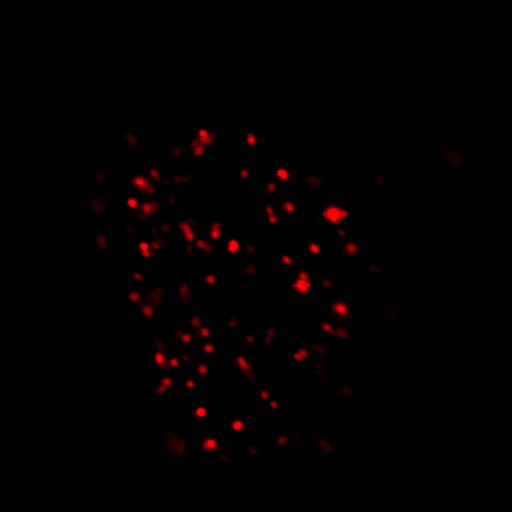

Supplement: Supplementary file 5 — Source data Fig. 4 [file 44318_2025_537_MOESM5_ESM.zip › EMBOJ-2025-120849-T_Source data Fig_4/Fig_4C/Images_Fig_4C/PLA_untreated_magnification.tif]

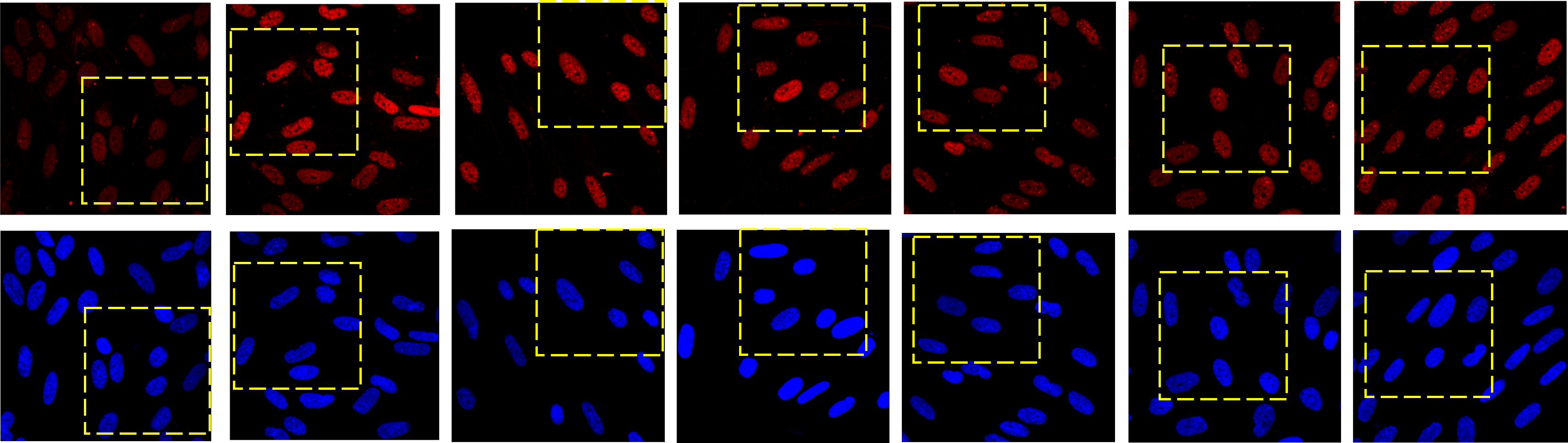

Supplement: Supplementary file 6 — Source data Fig. 5 [file 44318_2025_537_MOESM6_ESM.zip › EMBOJ-2025-120849-T_Source data Fig_5/Fig_5C/Images_Fig_5C/All_uncropped_images.tif]

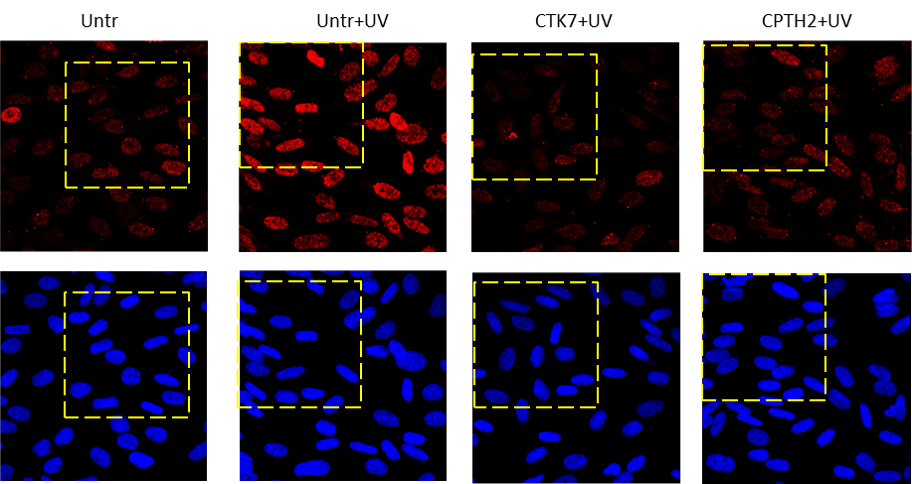

Supplement: Supplementary file 6 — Source data Fig. 5 [file 44318_2025_537_MOESM6_ESM.zip › EMBOJ-2025-120849-T_Source data Fig_5/Fig_5D/Images_Fig_5D/All_uncropped_images.tif]

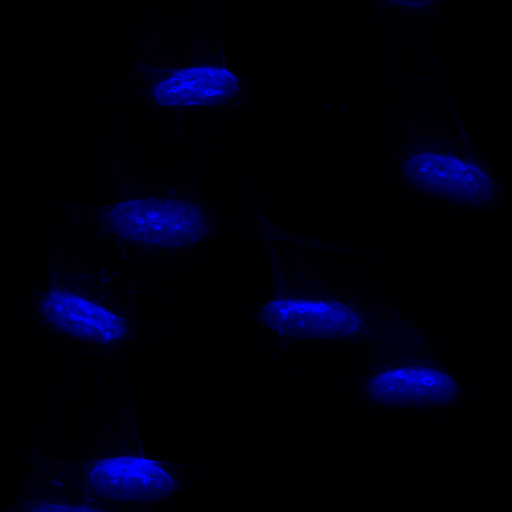

Supplement: Supplementary file 6 — Source data Fig. 5 [file 44318_2025_537_MOESM6_ESM.zip › EMBOJ-2025-120849-T_Source data Fig_5/Fig_5B/Images_Fig_5B/DAPI_CTK7+UV.tif]

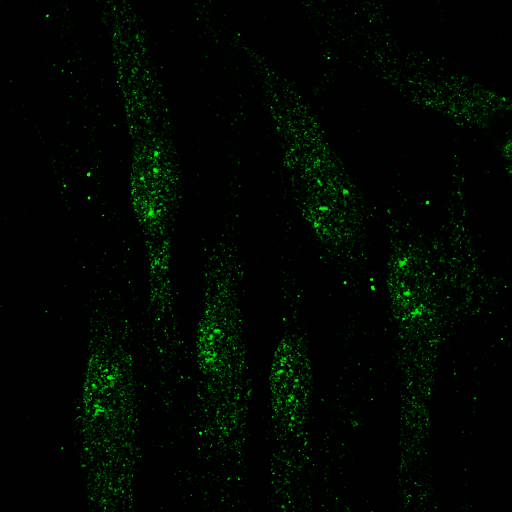

Supplement: Supplementary file 6 — Source data Fig. 5 [file 44318_2025_537_MOESM6_ESM.zip › EMBOJ-2025-120849-T_Source data Fig_5/Fig_5B/Images_Fig_5B/S9.6_THZ1.tif]

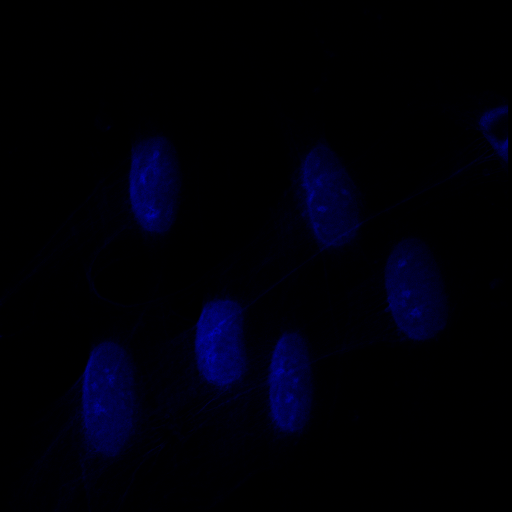

Supplement: Supplementary file 6 — Source data Fig. 5 [file 44318_2025_537_MOESM6_ESM.zip › EMBOJ-2025-120849-T_Source data Fig_5/Fig_5B/Images_Fig_5B/DAPI_THZ1.tif]

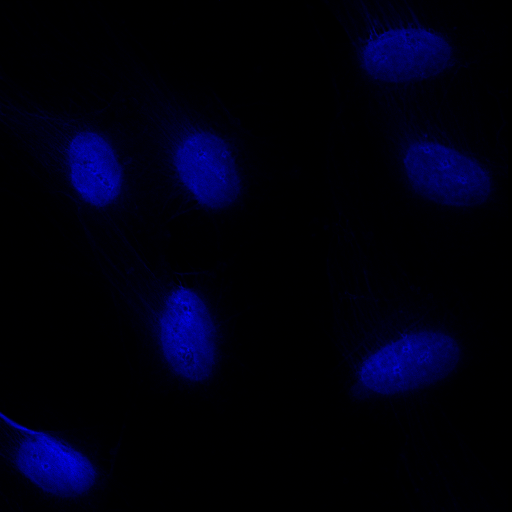

Supplement: Supplementary file 6 — Source data Fig. 5 [file 44318_2025_537_MOESM6_ESM.zip › EMBOJ-2025-120849-T_Source data Fig_5/Fig_5B/Images_Fig_5B/DAPI_UV.tif]

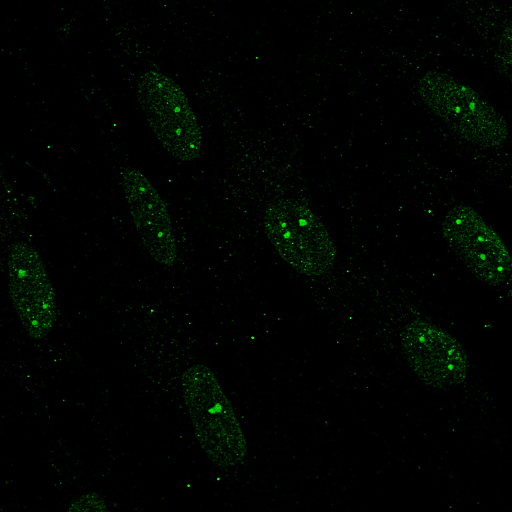

Supplement: Supplementary file 6 — Source data Fig. 5 [file 44318_2025_537_MOESM6_ESM.zip › EMBOJ-2025-120849-T_Source data Fig_5/Fig_5B/Images_Fig_5B/S9.6_untreated.tif]

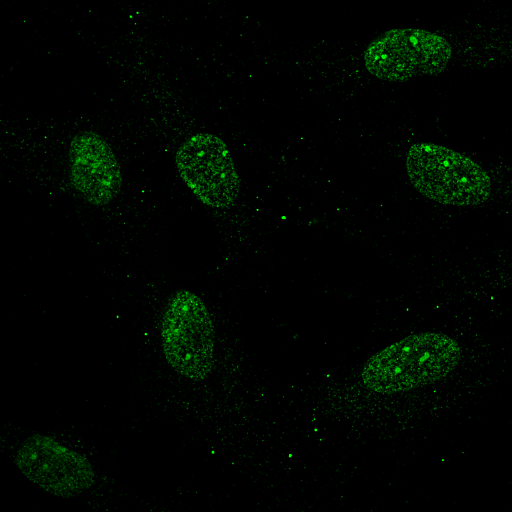

Supplement: Supplementary file 6 — Source data Fig. 5 [file 44318_2025_537_MOESM6_ESM.zip › EMBOJ-2025-120849-T_Source data Fig_5/Fig_5B/Images_Fig_5B/S9.6_UV.tif]

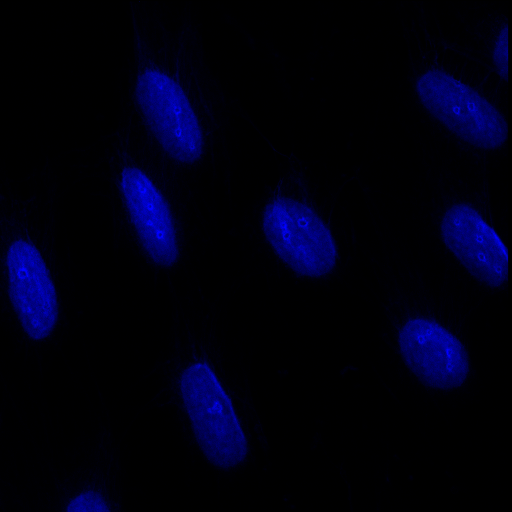

Supplement: Supplementary file 6 — Source data Fig. 5 [file 44318_2025_537_MOESM6_ESM.zip › EMBOJ-2025-120849-T_Source data Fig_5/Fig_5B/Images_Fig_5B/DAPI_untreated.tif]

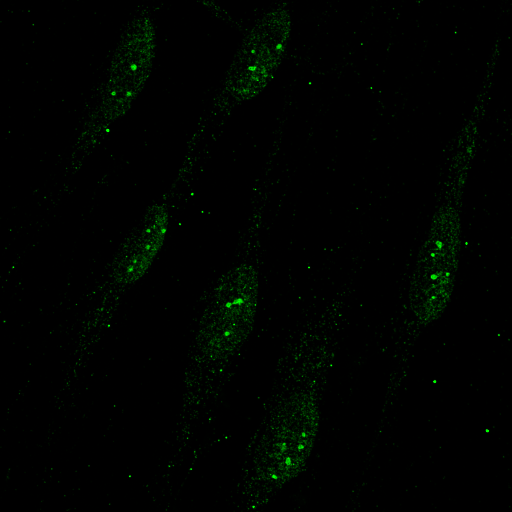

Supplement: Supplementary file 6 — Source data Fig. 5 [file 44318_2025_537_MOESM6_ESM.zip › EMBOJ-2025-120849-T_Source data Fig_5/Fig_5B/Images_Fig_5B/S9.6_CPTH2+UV.tif]

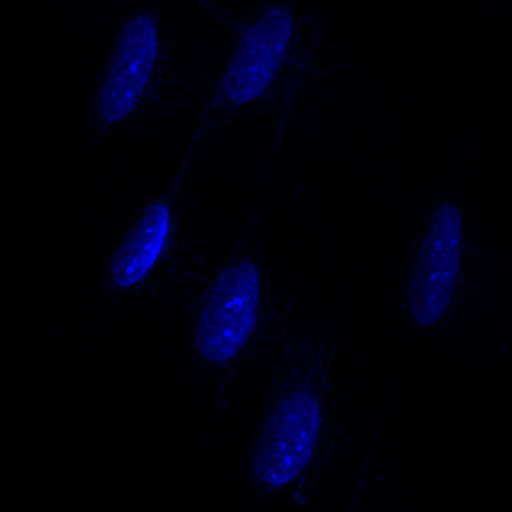

Supplement: Supplementary file 6 — Source data Fig. 5 [file 44318_2025_537_MOESM6_ESM.zip › EMBOJ-2025-120849-T_Source data Fig_5/Fig_5B/Images_Fig_5B/DAPI_CPTH+UV.tif]

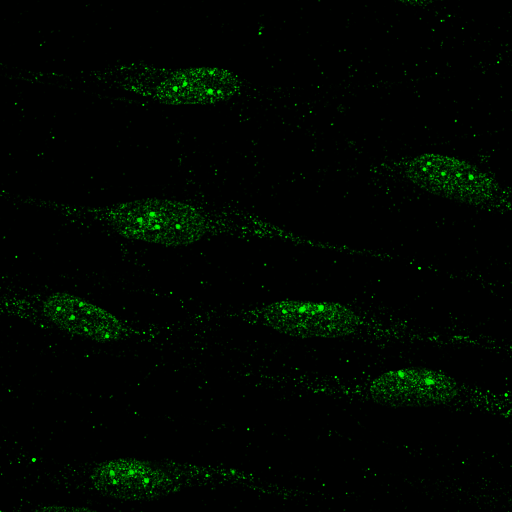

Supplement: Supplementary file 6 — Source data Fig. 5 [file 44318_2025_537_MOESM6_ESM.zip › EMBOJ-2025-120849-T_Source data Fig_5/Fig_5B/Images_Fig_5B/S9.6_CTK7+UV.tif]

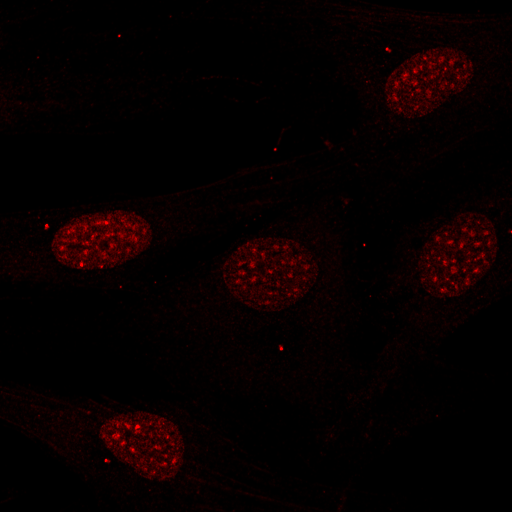

Supplement: Supplementary file 6 — Source data Fig. 5 [file 44318_2025_537_MOESM6_ESM.zip › EMBOJ-2025-120849-T_Source data Fig_5/Fig_5F/Images_Fig_5F/P-ATM_UV.tif]

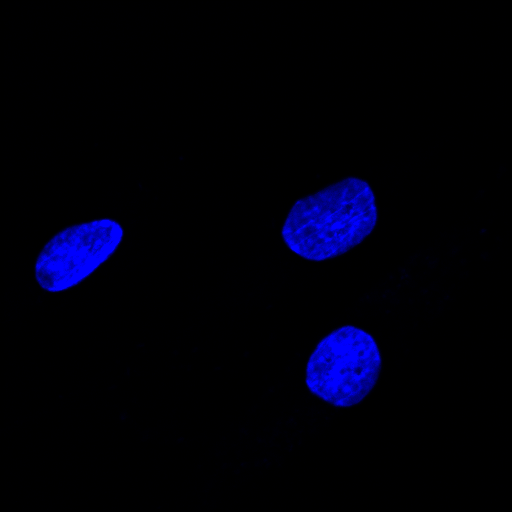

Supplement: Supplementary file 6 — Source data Fig. 5 [file 44318_2025_537_MOESM6_ESM.zip › EMBOJ-2025-120849-T_Source data Fig_5/Fig_5F/Images_Fig_5F/DAPI_TSA.tif]

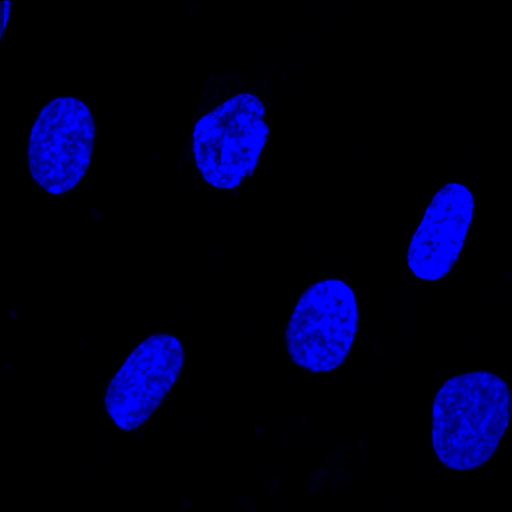

Supplement: Supplementary file 6 — Source data Fig. 5 [file 44318_2025_537_MOESM6_ESM.zip › EMBOJ-2025-120849-T_Source data Fig_5/Fig_5F/Images_Fig_5F/DAPI_untr.tif]

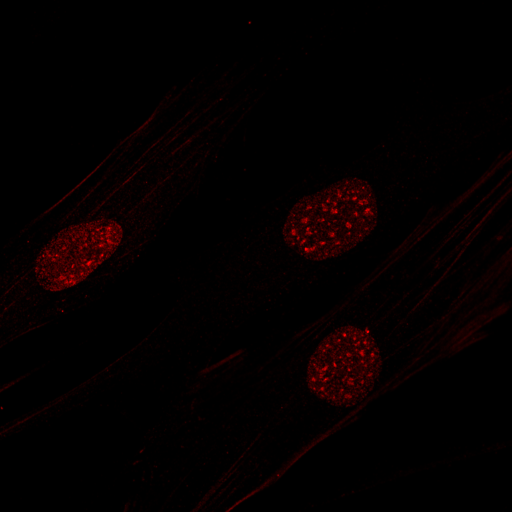

Supplement: Supplementary file 6 — Source data Fig. 5 [file 44318_2025_537_MOESM6_ESM.zip › EMBOJ-2025-120849-T_Source data Fig_5/Fig_5F/Images_Fig_5F/P-ATM_TSA.tif]

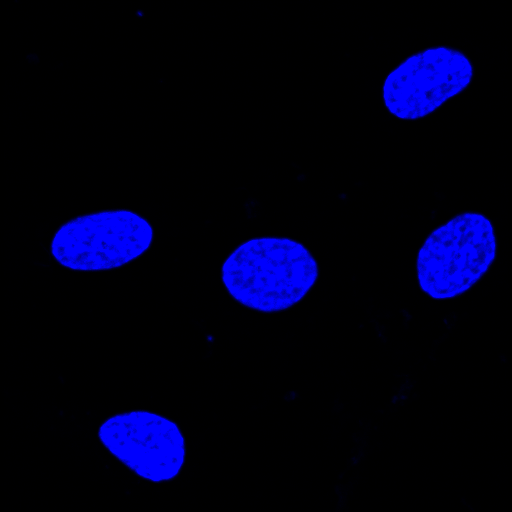

Supplement: Supplementary file 6 — Source data Fig. 5 [file 44318_2025_537_MOESM6_ESM.zip › EMBOJ-2025-120849-T_Source data Fig_5/Fig_5F/Images_Fig_5F/DAPI_UV.tif]

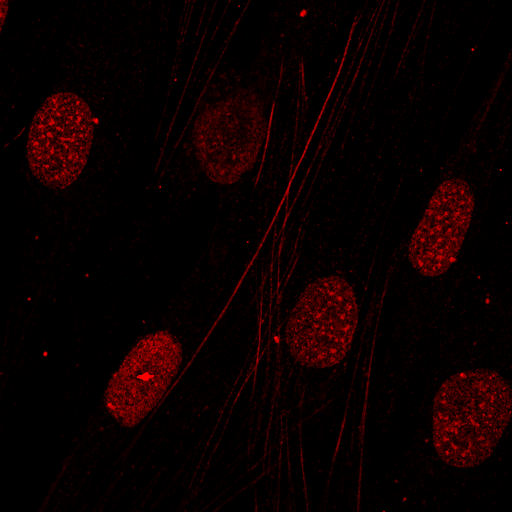

Supplement: Supplementary file 6 — Source data Fig. 5 [file 44318_2025_537_MOESM6_ESM.zip › EMBOJ-2025-120849-T_Source data Fig_5/Fig_5F/Images_Fig_5F/P-ATM_untr.tif]

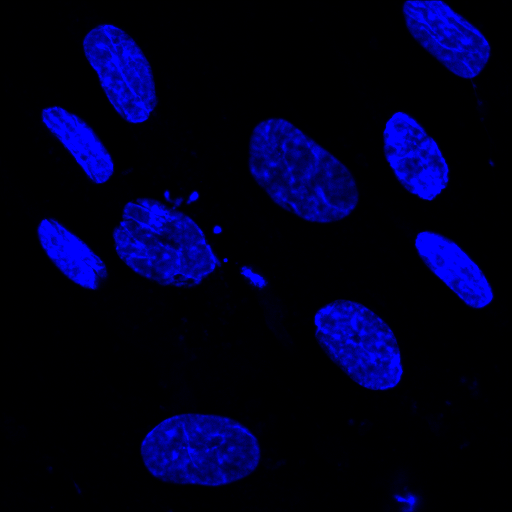

Supplement: Supplementary file 6 — Source data Fig. 5 [file 44318_2025_537_MOESM6_ESM.zip › EMBOJ-2025-120849-T_Source data Fig_5/Fig_5G/Images_Fig_5G/DAPI_UV.tif]

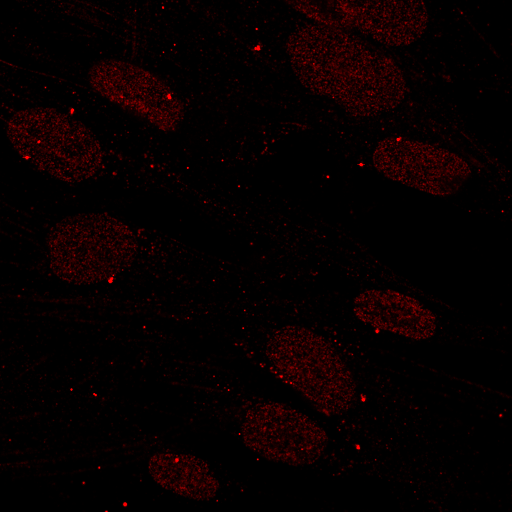

Supplement: Supplementary file 6 — Source data Fig. 5 [file 44318_2025_537_MOESM6_ESM.zip › EMBOJ-2025-120849-T_Source data Fig_5/Fig_5G/Images_Fig_5G/P-ATM_BIX.tif]

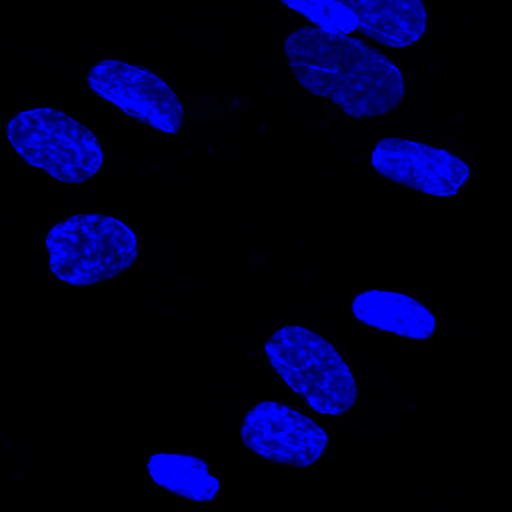

Supplement: Supplementary file 6 — Source data Fig. 5 [file 44318_2025_537_MOESM6_ESM.zip › EMBOJ-2025-120849-T_Source data Fig_5/Fig_5G/Images_Fig_5G/DAPI_BIX.tif]

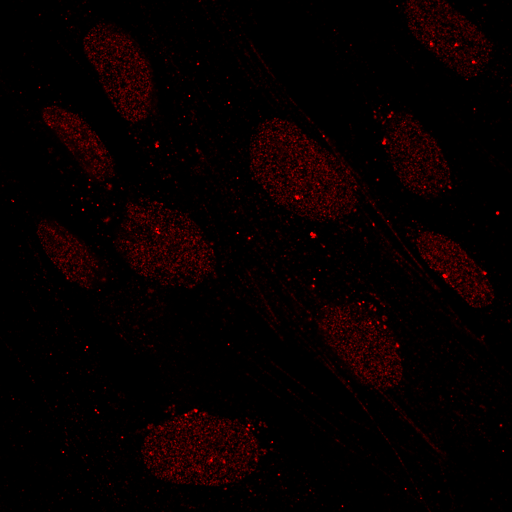

Supplement: Supplementary file 6 — Source data Fig. 5 [file 44318_2025_537_MOESM6_ESM.zip › EMBOJ-2025-120849-T_Source data Fig_5/Fig_5G/Images_Fig_5G/P-ATM_UV.tif]

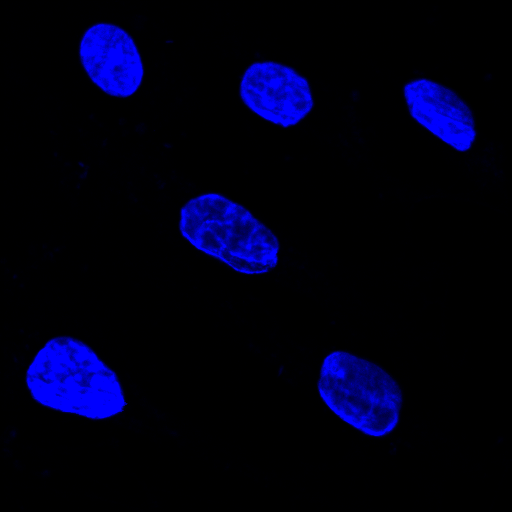

Supplement: Supplementary file 6 — Source data Fig. 5 [file 44318_2025_537_MOESM6_ESM.zip › EMBOJ-2025-120849-T_Source data Fig_5/Fig_5G/Images_Fig_5G/DAPI_GSK126.tif]

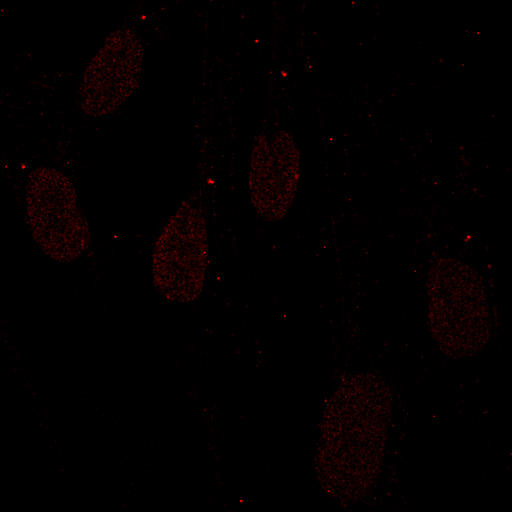

Supplement: Supplementary file 6 — Source data Fig. 5 [file 44318_2025_537_MOESM6_ESM.zip › EMBOJ-2025-120849-T_Source data Fig_5/Fig_5G/Images_Fig_5G/P-ATM_untr.tif]

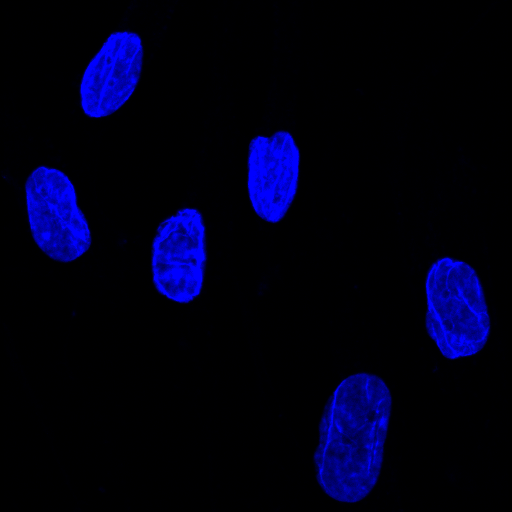

Supplement: Supplementary file 6 — Source data Fig. 5 [file 44318_2025_537_MOESM6_ESM.zip › EMBOJ-2025-120849-T_Source data Fig_5/Fig_5G/Images_Fig_5G/DAPI_untr.tif]

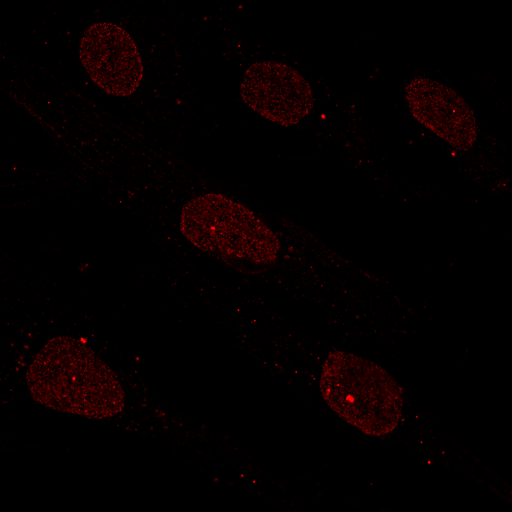

Supplement: Supplementary file 6 — Source data Fig. 5 [file 44318_2025_537_MOESM6_ESM.zip › EMBOJ-2025-120849-T_Source data Fig_5/Fig_5G/Images_Fig_5G/P-ATM_GSK126.tif]

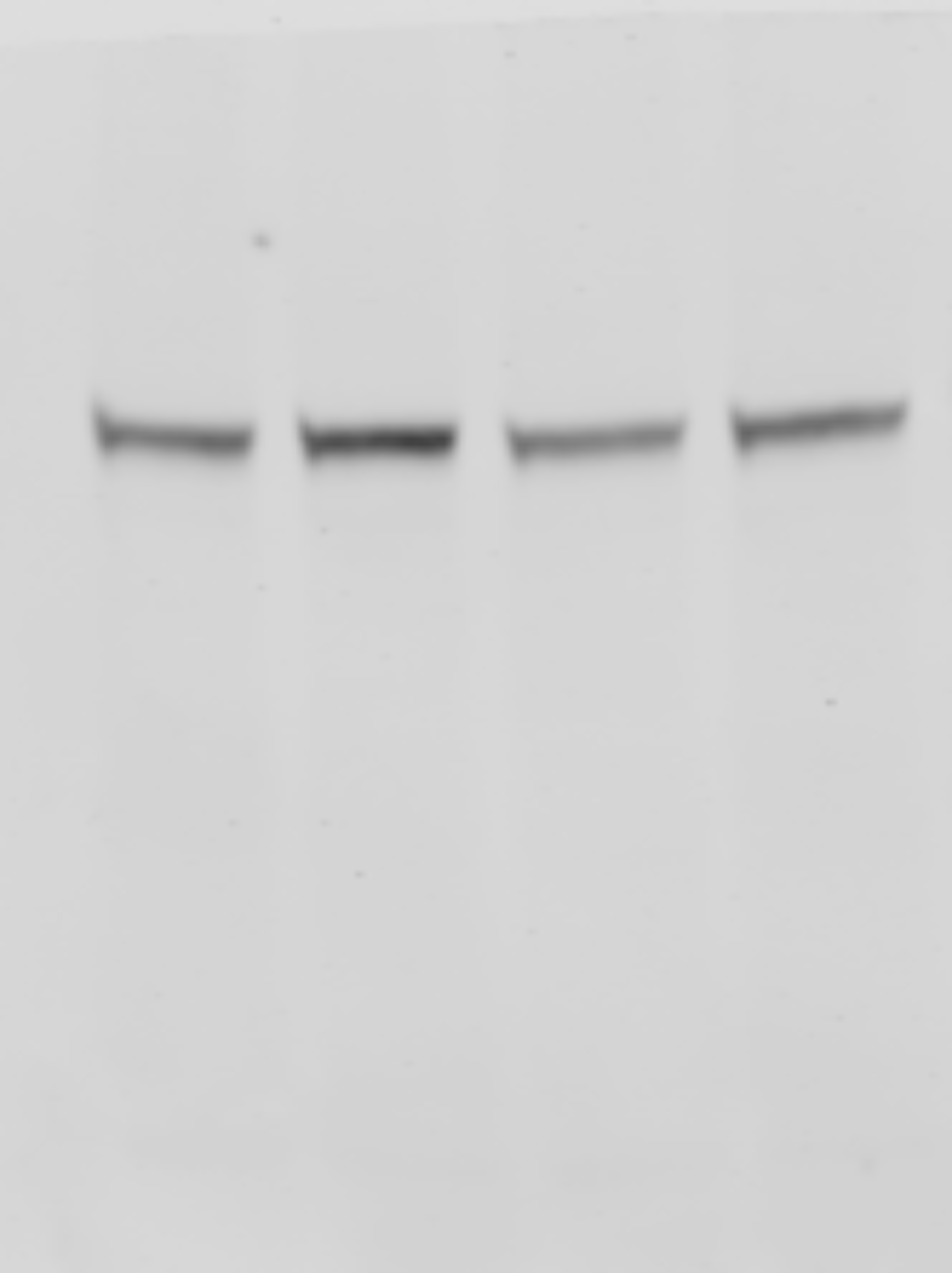

Supplement: Supplementary file 6 — Source data Fig. 5 [file 44318_2025_537_MOESM6_ESM.zip › EMBOJ-2025-120849-T_Source data Fig_5/Fig_5E/Images_Fig_5E/XAB2_HATi.tif]

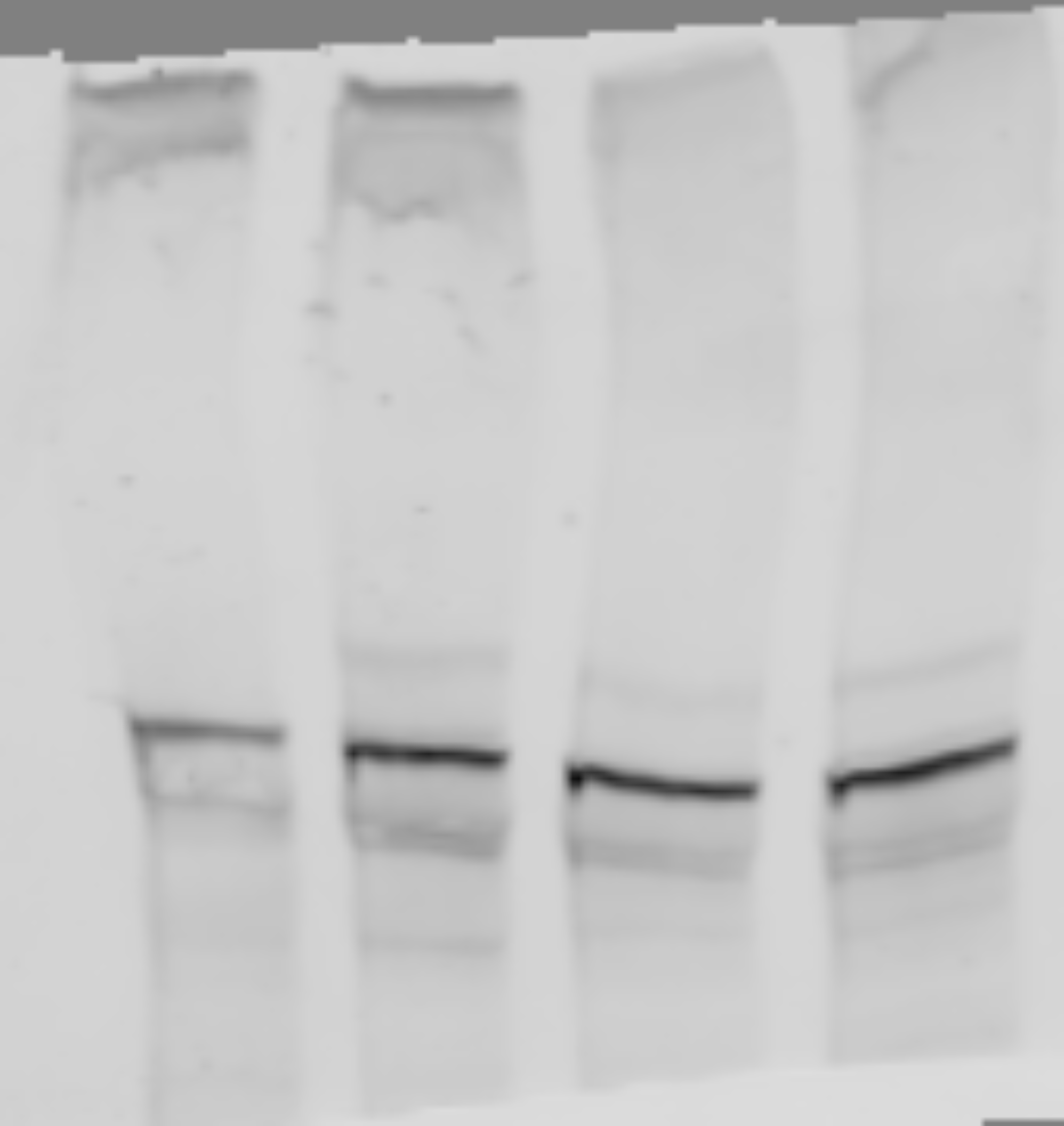

Supplement: Supplementary file 6 — Source data Fig. 5 [file 44318_2025_537_MOESM6_ESM.zip › EMBOJ-2025-120849-T_Source data Fig_5/Fig_5E/Images_Fig_5E/ATM_HATi.tif]

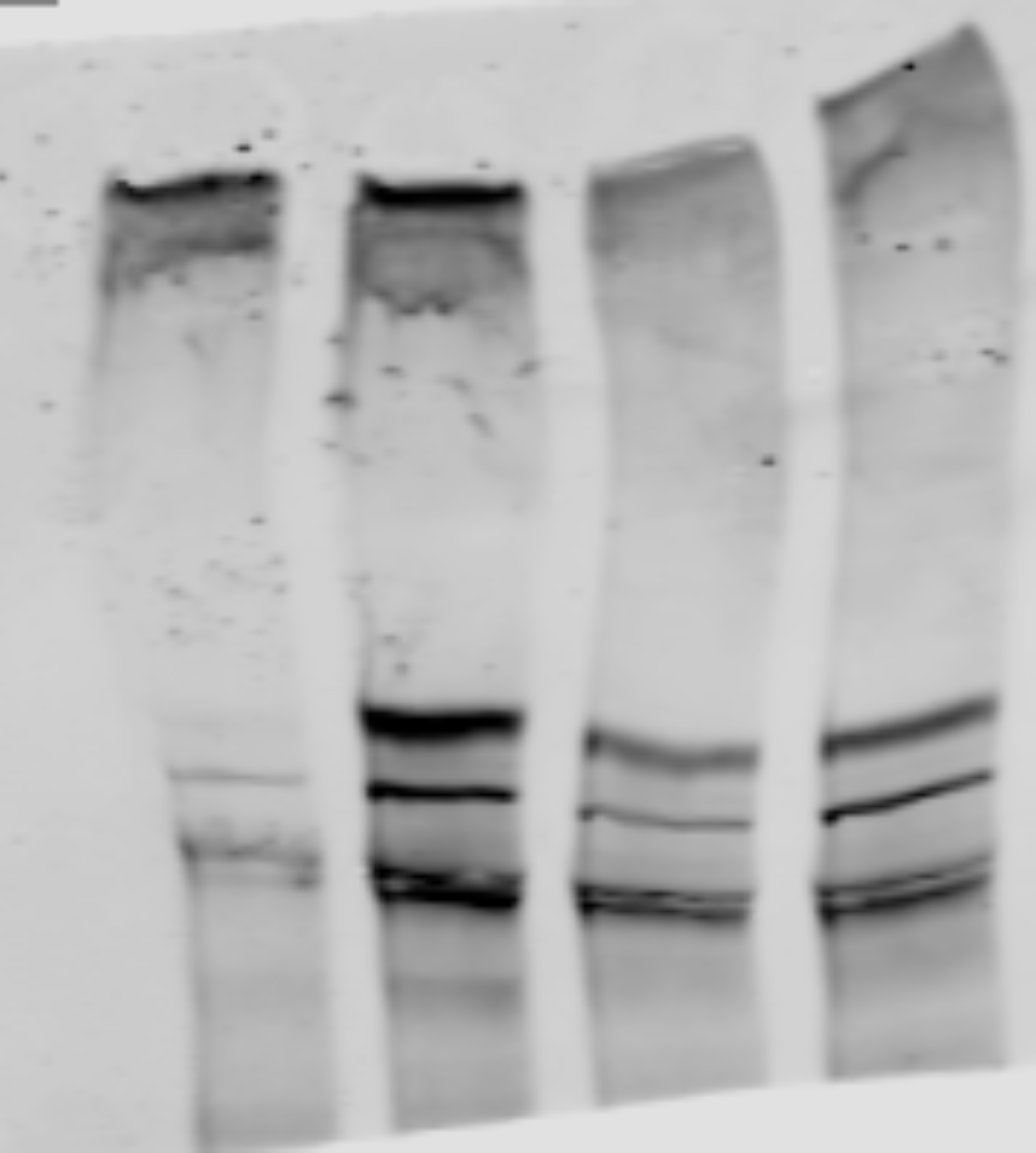

Supplement: Supplementary file 6 — Source data Fig. 5 [file 44318_2025_537_MOESM6_ESM.zip › EMBOJ-2025-120849-T_Source data Fig_5/Fig_5E/Images_Fig_5E/P-ATM_HATi.tif]

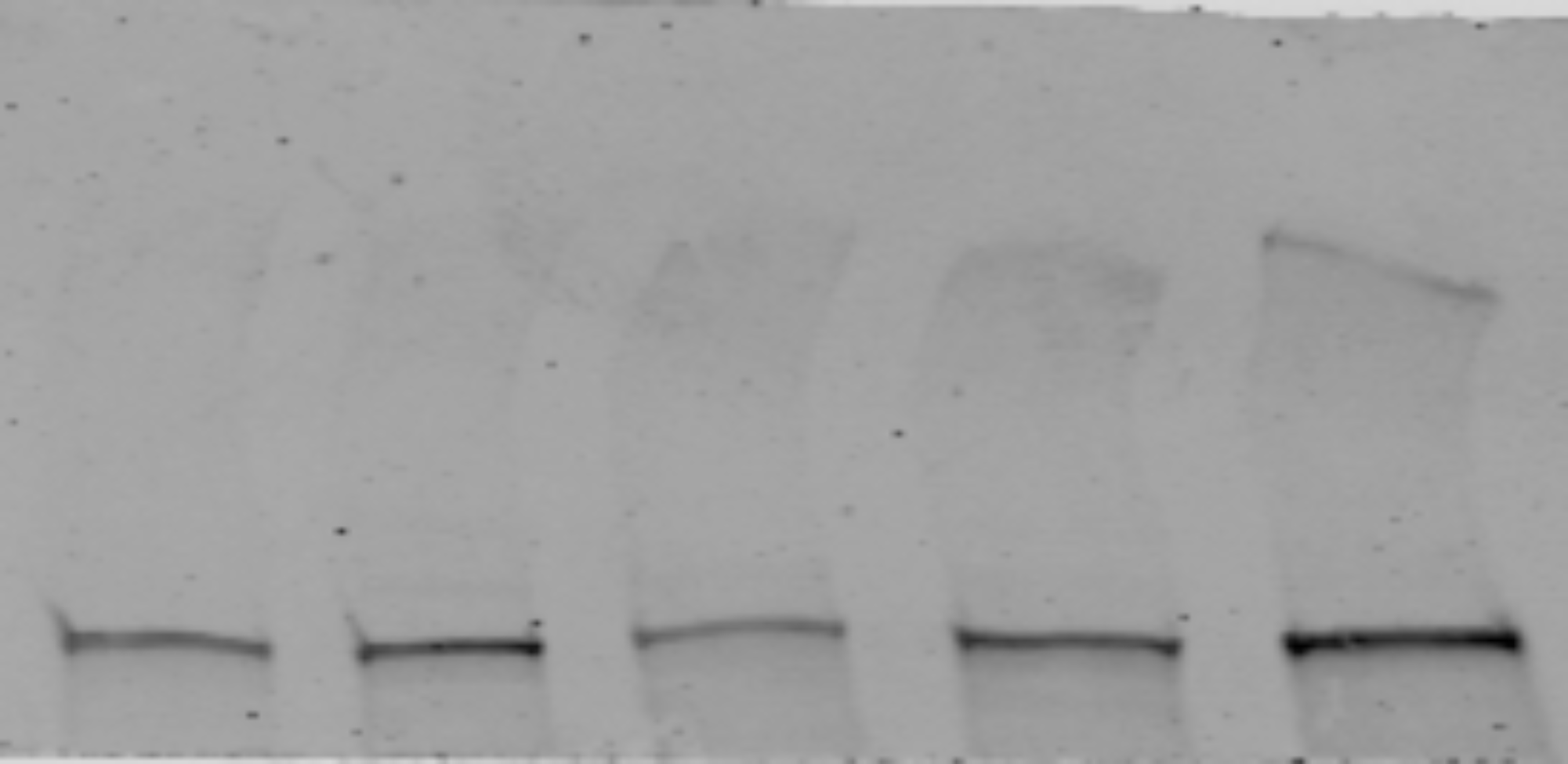

Supplement: Supplementary file 6 — Source data Fig. 5 [file 44318_2025_537_MOESM6_ESM.zip › EMBOJ-2025-120849-T_Source data Fig_5/Fig_5E/Images_Fig_5E/ATM_siHAT.tif]

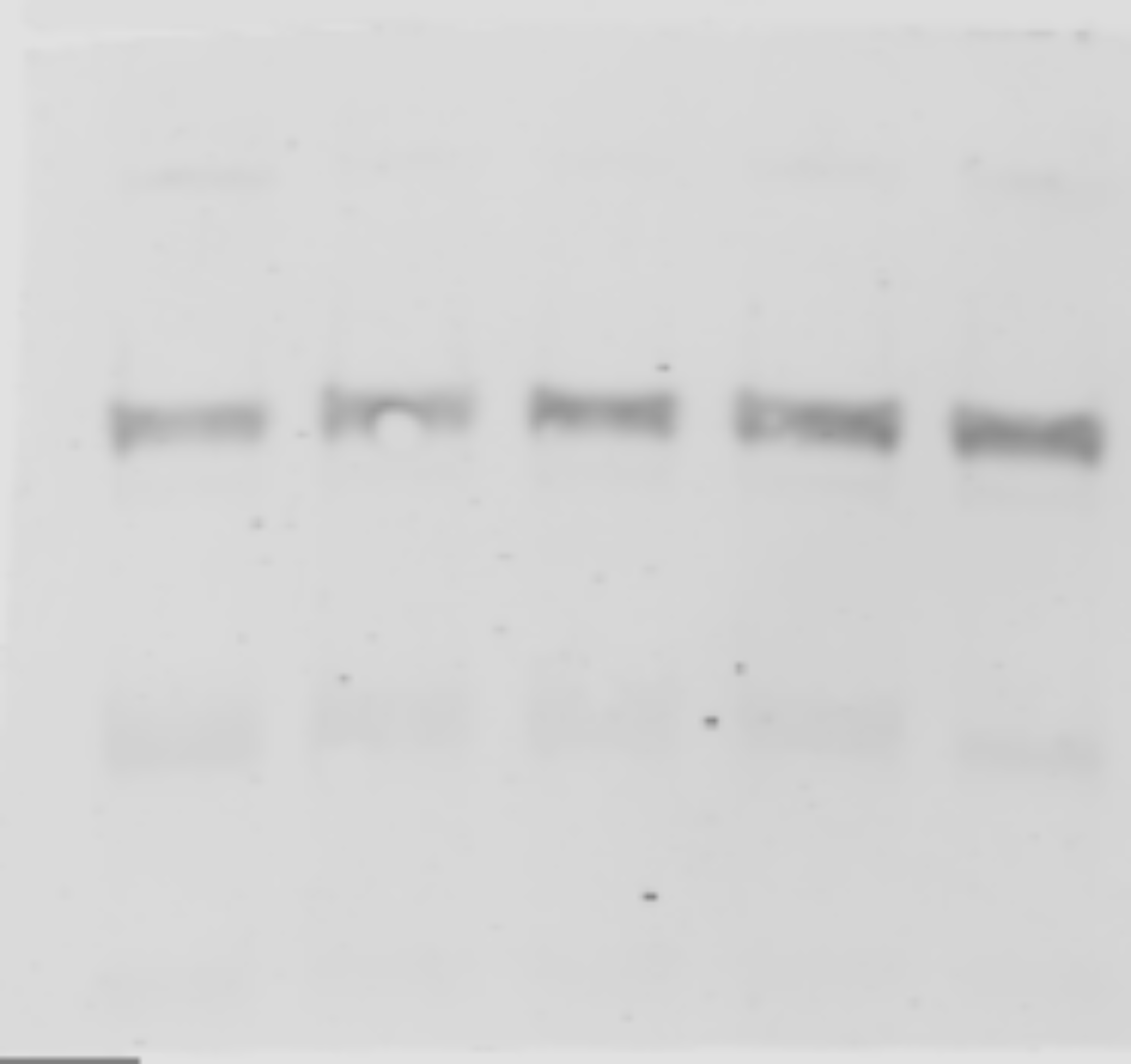

Supplement: Supplementary file 6 — Source data Fig. 5 [file 44318_2025_537_MOESM6_ESM.zip › EMBOJ-2025-120849-T_Source data Fig_5/Fig_5E/Images_Fig_5E/XAB2_siHAT.tif]

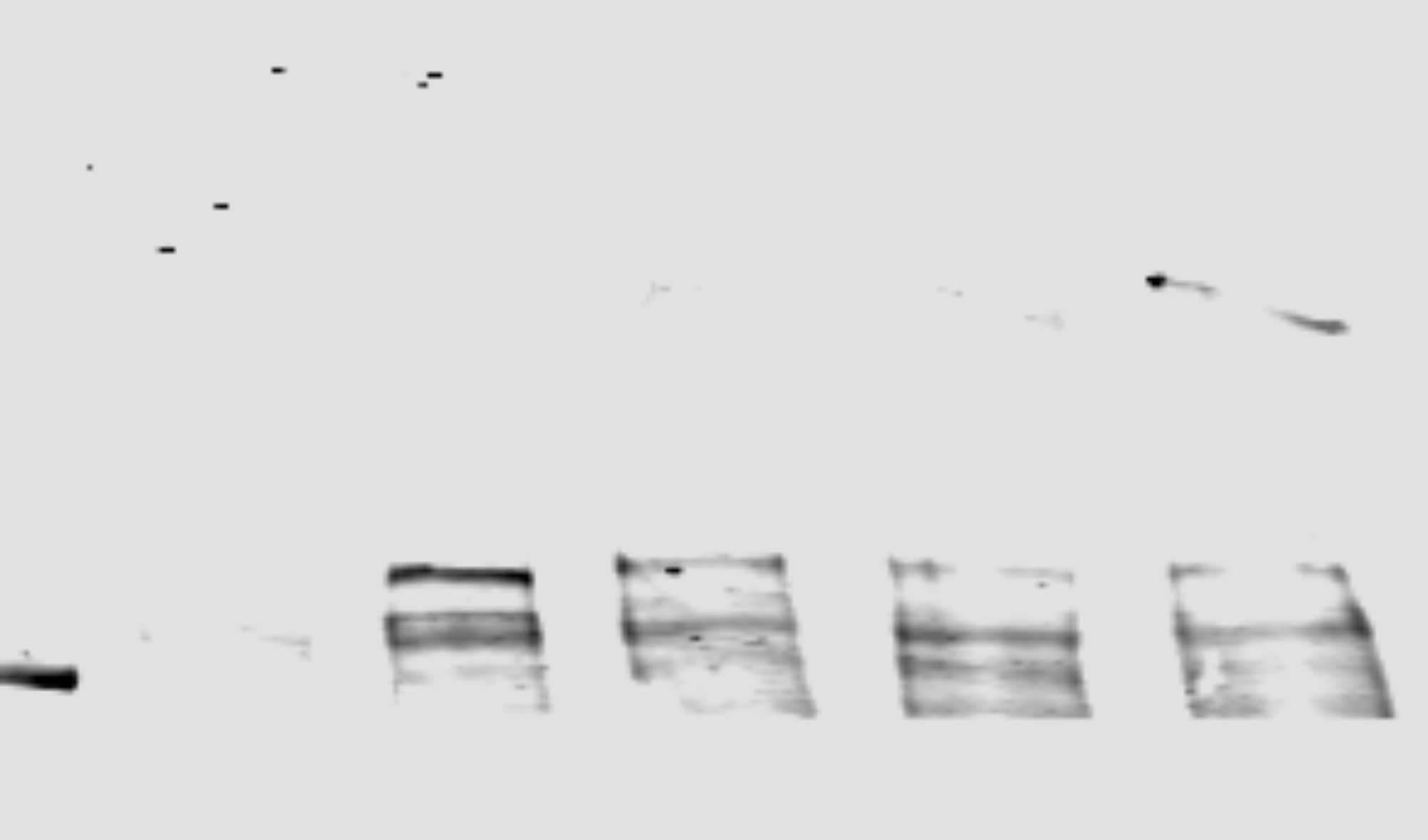

Supplement: Supplementary file 6 — Source data Fig. 5 [file 44318_2025_537_MOESM6_ESM.zip › EMBOJ-2025-120849-T_Source data Fig_5/Fig_5E/Images_Fig_5E/P-ATM_siHAT.tif]

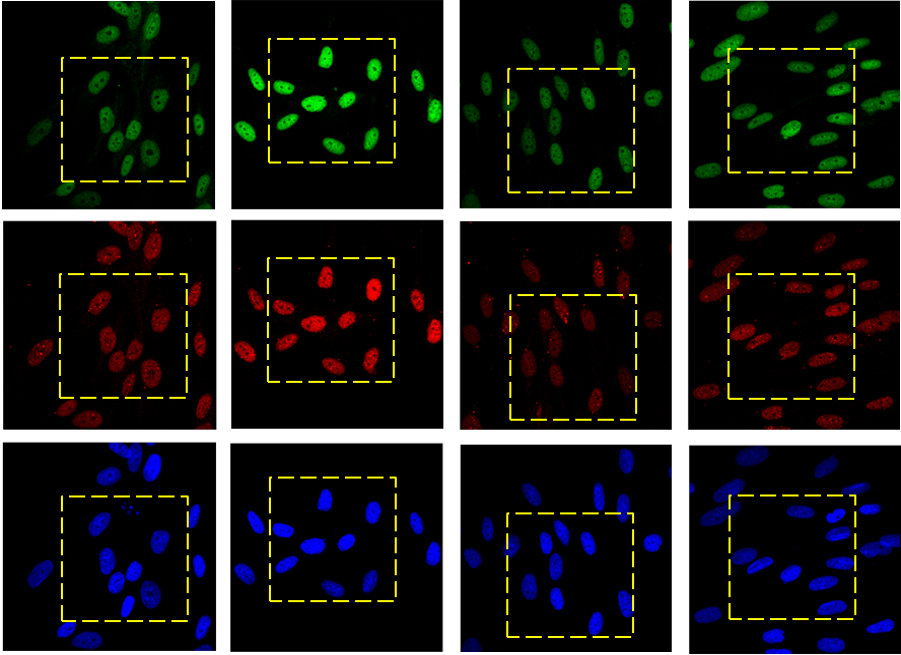

Supplement: Supplementary file 7 — Source data Fig. 6 [file 44318_2025_537_MOESM7_ESM.zip › EMBOJ-2025-120849-T_Source data Fig_6/Fig_6A/Images_Fig_6A/All_uncropped_images_Fig_6A.tif]

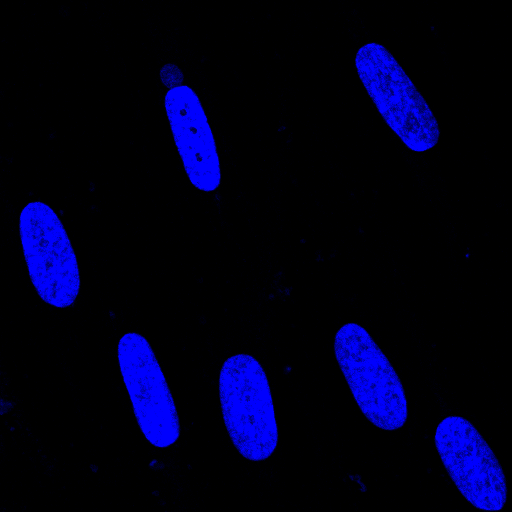

Supplement: Supplementary file 7 — Source data Fig. 6 [file 44318_2025_537_MOESM7_ESM.zip › EMBOJ-2025-120849-T_Source data Fig_6/Fig_6C/Images Fig 6C/DAPI untreated.tif]

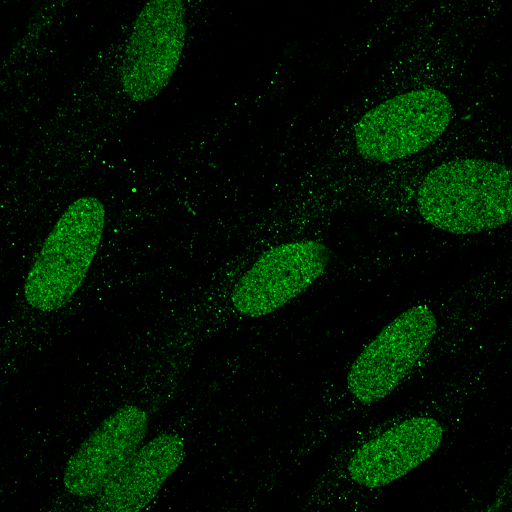

Supplement: Supplementary file 7 — Source data Fig. 6 [file 44318_2025_537_MOESM7_ESM.zip › EMBOJ-2025-120849-T_Source data Fig_6/Fig_6C/Images Fig 6C/H3S10P +UV+THZ1.tif]

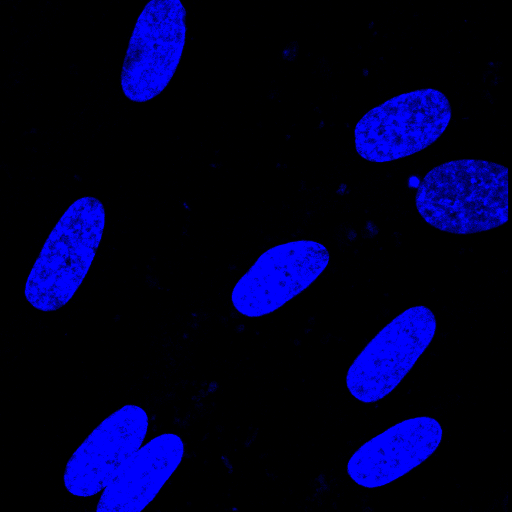

Supplement: Supplementary file 7 — Source data Fig. 6 [file 44318_2025_537_MOESM7_ESM.zip › EMBOJ-2025-120849-T_Source data Fig_6/Fig_6C/Images Fig 6C/DAPI +UV+THZ1.tif]

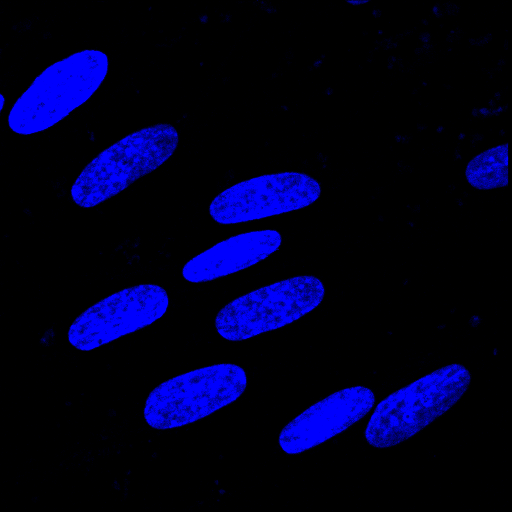

Supplement: Supplementary file 7 — Source data Fig. 6 [file 44318_2025_537_MOESM7_ESM.zip › EMBOJ-2025-120849-T_Source data Fig_6/Fig_6C/Images Fig 6C/DAPI +UV .tif]

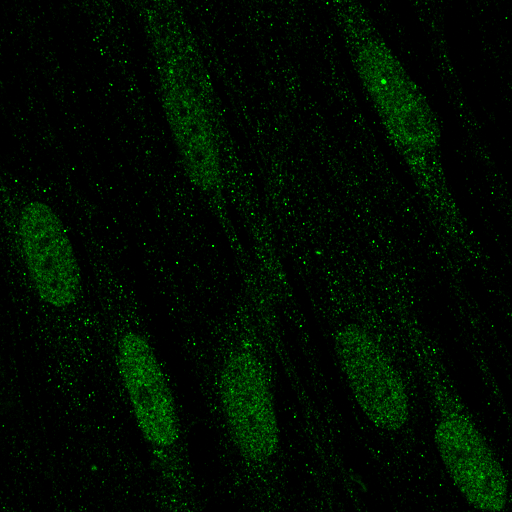

Supplement: Supplementary file 7 — Source data Fig. 6 [file 44318_2025_537_MOESM7_ESM.zip › EMBOJ-2025-120849-T_Source data Fig_6/Fig_6C/Images Fig 6C/H3S10P untr.tif]

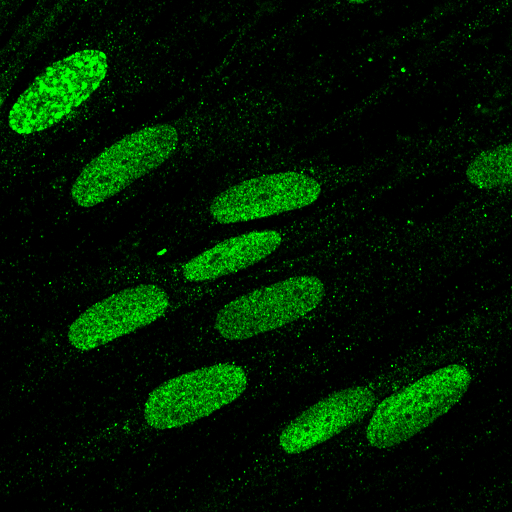

Supplement: Supplementary file 7 — Source data Fig. 6 [file 44318_2025_537_MOESM7_ESM.zip › EMBOJ-2025-120849-T_Source data Fig_6/Fig_6C/Images Fig 6C/H3S10P+UV.tif]

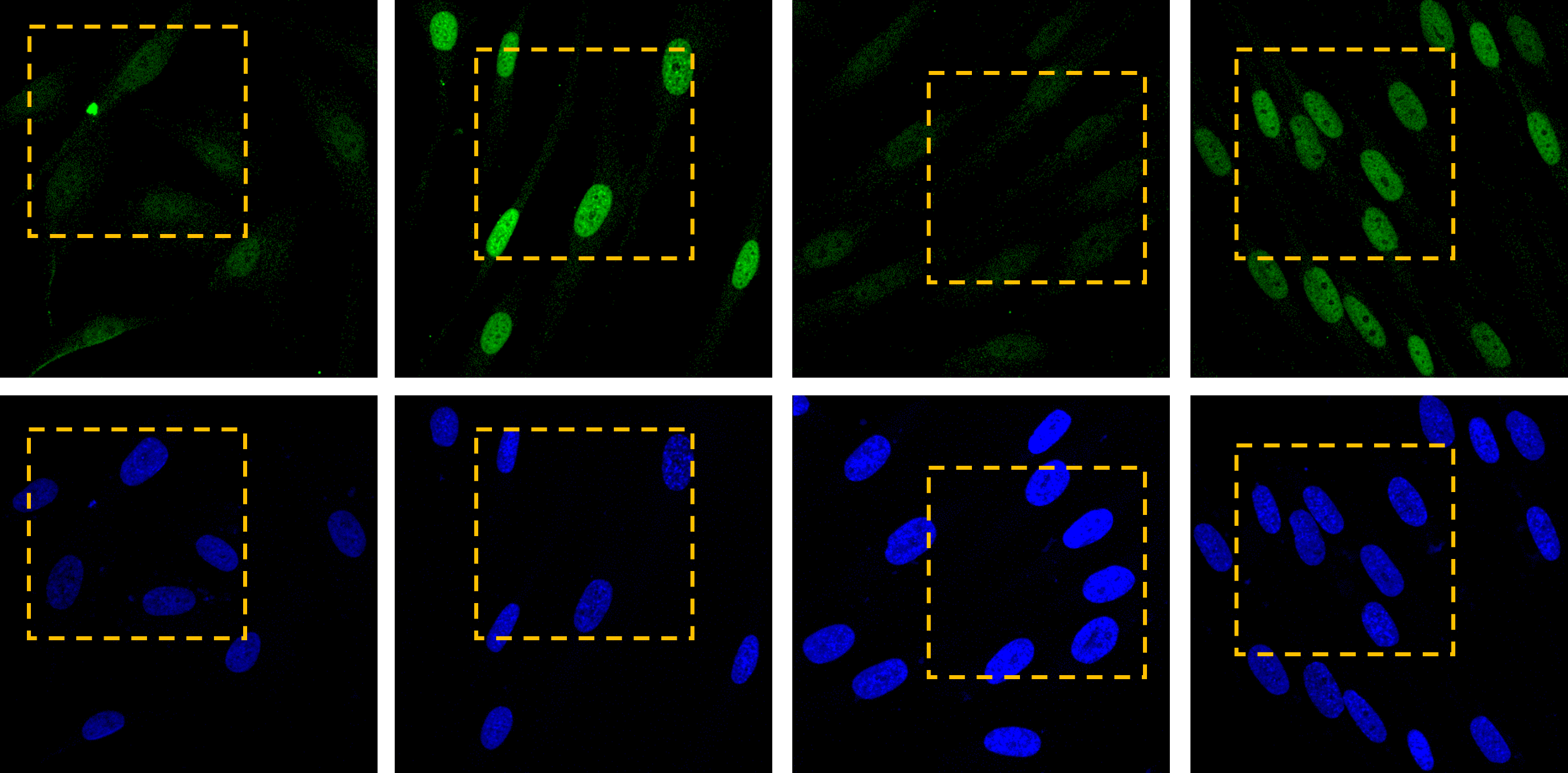

Supplement: Supplementary file 7 — Source data Fig. 6 [file 44318_2025_537_MOESM7_ESM.zip › EMBOJ-2025-120849-T_Source data Fig_6/Fig_6F/Images_Fig_6F/All_uncropped_images_Fig_6F.tif]

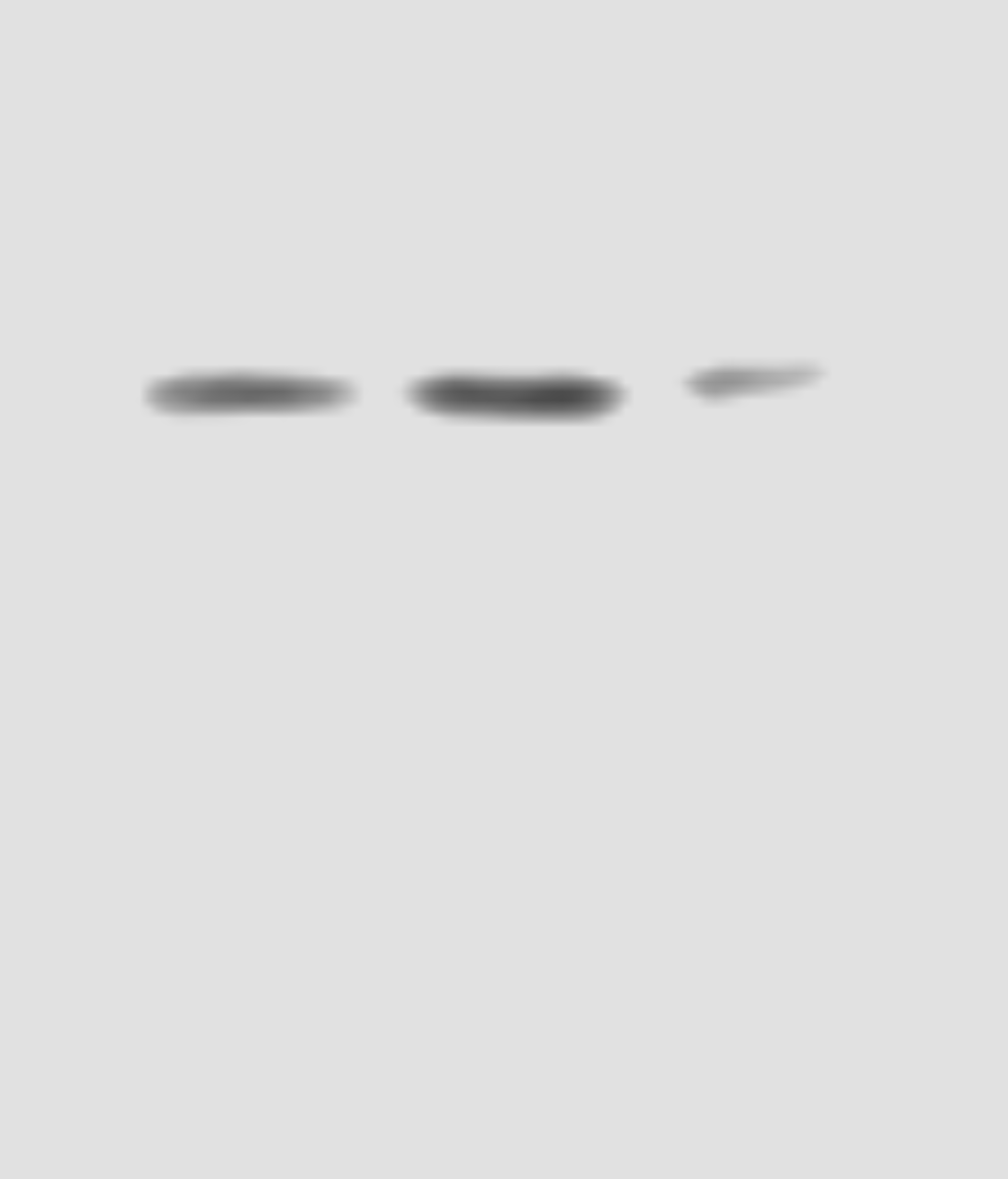

Supplement: Supplementary file 7 — Source data Fig. 6 [file 44318_2025_537_MOESM7_ESM.zip › EMBOJ-2025-120849-T_Source data Fig_6/Fig_6B/Images_Fig_6B/H3Ac.tif]

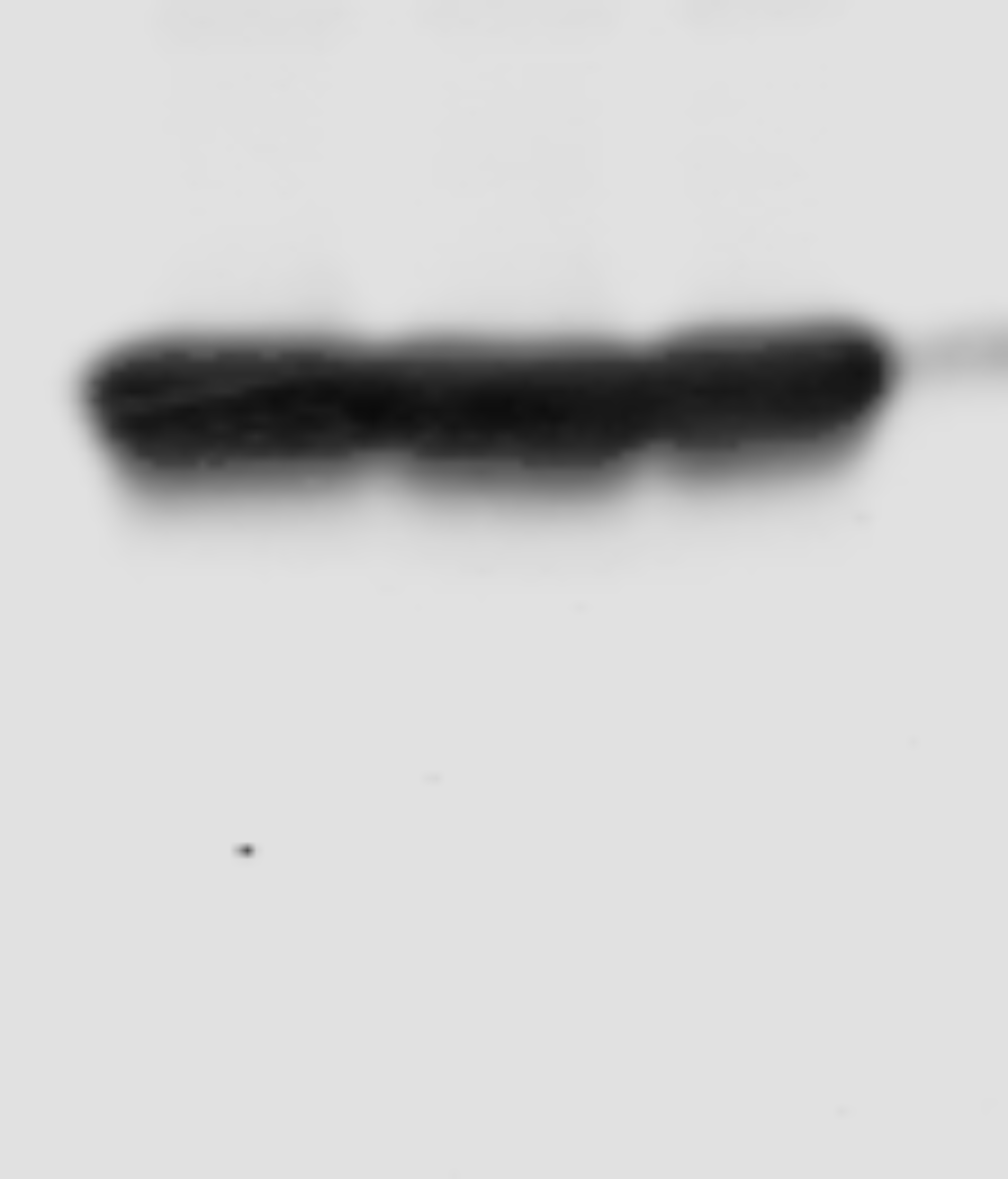

Supplement: Supplementary file 7 — Source data Fig. 6 [file 44318_2025_537_MOESM7_ESM.zip › EMBOJ-2025-120849-T_Source data Fig_6/Fig_6B/Images_Fig_6B/Histone H3.tif]

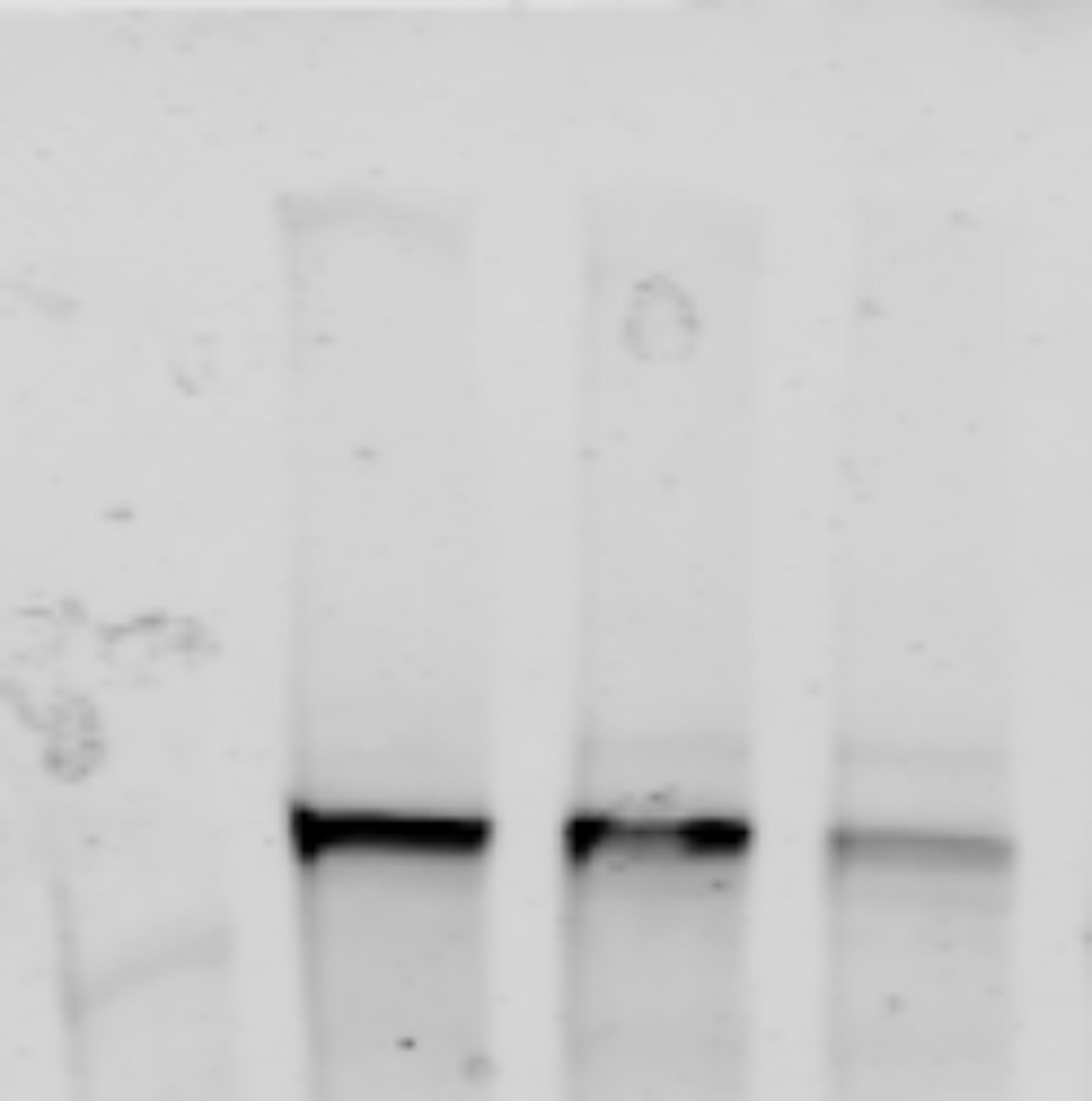

Supplement: Supplementary file 7 — Source data Fig. 6 [file 44318_2025_537_MOESM7_ESM.zip › EMBOJ-2025-120849-T_Source data Fig_6/Fig_6B/Images_Fig_6B/ATM.tif]

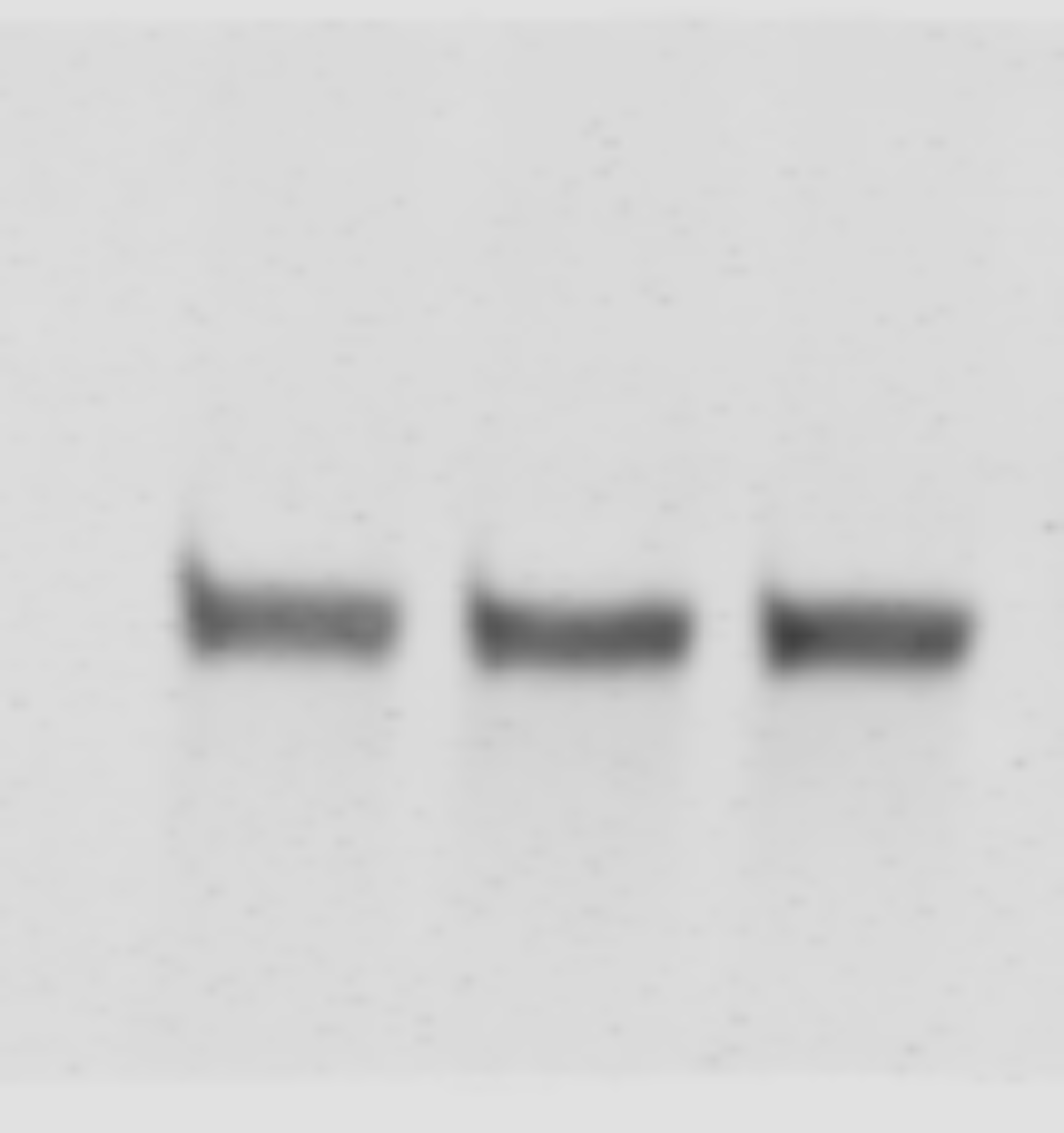

Supplement: Supplementary file 7 — Source data Fig. 6 [file 44318_2025_537_MOESM7_ESM.zip › EMBOJ-2025-120849-T_Source data Fig_6/Fig_6B/Images_Fig_6B/XAB2.tif]

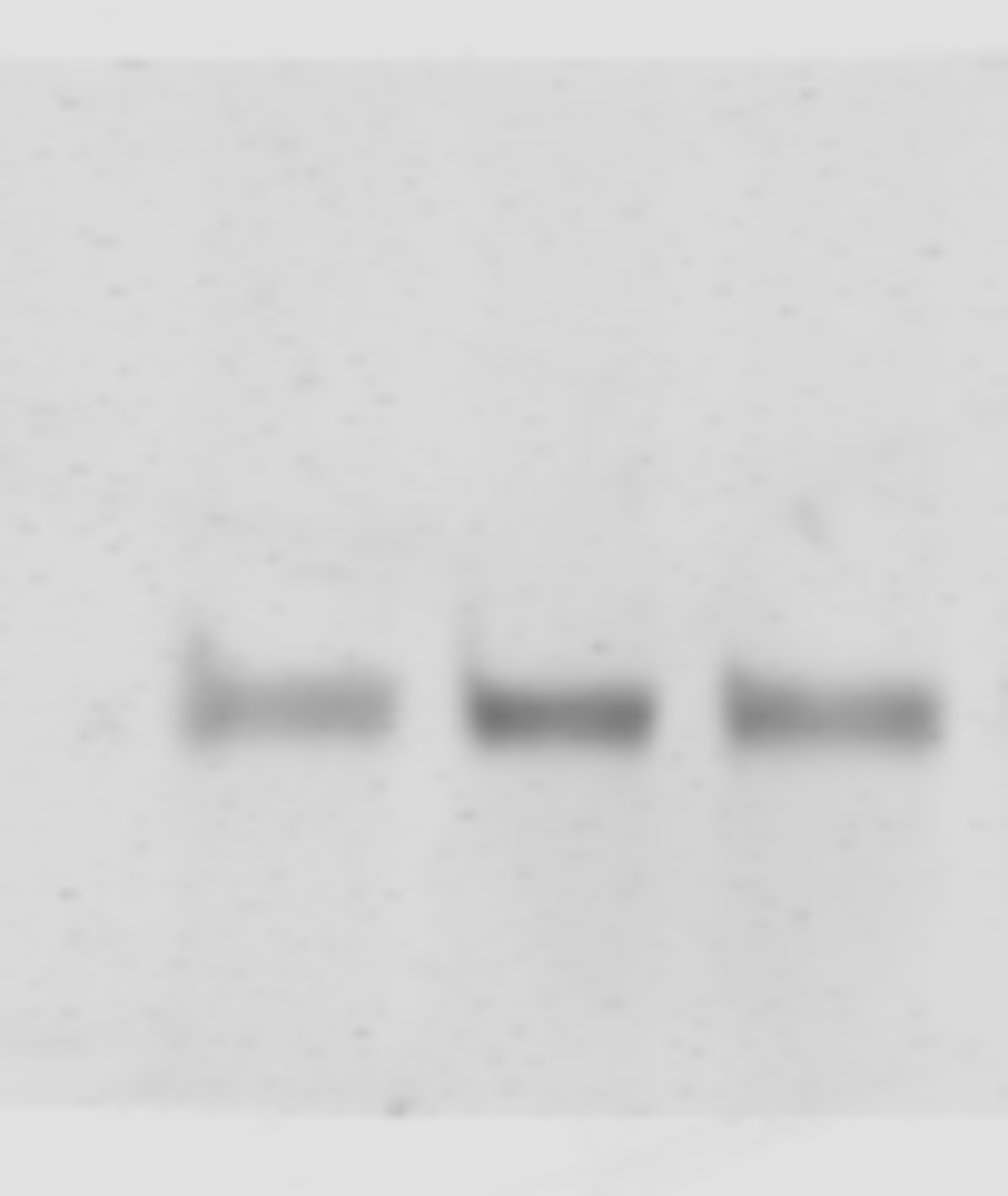

Supplement: Supplementary file 7 — Source data Fig. 6 [file 44318_2025_537_MOESM7_ESM.zip › EMBOJ-2025-120849-T_Source data Fig_6/Fig_6D/Images 6D/XAB2.tif]

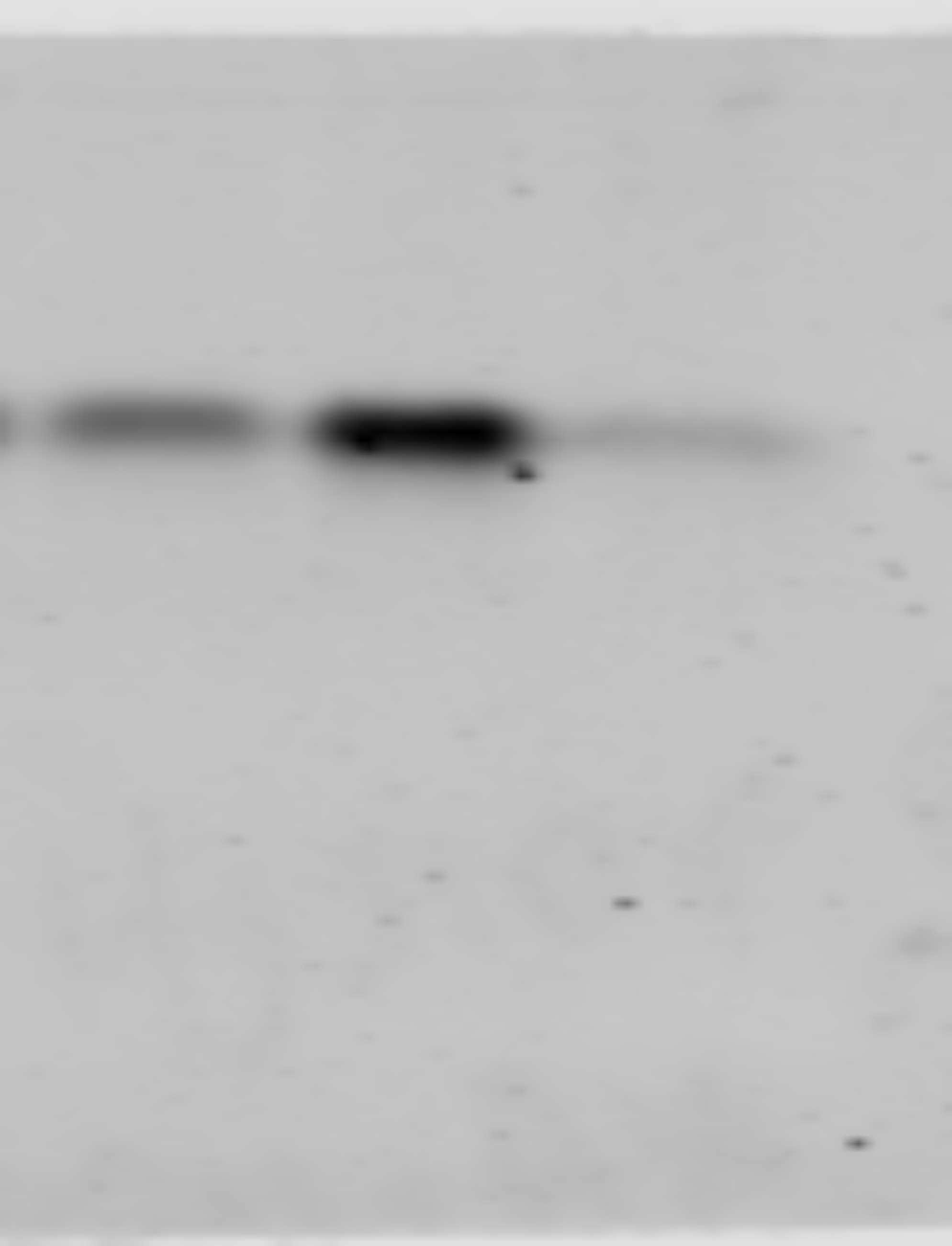

Supplement: Supplementary file 7 — Source data Fig. 6 [file 44318_2025_537_MOESM7_ESM.zip › EMBOJ-2025-120849-T_Source data Fig_6/Fig_6D/Images 6D/H3Ac.tif]

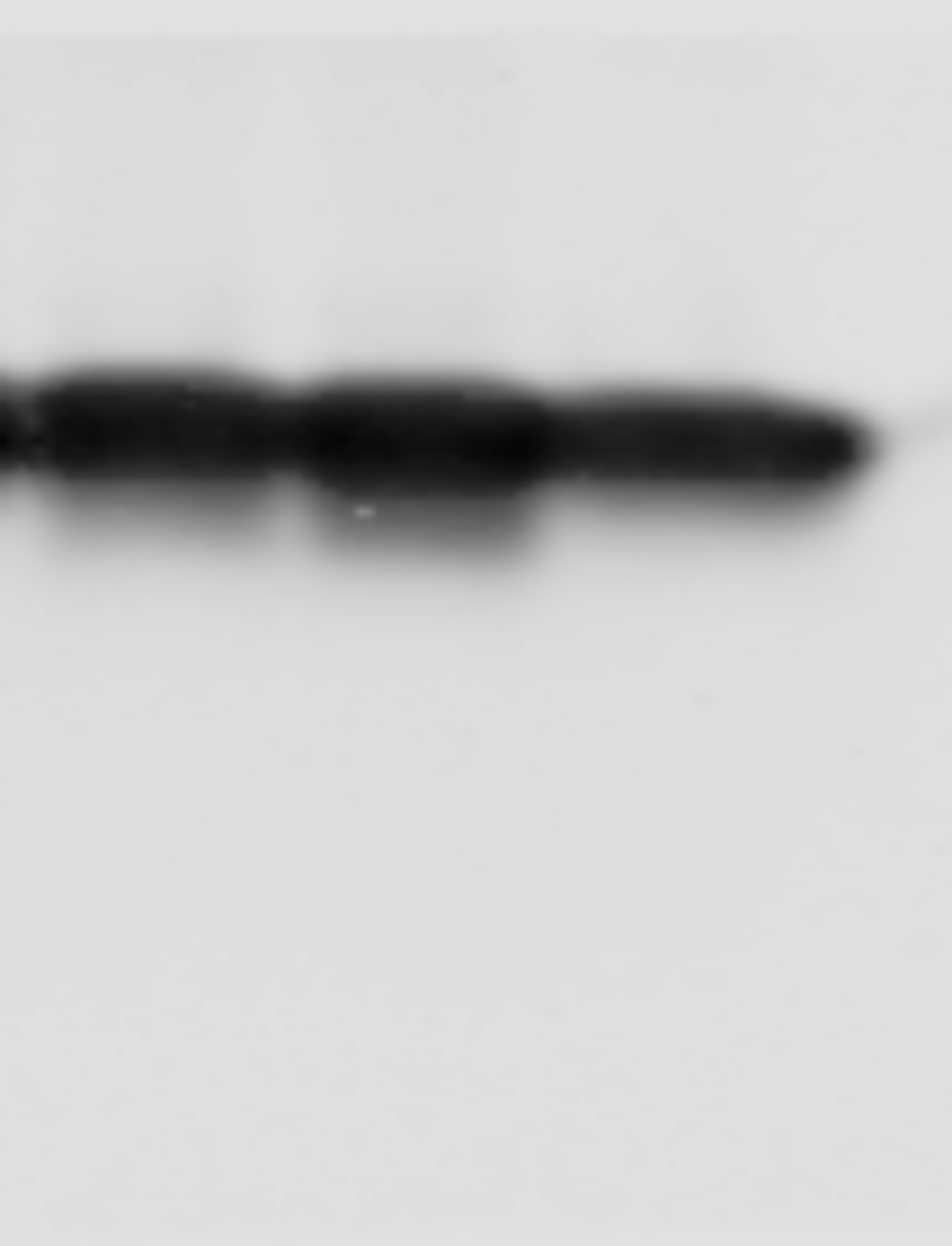

Supplement: Supplementary file 7 — Source data Fig. 6 [file 44318_2025_537_MOESM7_ESM.zip › EMBOJ-2025-120849-T_Source data Fig_6/Fig_6D/Images 6D/Histone_H3.tif]

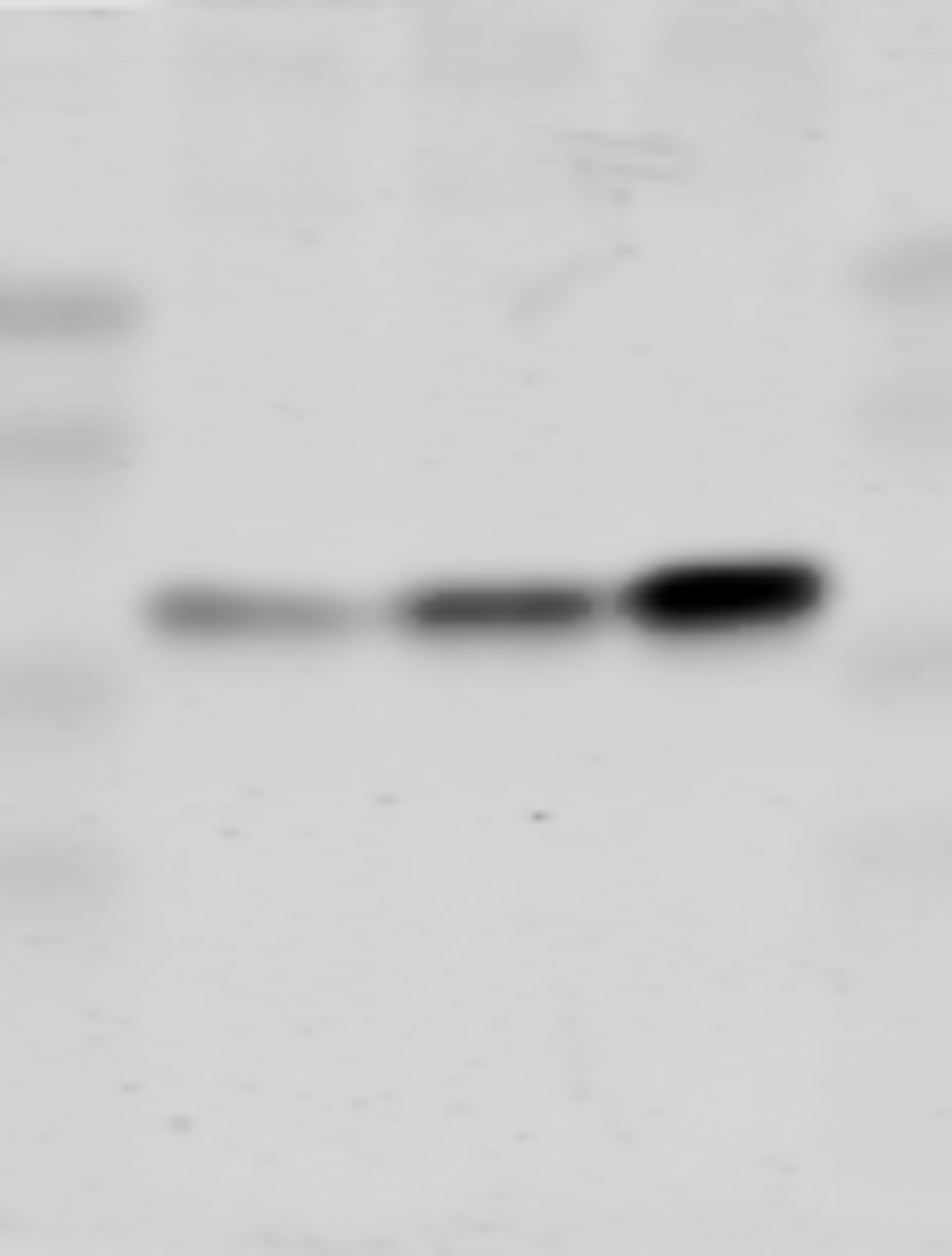

Supplement: Supplementary file 7 — Source data Fig. 6 [file 44318_2025_537_MOESM7_ESM.zip › EMBOJ-2025-120849-T_Source data Fig_6/Fig_6E/Images Fig 6E/H3Ac.tif]

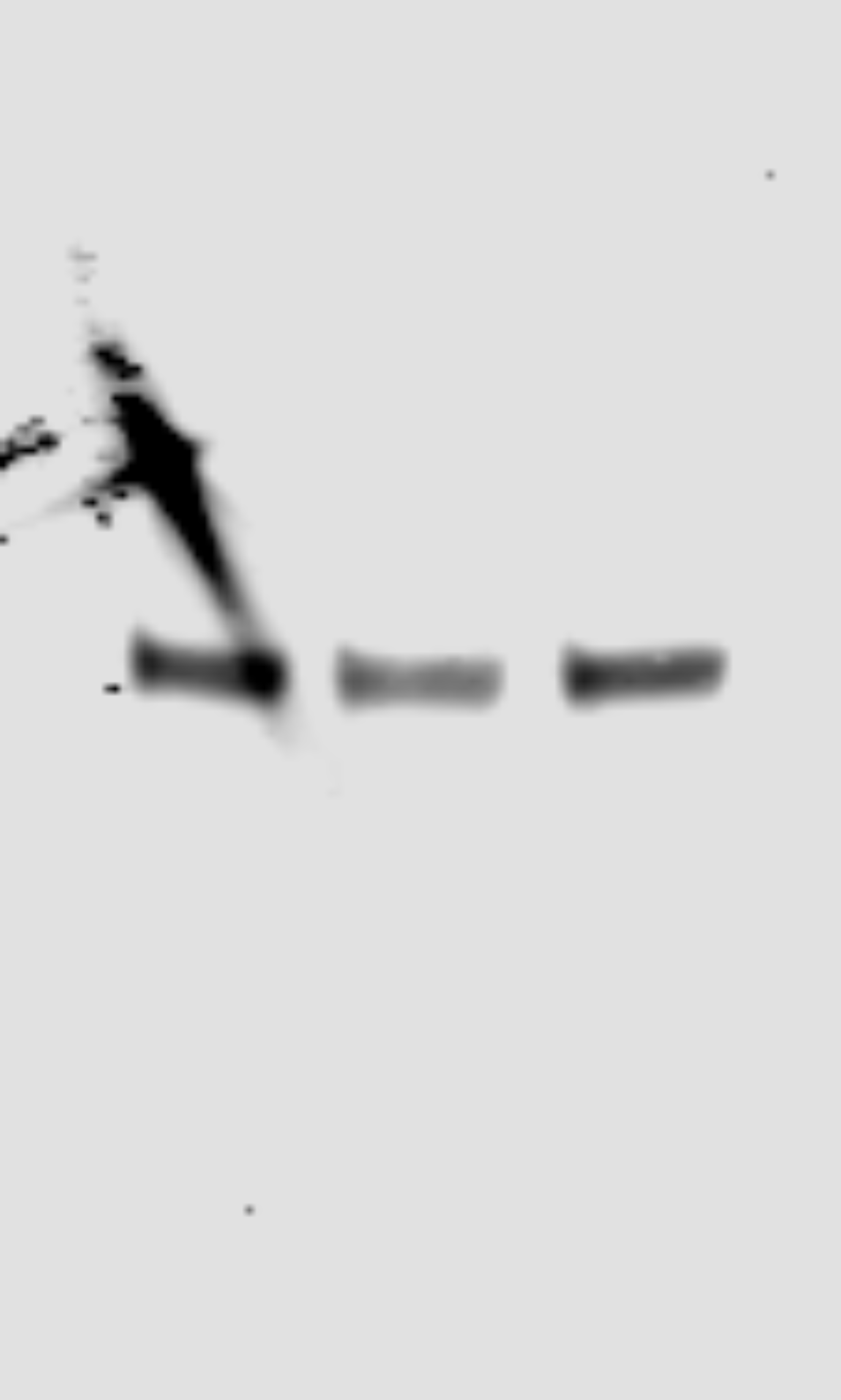

Supplement: Supplementary file 7 — Source data Fig. 6 [file 44318_2025_537_MOESM7_ESM.zip › EMBOJ-2025-120849-T_Source data Fig_6/Fig_6E/Images Fig 6E/XAB2.tif]

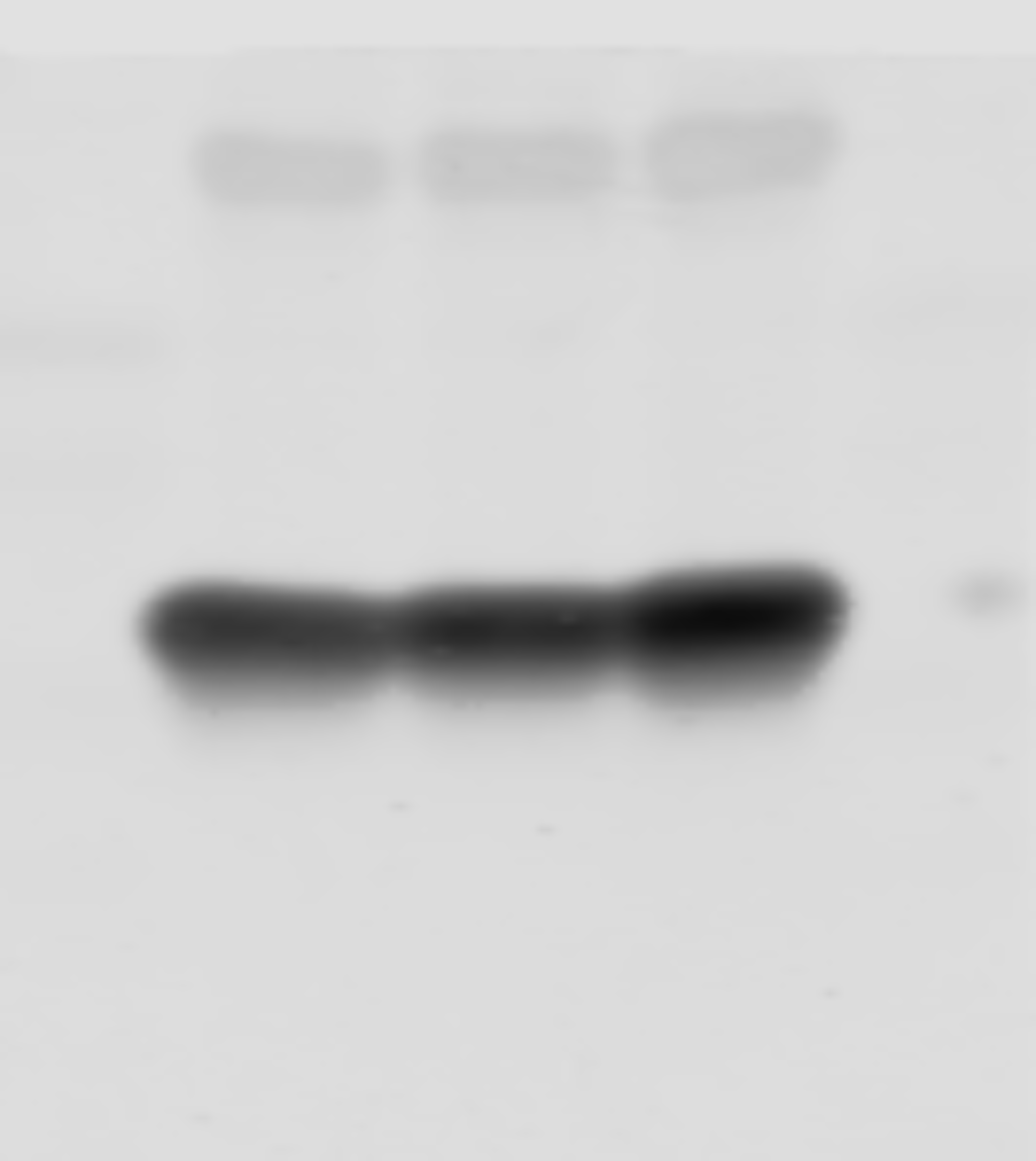

Supplement: Supplementary file 7 — Source data Fig. 6 [file 44318_2025_537_MOESM7_ESM.zip › EMBOJ-2025-120849-T_Source data Fig_6/Fig_6E/Images Fig 6E/Histone_H3.tif]

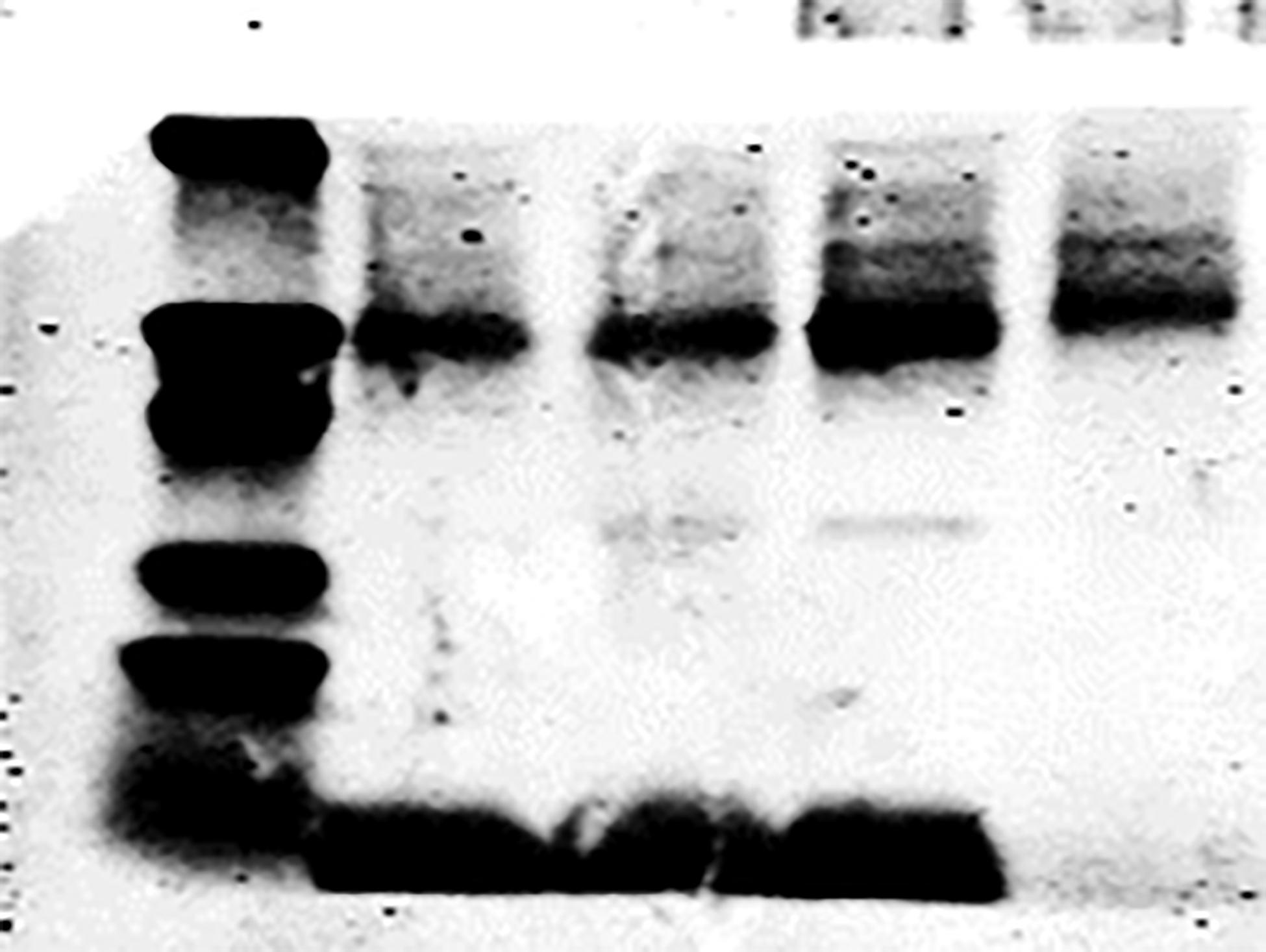

Supplement: Supplementary file 7 — Source data Fig. 6 [file 44318_2025_537_MOESM7_ESM.zip › EMBOJ-2025-120849-T_Source data Fig_6/Fig_6G/Images_Fig_6G/IP_S9.6_IB_H3S10P_Fig_6G.tif]

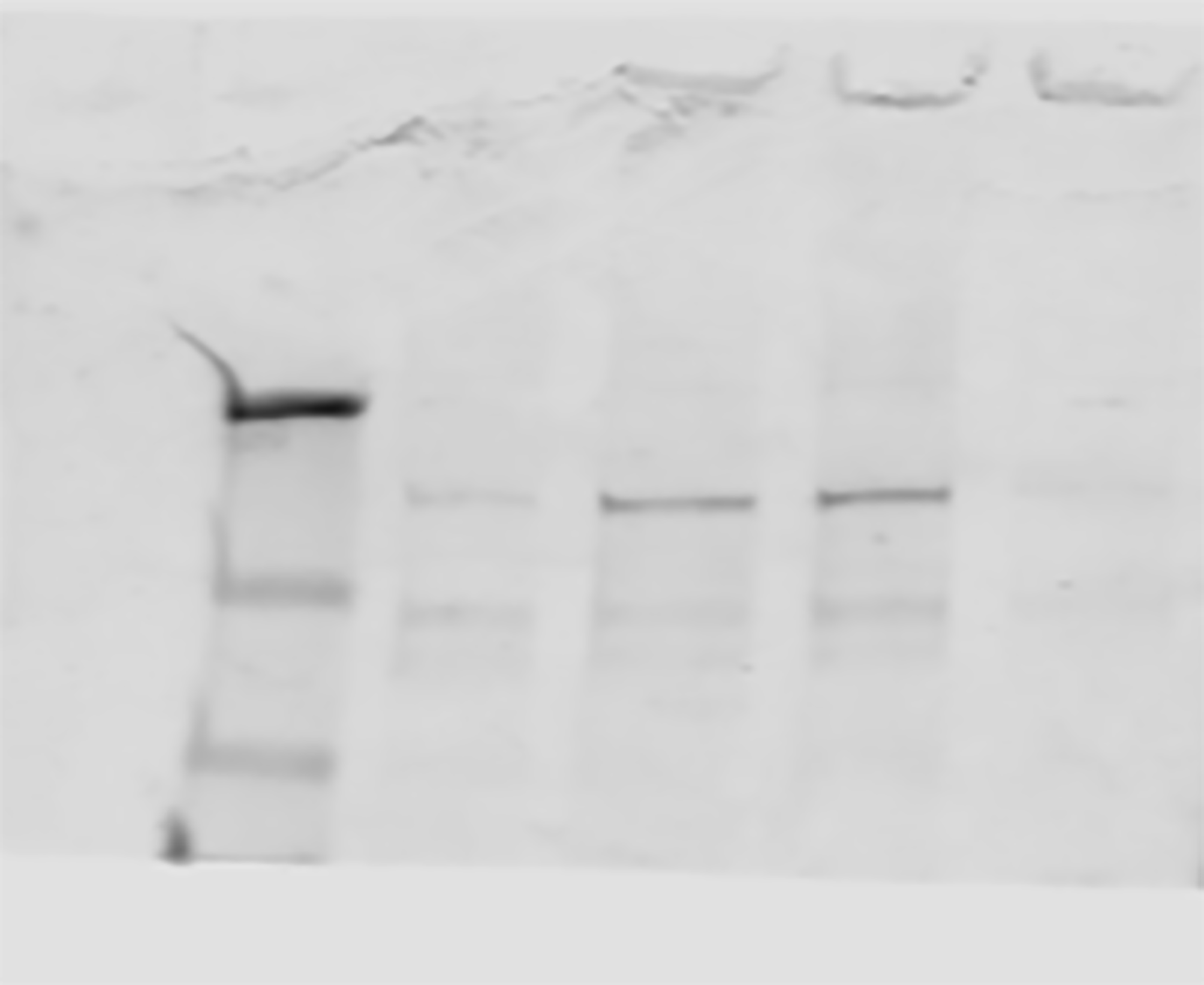

Supplement: Supplementary file 7 — Source data Fig. 6 [file 44318_2025_537_MOESM7_ESM.zip › EMBOJ-2025-120849-T_Source data Fig_6/Fig_6G/Images_Fig_6G/IP_S9.6_IB_CSB_Fig_6G.tif]

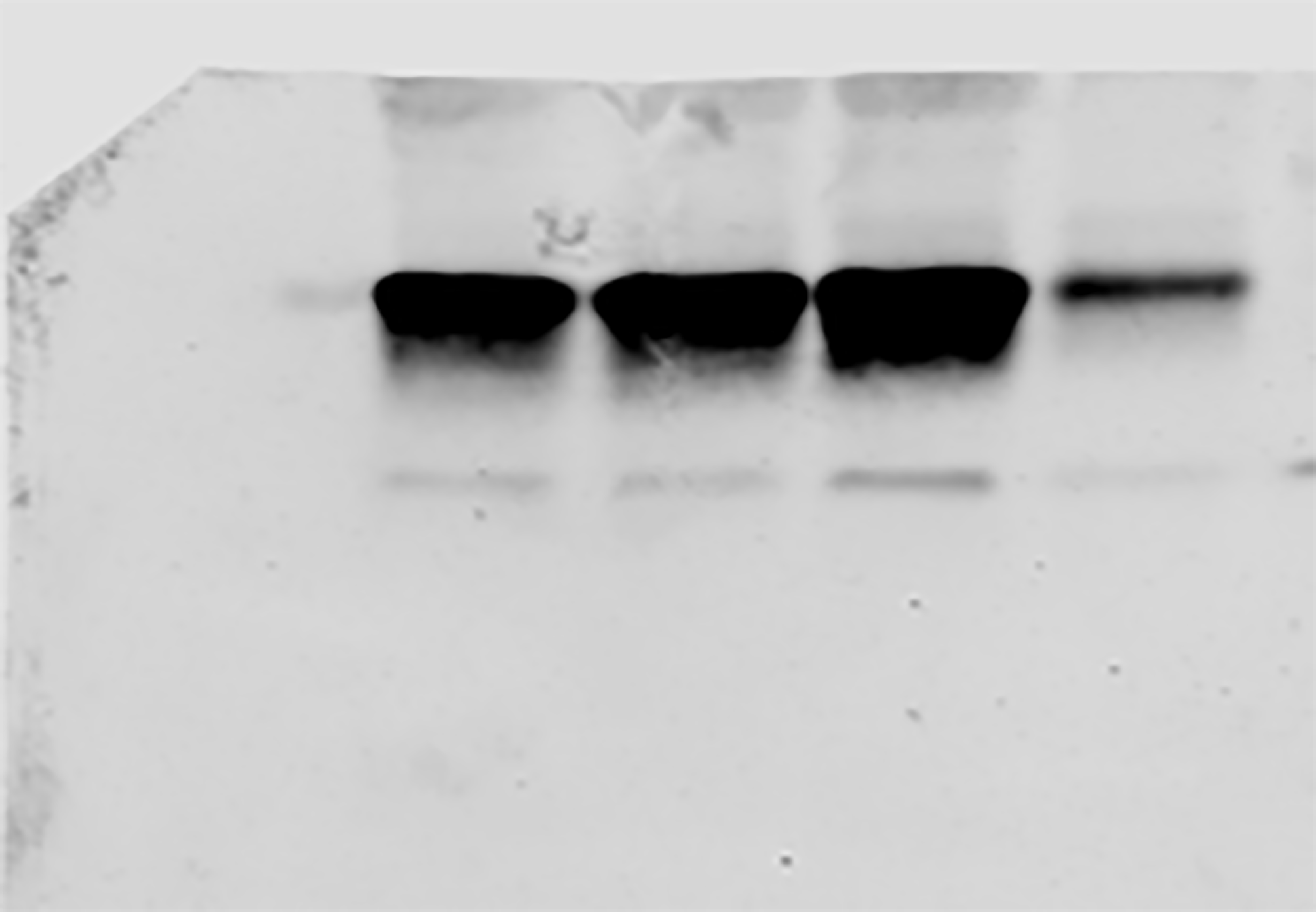

Supplement: Supplementary file 7 — Source data Fig. 6 [file 44318_2025_537_MOESM7_ESM.zip › EMBOJ-2025-120849-T_Source data Fig_6/Fig_6G/Images_Fig_6G/IP_S9.6_IB_Histone_H3_Fig_6G.tif]
