# Supplementary material for: Crosstalk between chromatin state and ATM signalling in DNA damage-induced transcription stress
Source: EMBO J. 2025 Aug 26;44(19):5564–94. doi: 10.1038/s44318-025-00537-7 (PMC12489091; doi:10.1038/s44318-025-00537-7)
Supplement: Supplementary file 6 — Source data Fig. 5 [file 44318_2025_537_MOESM6_ESM.zip › EMBOJ-2025-120849-T_Source data Fig_5/Fig_5A/readme_Fig_5A.docx]

**RNAseH1 recruitment to UV-C laser–irradiated subnuclear regions (Figure 5A)**

**File Description:**
The Excel file contains the numerical data corresponding to the analysis presented in Figure 5A of the manuscript.

**Experimental Details:**
Recruitment of GFP-tagged RNAseH1 (D145N) to UV-C laser microirradiation sites in live-cells was monitored on a Leica TCS SP5 AOBS laser scanning confocal microscope.

**Data Acquisition and Quantification:**
Images were acquired and quantified using LASAF software. Quantification was performed in Microsoft Excel.

For each time point and individual cell, the data represent:
*(Fluorescence intensity in the UV-C–irradiated region − nuclear background fluorescence), normalized to pre-irradiation fluorescence levels.*

**Image Processing:**
All quantifications were performed on unmodified, raw images. No image processing or resolution downsampling was applied post-acquisition.

**Graphing and Statistical Analysis:**
Graphs were generated in GraphPad Prism. Statistical analyses were also performed using Prism, as described in the accompanying Excel file.
