# Supplementary material for: Crosstalk between chromatin state and ATM signalling in DNA damage-induced transcription stress
Source: EMBO J. 2025 Aug 26;44(19):5564–94. doi: 10.1038/s44318-025-00537-7 (PMC12489091; doi:10.1038/s44318-025-00537-7)
Supplement: Supplementary file 6 — Source data Fig. 5 [file 44318_2025_537_MOESM6_ESM.zip › EMBOJ-2025-120849-T_Source data Fig_5/Fig_5E/readme_Fig_5E.docx]

**Immunoblots showing ATM phosphorylation following HAT inhibition or depletion (Figure 5E)**

**Folder Contents:**
The folder contains source data for the immunoblots presented in Figure 5E of the manuscript, including images (“Images” subfolder) and quantification (Excel file).

**Image Acquisition and Processing:**

- Immunoblot images were acquired using an Odyssey CLx (*LI-COR)* imaging system.
- Image intensity levels were adjusted prior to quantification to ensure grayscale rendering and avoid saturation. These adjustments were applied uniformly across the membrane and did not alter the relative signal intensities.
- Images were exported as TIFFs directly from the *LI-COR* Image Studio 6.0 Software.

**Blotting and Antibody Incubation Details:**

- Membranes were cut prior to antibody incubation to allow separate hybridization with different antibodies.
- Phosphorylated and total ATM were detected on the same membrane probed sequentially.

**Quantification and Analysis:**

- Signal intensities were measured using ImageQuant software.
- Phospho-ATM values were normalized to ATM levels and expressed as fold change relative to non-irradiated controls.
- The accompanying Excel file contains:
  - Raw and normalized quantification data
  - Cropped versions of the blot images corresponding to the final figure panel (included for reference only)
  - Uncropped images
