# Supplementary material for: Crosstalk between chromatin state and ATM signalling in DNA damage-induced transcription stress
Source: EMBO J. 2025 Aug 26;44(19):5564–94. doi: 10.1038/s44318-025-00537-7 (PMC12489091; doi:10.1038/s44318-025-00537-7)
Supplement: Supplementary file 8 — Source data Fig. 7 [file 44318_2025_537_MOESM8_ESM.zip › EMBOJ-2025-120849-T_Source data Fig_7/Fig_7A/readme_Fig_7A.docx]

**Immunofluorescence detection of UV-induced p38 MAPK activating phosphorylation in control and ATM depleted cells (Figure 7A)**

The folder contains microscopy images (“images” subfolder) and quantitative data (Excel file) corresponding to the IF analysis presented in Figure 7A of the manuscript.

**Image Acquisition:**

- Confocal images were acquired using a Zeiss LSM700 laser-scanning confocal microscope at a resolution of 512 × 512 pixels.
- Acquisition settings were optimized to enable high-throughput imaging across multiple treatment conditions.
- Image files (Zeiss “lsm” files) were exported as TIFF files from Fiji (ImageJ) without any modifications.

**Quantification and Analysis:**

- IF signal intensities were quantified from multiple unmodified images using Fiji (ImageJ), and the data were normalized to control samples in Excel.
- No post-acquisition image processing (e.g., filtering, downsampling, or cropping) was applied to the data used for analysis.
- Signal intensities were normalized to the average of non-irradiated cells, and graphs were generated in GraphPad Prism.
- Statistical analyses were also performed in Prism, as detailed in the accompanying Excel file.

**Figure Presentation Notes:**

- Brightness and contrast adjustments were applied only to the representative images shown in the figure panel, for improved visualization.
- These adjustments were applied uniformly across all experimental conditions.
- All quantifications were based on the original, unmodified images.
