# Supplementary material for: Crosstalk between chromatin state and ATM signalling in DNA damage-induced transcription stress
Source: EMBO J. 2025 Aug 26;44(19):5564–94. doi: 10.1038/s44318-025-00537-7 (PMC12489091; doi:10.1038/s44318-025-00537-7)
Supplement: Supplementary file 8 — Source data Fig. 7 [file 44318_2025_537_MOESM8_ESM.zip › EMBOJ-2025-120849-T_Source data Fig_7/Fig_7C/readme_Fig_7C.docx]

**Cell Viability of UV irradiated cells (Figure 7C)**

This Excel file contains the source data for Figure 7C of the manuscript.

Cell viability was assessed 48 hours after UV irradiation using the AlamarBlue™ assay. Fluorescence was measured 3 hours after reagent addition at 570 nm using a Promega Glomax® Multimode reader.

Data represent mean fluorescence intensities (± SEM), normalized to untreated, non-irradiated controls. Values were averaged from technical triplicates and derived from four independent experiments (ATM inhibitor) or three (p38 and MSK1 inhibitors).
