# Supplementary material for: Crosstalk between chromatin state and ATM signalling in DNA damage-induced transcription stress
Source: EMBO J. 2025 Aug 26;44(19):5564–94. doi: 10.1038/s44318-025-00537-7 (PMC12489091; doi:10.1038/s44318-025-00537-7)
Supplement: Supplementary file 9 — Expanded View Figures [file 44318_2025_537_MOESM9_ESM.pdf]

Expanded View Figures

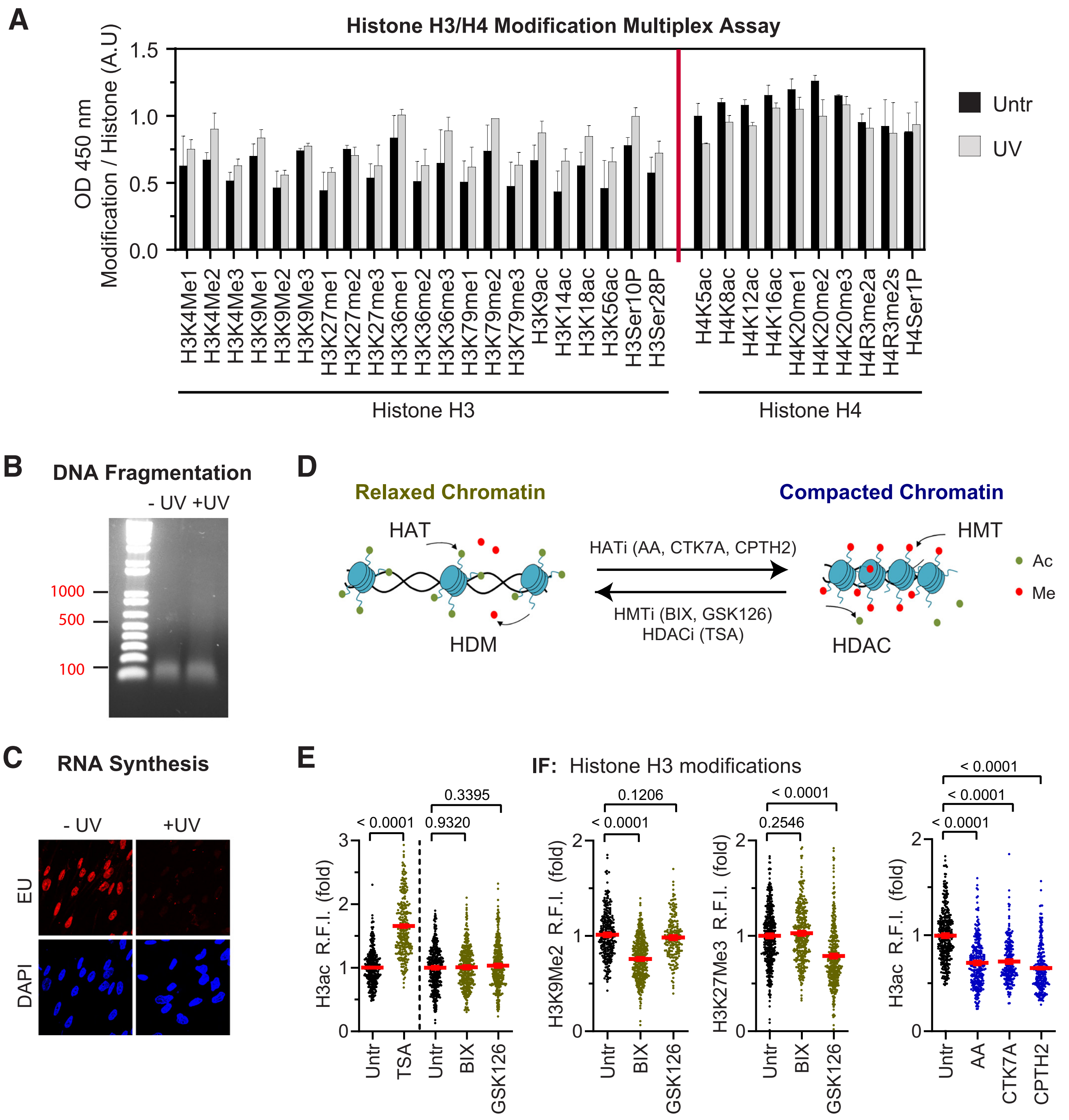

 © The Author(s)

Figure EV1. UV-induced chromatin remodeling and its pharmacological modulation.

(**A**) Detection of UV-induced changes in histone H3 and H4 post-translational modifications (PTMs). Histones were extracted from quiescent HDFs, either untreated or UV-irradiated (40 J/m^2, 2 h). Thirty-one well-characterized histone H3 or H4 PTMs were measured using a multiplex ELISA-based colorimetric assay. Optical density (OD_{450} nm) values for each modification were normalized to total histone H3 and H4 levels. Bars represent mean normalized OD_{450} values from two independent biological replicates, each performed in duplicate. (**B**) Quality control of chromatin fragmentation used for chromatin immunoprecipitations (Fig. 1C). Agarose gel of DNA purified from cross-linked, digested chromatin is shown. DNA ladder: 1 Kb Plus (Invitrogen). (**C**) UV-induced transcription suppression. Representative immunofluorescence images of Ethynyl-uridine (EU) incorporation into nascent RNA in mock-treated or UV-irradiated (20 J/m^2, 2 h) quiescent HDFs. Nuclei were stained with DAPI. (**D**) Schematic illustrating the chromatin-modifying effects of pharmacological inhibitors used in this study. Chromatin relaxation is induced by the broad-spectrum histone deacetylase (HDAC) inhibitor Trichostatin A (TSA), which stimulates histone hyperacetylation, and by histone methyltransferase (HMT) inhibitors BIX01294 and GSK126, which prevent deposition of the suppressive marks H3K9me2 and H3K27me3, respectively. Chromatin compaction is promoted by histone acetyltransferase (HAT) inhibitors Anacardic Acid (AA), CTK7A (PCAF/p300 inhibitor), and CPTH2 (GCN5 inhibitor). (**E**) Effects of chromatin-modifying drugs on histone marks associated with relaxed or compacted chromatin. Quiescent HDFs were treated with the indicated inhibitors, followed by immunofluorescence detection of H3-specific PTMs in detergent-extracted nuclei. Acetylation changes were assessed using an antibody recognizing H3 acetylation at lysines 9, 14, 18, 23, and 27. H3K9me2 and H3K27me3 levels were detected following treatment with BIX01294 or GSK126, respectively. Nuclei were stained with DAPI. Plotted are mean fluorescence intensities normalized to untreated controls. Data information: (**E**) Mean ± SEM of (left to right): $n = 356/283/431/363/3949$ (three biological replicates), 124/98/93 (two replicates), 445/341/396 (three replicates), and 358/278/240/261 (three replicates). Unpaired two-tailed *t*-test with Welch's correction (H3ac by TSA), or Brown–Forsythe and Welch's ANOVA with Games–Howell's multiple comparisons test.

© The Author(s)

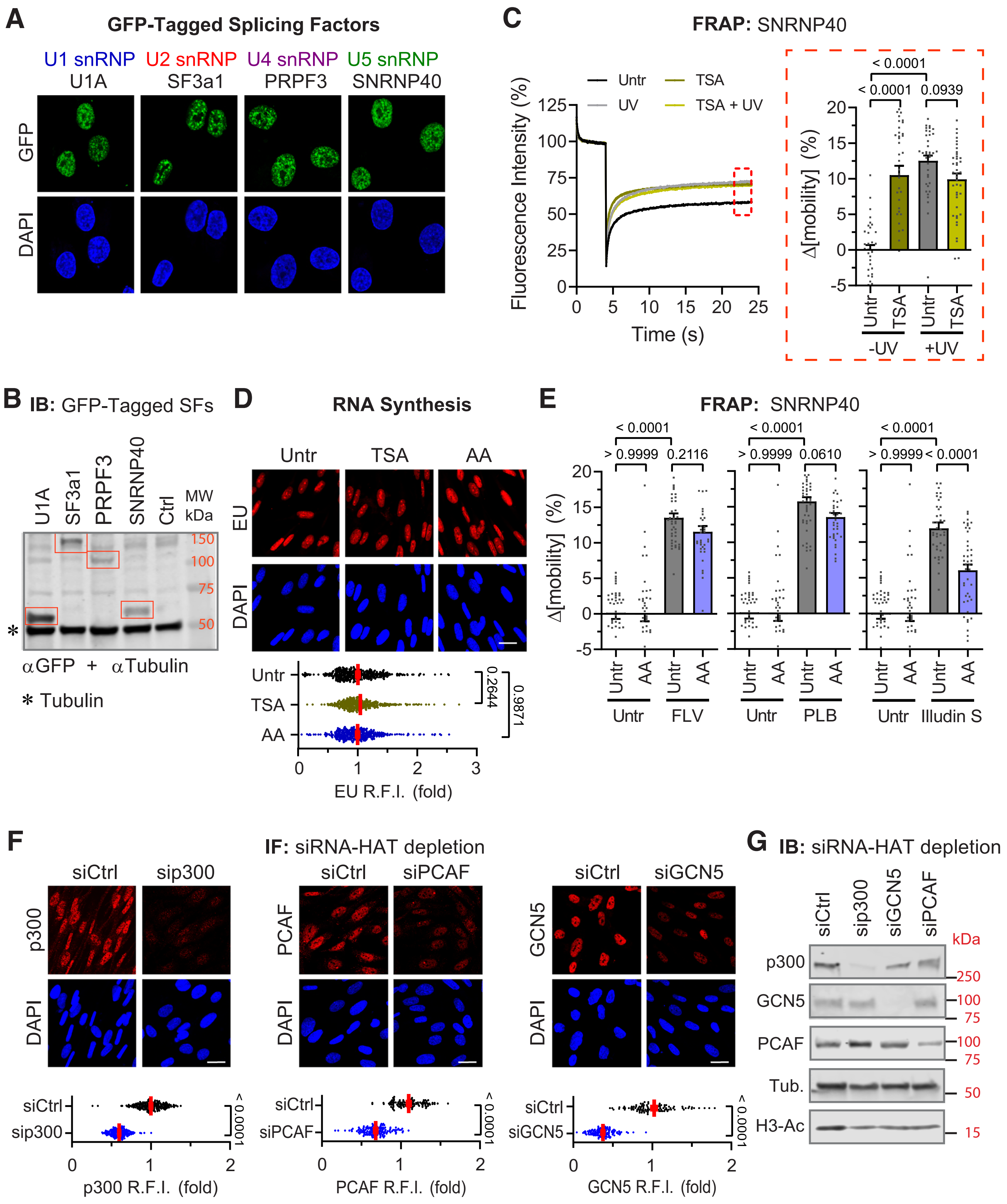
A
GFP-Tagged Splicing Factors
U1 snRNP U2 snRNP U4 snRNP U5 snRNP
U1A SF3a1 PRPF3 SNRNP40
GFP
DAPI
B IB: GFP-Tagged SFs
U1A SF3a1 PRPF3 SNRNP40 Ctrl
MW kDa
150 100 75 50
αGFP + αTubulin
* Tubulin
C
FRAP: SNRNP40
Untr TSA UV TSA + UV
Fluorescence Intensity (%)
Time (s)
Δ[mobility] (%)
< 0.0001
< 0.0001 0.0939
Untr TSA Untr TSA
-UV +UV
D
RNA Synthesis
Untr TSA AA
EU
DAPI
0.2644
0.9871
EU R.F.I. (fold)
E
FRAP: SNRNP40
< 0.0001
> 0.9999 0.2116
> 0.9999 0.0610
> 0.9999 < 0.0001
Δ[mobility] (%)
Untr AA Untr AA
Untr FLV
Untr PLB
Untr Illudin S
F
IF: siRNA-HAT depletion
siCtrl sip300
siCtrl siPCAF
siCtrl siGCN5
p300
PCAF
GCN5
DAPI
< 0.0001
p300 R.F.I. (fold)
PCAF R.F.I. (fold)
GCN5 R.F.I. (fold)
G
IB: siRNA-HAT depletion
siCtrl sip300 siGCN5 siPCAF
kDa
p300
GCN5
PCAF
Tub.
H3-Ac
250 100 75 100 75 50 15

 © The Author(s)

Figure EV2. Validation of GFP-tagged spliceosomal proteins and histone acetyltransferase depletion efficiency.

(**A**) Expression of GFP-tagged splicing factors (SFs) from distinct snRNP complexes in Human Dermal Fibroblasts (HDFs). Representative fluorescence microscopy images show characteristic speckled nuclear distribution of GFP-tagged SFs. Nuclei were stained with DAPI. (**B**) Immunoblot analysis of stably expressed snRNP-specific GFP-tagged SFs in HDFs. Tubulin served as a loading control. Depicted in each SNRNP40-GFP FRAP curve is the mean fluorescence recovery after background-correction and normalization to average pre-bleaching values ($n = 12$, representative experiment). Changes in mobility denoted as Δ[mobility] were calculated as the SNRNP40-GFP fluorescence in treated - fluorescence in untreated, non-irradiated cells at 20-21 s post photobleaching (indicated by the rectangle in the left panel) and plotted in the graph next to the FRAP curves. (**C**) SNRNP40-GFP mobility assayed by FRAP in quiescent HDFs under control conditions, following UV irradiation (20 J/m², 30 min), or chromatin hyperacetylation induced by TSA (1 µM, 3 h). Mean FRAP recovery curves are shown after background correction and normalization to average pre-bleaching values ($n = 12$, representative experiment). Mobility changes (Δ[mobility]) were calculated as the difference in normalized fluorescence intensity between UV irradiated and untreated non-irradiated cells at 20-21 s post photobleaching (indicated by the rectangle in the left panel) and plotted in the graph next to the FRAP curves. (**D**) Chromatin acetylation does not impact global RNA synthesis. Representative immunofluorescence images and quantification of nascent RNA synthesis (EU incorporation) in quiescent HDFs treated with TSA (1 µM, 3 h) or Anacardic Acid (AA; 10 µM, 2 h). Mean fluorescence intensities were normalized to untreated controls. Nuclei were stained with DAPI. Scale bars: 20 µm. (**E**) HAT inhibition impairs spliceosome mobilization by transcription-blocking lesions (TBLs) but not by pharmacological disruption of transcription initiation or spliceosome assembly. SNRNP40-GFP FRAP was performed in quiescent HDFs treated with the transcription initiation inhibitor Flavopiridol (FLV - 1 µM, 1 h), the spliceosome maturation inhibitor Pladienolide B (PLB - 1 µM, 1 h), or the TBL inducing drug Illudin S (25 ng/ml, 1 h). Treatments were administered in the presence or absence of AA (10 µM, 2 h). Calculated changes in SNRNP40-GFP mobility are shown. (**F**, **G**) Efficiency of HAT depletion by siRNA. (**F**) Representative immunofluorescence images and quantifications showing decreased fluorescence intensities of p300, PCAF, and GCN5 following siRNA transfection. Nuclei stained with DAPI. Scale bars: 20 µm. (**G**) Immunoblots showing protein levels of p300, PCAF, GCN5, and Histone H3 acetylation in siRNA-transfected cells. Tubulin is shown as loading control. Data information: Mean ± SEM of: (**C**) $n = 34/37/39/42$ (left to right), (**D**) $n = 402/411/398$ (top to bottom), (**E**) $n = 40/10/15/35$, 40/40/37/38, and 43/40/42/42 (left to right); all from three (**C**, **E**) or four (**D**) biological replicates. (**F**) $n = 253/262$, *119/134*, and 107/138 (left to right), from two biological replicates. (**C–E**) Brown–Forsythe and Welch's ANOVA, Games–Howell's multiple comparisons test; (**F**) unpaired two-tailed *t*-test with Welch's correction.

© The Author(s)

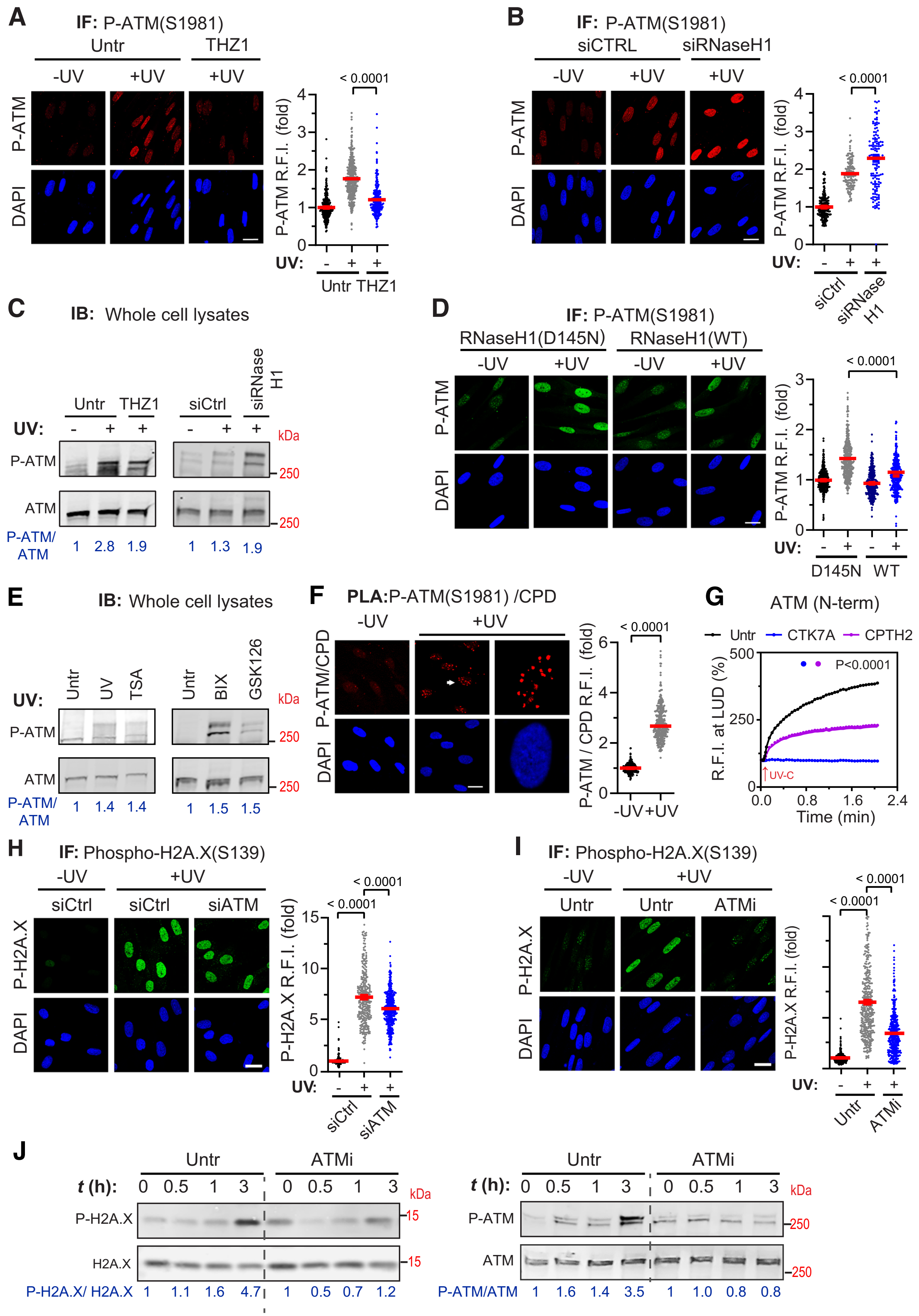
A
IF: P-ATM(S1981)
Untr
THZ1
-UV
+UV
+UV
P-ATM
DAPI
< 0.0001
P-ATM R.F.I. (fold)
UV:
Untr THZ1
B
IF: P-ATM(S1981)
siCTRL
siRNaseH1
P-ATM R.F.I. (fold)
siCtrl
siRNase H1
C
IB: Whole cell lysates
Untr THZ1 siCtrl siRNase H1
UV:
P-ATM
ATM
kDa
250
P-ATM/ ATM
1 2.8 1.9 1 1.3 1.9
D
IF: P-ATM(S1981)
RNaseH1(D145N)
RNaseH1(WT)
D145N WT
E
IB: Whole cell lysates
Untr UV TSA Untr BIX GSK126
P-ATM/ ATM
1 1.4 1.4 1 1.5 1.5
F
PLA:P-ATM(S1981) /CPD
P-ATM/CPD
P-ATM / CPD R.F.I. (fold)
-UV+UV
G
ATM (N-term)
Untr
CTK7A
CPTH2
P<0.0001
R.F.I. at LUD (%)
UV-C
Time (min)
H
IF: Phospho-H2A.X(S139)
siCtrl
siATM
P-H2A.X
P-H2A.X R.F.I. (fold)
I
IF: Phospho-H2A.X(S139)
Untr
ATMi
J
t (h): 0 0.5 1 3 0 0.5 1 3
P-H2A.X
H2A.X
15
P-H2A.X/ H2A.X 1 1.1 1.6 4.7 1 0.5 0.7 1.2
P-ATM
ATM
P-ATM/ATM 1 1.6 1.4 3.5 1 1.0 0.8 0.8

 © The Author(s)

Figure EV3. Transcription- and R-loop-dependent ATM activation in response to UV-induced DNA damage.

(**A–D**) UV-induced ATM activation requires active transcription and R-loop accumulation. Representative immunofluorescence images and quantification of UV-induced (40 J/m², 2 h) autophosphorylation of ATM in quiescent HDFs under the following conditions: (**A**, **C**) ± transcription inhibition by THZ1 (10 µM, 3 h); (**B**, **C**) following siRNA-mediated depletion of the R-loop hydrolase RNaseH1; (**D**) after doxycycline-induced expression of mCherry-tagged wild-type RNaseH1 or catalytically inactive D145N mutant. (**E**) Immunoblot analysis of ATM activation in response to chromatin-modifying treatments: UV irradiation (20 J/m², 2 h), HDAC inhibitor TSA (1 µM, 2 h), or HMT inhibitors BIX01294 and GSK126 (10 µM, 2 h each). All treatments induce chromatin acetylation typical of relaxed chromatin. (**F**) Representative images and quantification of Proximity Ligation Assays (PLA) detecting co-localization of auto-phosphorylated ATM (active) and UV-induced CPDs in quiescent HDFs. Arrow indicates magnified cell. (**G**) HAT inhibition impairs ATM recruitment to UV-C laser-damaged chromatin. Quiescent HDFs stably expressing GFP-tagged ATM N-terminal domain (aa 2–1314) were treated with HAT inhibitors prior to UV-C laser microirradiation (256 nm). Fluorescence intensities at the irradiation site were normalized to pre-irradiation levels. (**H–J**) ATM activity contributes to UV-induced phosphorylation of histone H2A.X. Representative images and quantifications of phospho-H2A.X in (**H**) siATM-depleted and (**I**) ATM-inhibitor treated cells. (**J**) Immunoblots showing phospho-H2A.X and ATM autophosphorylation at indicated times post-UV irradiation. Data information: Mean ± SEM of (left to right): (**A**) $n =$ 363/438/287; (**B**) 189/127/140; (**D**) 367/355/370/349; (**F**) 164/194; (**H**) 272/291/417; and (**I**) 338/443/393 cells; from three biological replicates (**A**, **D**, **H**, **I**) or two biological replicates (**B, F**). Brown–Forsythe and Welch's ANOVA with Games–Howell's multiple comparisons test (**A**, **B**, **D**, **H**, **I**) or unpaired two-tailed *t*-test with Welch's correction (**F**). (**G**) Mean ± SEM of $n = 41$ (untreated), 43 (CTK7A-treated), and 35 (CPTH2-treated) cells from three biological replicates; repeated-measures two-way ANOVA.

© The Author(s)

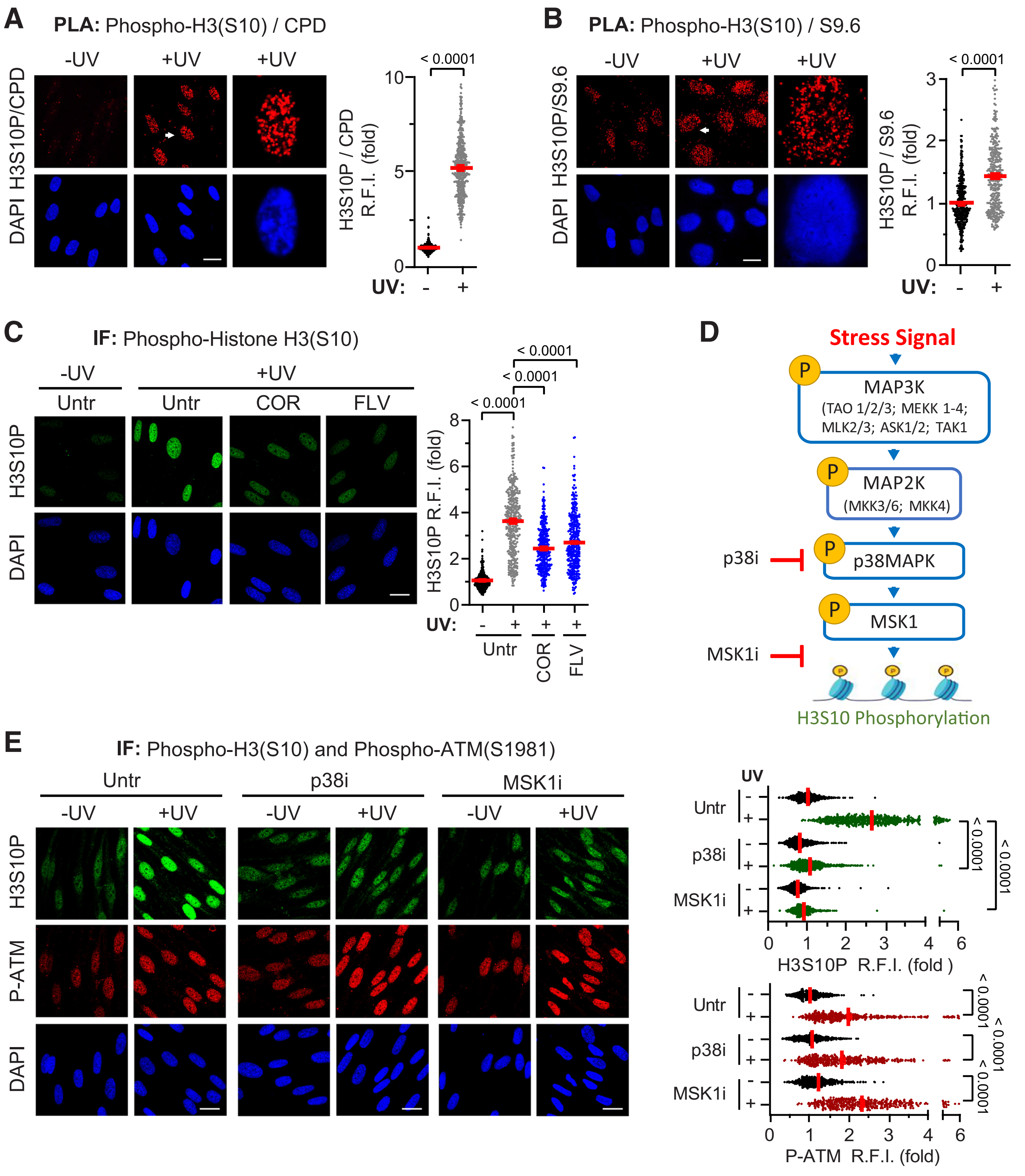
A
PLA: Phospho-H3(S10) / CPD
-UV
+UV
+UV
DAPI H3S10P/CPD
H3S10P / CPD R.F.I. (fold)
< 0.0001
10
5
0
UV: - +
B
PLA: Phospho-H3(S10) / S9.6
-UV
+UV
+UV
DAPI H3S10P/S9.6
H3S10P / S9.6 R.F.I. (fold)
< 0.0001
3
2
1
0
UV: - +
C
IF: Phospho-Histone H3(S10)
-UV
+UV
Untr
Untr
COR
FLV
H3S10P
DAPI
H3S10P R.F.I. (fold)
< 0.0001
< 0.0001
< 0.0001
8
6
4
2
0
UV: - + + +
Untr
COR
FLV
D
Stress Signal
P
MAP3K
(TAO 1/2/3; MEKK 1-4; MLK2/3; ASK1/2; TAK1
MAP2K
(MKK3/6; MKK4)
p38i
p38MAPK
MSK1i
MSK1
H3S10 Phosphorylation
E
IF: Phospho-H3(S10) and Phospho-ATM(S1981)
Untr
p38i
MSK1i
-UV
+UV
H3S10P
P-ATM
DAPI
UV
Untr
p38i
MSK1i
< 0.0001
< 0.0001
0 1 2 3 4 6
H3S10P R.F.I. (fold)
< 0.0001
< 0.0001
< 0.0001
0 1 2 3 4 6
P-ATM R.F.I. (fold)

 © The Author(s)

Figure EV4. Transcription-dependent phosphorylation of Histone H3S10 at UV-damaged, R-loop-containing chromatin.

(**A**, **B**) Phosphorylation of Histone occurs near UV-induced DNA damage and R-loop–rich chromatin. Representative PLA images and quantification of phosphorylation proximal to: (**A**) UV-induced CPDs and (**B**) RNA:DNA hybrids in detergent-extracted nuclei of quiescent UV irradiated (30 J/m², 1 h). Nuclei stained with DAPI. Scale bars: 20 µm. (**C**) Phosphorylation requires active transcription. Immunofluorescence images and quantification of H3S10P in quiescent HDFs treated with transcription inhibitors Flavopiridol (FLV, 1 µM, 4 h) or Cordycepin (COR, 10 µM, 4 h) prior to UV irradiation (40 J/m^2, 2 h). (**D**) Schematic of the p38–MSK1 signaling cascade mediating phosphorylation in response to stress, indicating pharmacological inhibitors used in this study. (**E**) UV-induced phosphorylation is dispensable for ATM activation. Representative images and quantification of P and auto-phosphorylated ATM in control and UV-irradiated quiescent HDFs with or without p38 (SB203580, 20 µM, 4 h) or MSK1 (SB747651A, 25 µM, 4 h) inhibition. Nuclei stained with DAPI. Scale bars: 20 µm. Data information: Mean ± SEM of (left to right): (**A**) $n = 569/484$; (**B**) 392/339; and (**C**) 351/348/357/374 cells from three biological replicates. Unpaired two-tailed *t* test with Welch's correction (**A**, **B**), or Brown–Forsythe and Welch's ANOVA with Games–Howell's multiple comparisons test (**C**). (**E**) Mean ± SEM of (top to bottom): $n = 495/532/539/540/477/479$ cells for both H3S10P and P-ATM detection from four biological replicates. Brown–Forsythe and Welch's ANOVA with Games–Howell's multiple comparisons test.

© The Author(s)

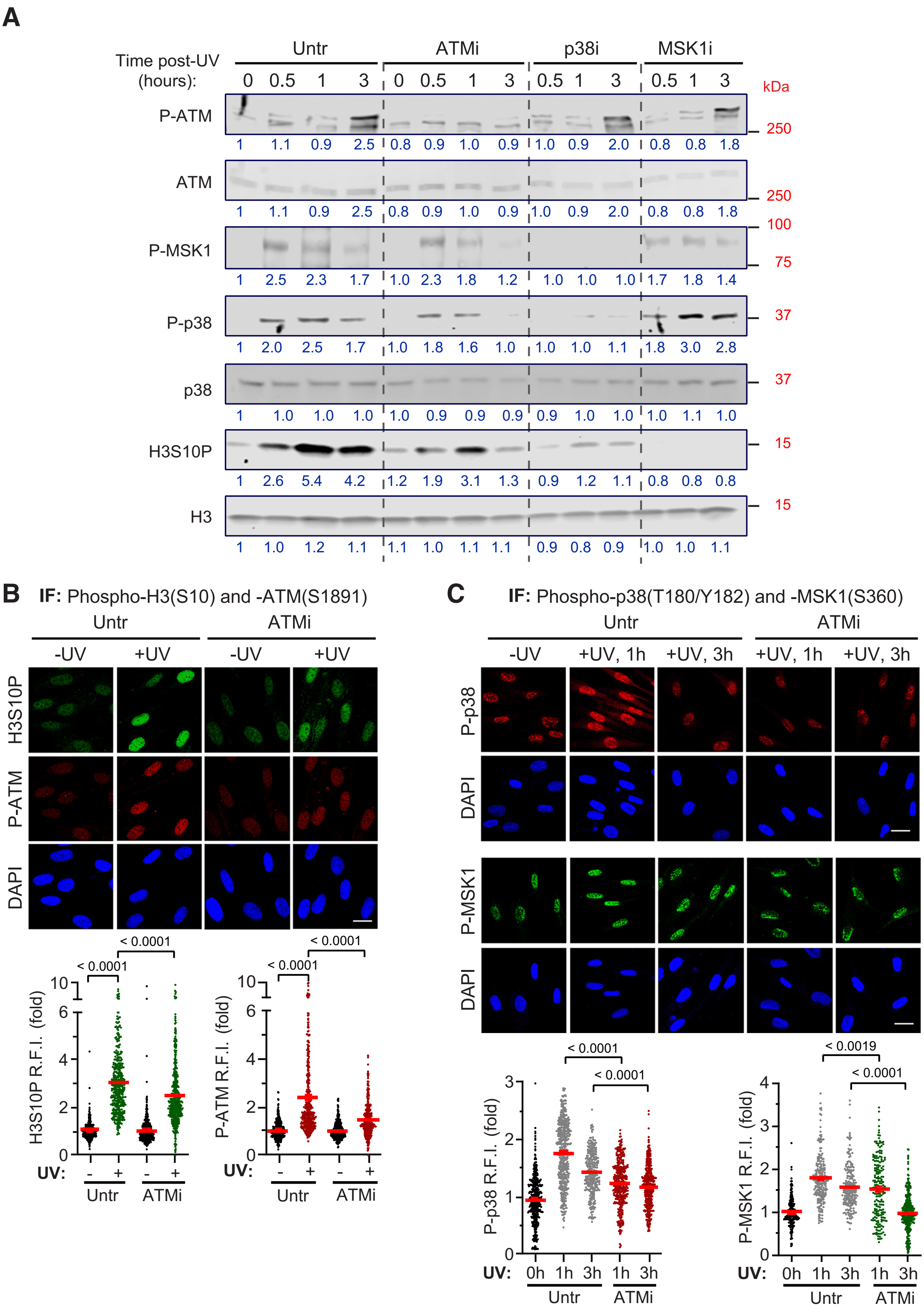
A
Time post-UV (hours):
Untr
ATMi
p38i
MSK1i
0 0.5 1 3 0 0.5 1 3 0.5 1 3 0.5 1 3
kDa
P-ATM
1 1.1 0.9 2.5 0.8 0.9 1.0 0.9 1.0 0.9 2.0 0.8 0.8 1.8
250
ATM
1 1.1 0.9 2.5 0.8 0.9 1.0 0.9 1.0 0.9 2.0 0.8 0.8 1.8
250
100
P-MSK1
1 2.5 2.3 1.7 1.0 2.3 1.8 1.2 1.0 1.0 1.0 1.7 1.8 1.4
75
P-p38
1 2.0 2.5 1.7 1.0 1.8 1.6 1.0 1.0 1.0 1.1 1.8 3.0 2.8
37
p38
1 1.0 1.0 1.0 1.0 0.9 0.9 0.9 0.9 1.0 1.0 1.0 1.1 1.0
37
H3S10P
1 2.6 5.4 4.2 1.2 1.9 3.1 1.3 0.9 1.2 1.1 0.8 0.8 0.8
15
H3
1 1.0 1.2 1.1 1.1 1.0 1.1 1.1 0.9 0.8 0.9 1.0 1.0 1.1
15
B
IF: Phospho-H3(S10) and -ATM(S1891)
Untr
ATMi
-UV
+UV
-UV
+UV
H3S10P
P-ATM
DAPI
< 0.0001
< 0.0001
H3S10P R.F.I. (fold)
10
6
4
2
0
UV: - + - +
Untr ATMi
< 0.0001
< 0.0001
P-ATM R.F.I. (fold)
10
6
4
2
0
UV: - + - +
Untr ATMi
C
IF: Phospho-p38(T180/Y182) and -MSK1(S360)
Untr
ATMi
-UV
+UV, 1h
+UV, 3h
+UV, 1h
+UV, 3h
P-p38
DAPI
P-MSK1
DAPI
< 0.0001
< 0.0001
P-p38 R.F.I. (fold)
3
2
1
0
UV: 0h 1h 3h 1h 3h
Untr ATMi
< 0.0019
< 0.0001
P-MSK1 R.F.I. (fold)
4
3
2
1
0
UV: 0h 1h 3h 1h 3h
Untr ATMi

 © The Author(s)

Figure EV5. ATM activity is required for UV-induced activation of the p38–MSK1– phosphorylation cascade.

(**A**) Immunoblot validation of pharmacological inhibition of ATM, p38, and MSK1, and their effects on downstream targets regulating H3S10 phosphorylation. Quiescent HDFs were treated with KU-55933 (10 μM, 2 h), SB203580 (20 μM, 4 h), or SB747651A (25 μM, 4 h) prior to UV irradiation (40 J/m^2). Whole cell extracts collected at indicated time-points post irradiation were analyzed by immunoblotting. All shown proteins were detected in the same membrane. (**B**) ATM activity promotes UV-induced phosphorylation of histone H3S10. Representative immunofluorescence images and quantification of phosphorylated H3S10 and auto-phosphorylated ATM in quiescent HDFs ± UV irradiation (40 J/m², 2 h) with or without ATM inhibitor KU-55933 (10 μM, 2 h) pre-treatment. (**C**) UV-induced activation of the p38/MSK1 pathway depends on ATM. Representative images and quantification of phosphorylated p38 and MSK1 with or without KU-55933 pre-treatment prior to UV irradiation (40 J/m², 2 h). Data information: Mean ± SEM of (left to right): (**B**) $n = 430/398/411/430$ (H3S10P and P-ATM); (**C**) 443/456/289/363/431 (P-p38); and 216/222/206/194/305 (P-MSK1) cells from three biological replicates. Brown–Forsythe and Welch's ANOVA with Games–Howell's multiple comparisons test.

© The Author(s)
